# Deterministic dynamics of distributional multi-agent reinforcement learning

**DOI:** 10.1371/journal.pcbi.1014723

**Published:** 2026-09-23

**Authors:** Clémence Bergerot, Pawel Romanczuk, Wolfram Barfuss

**Affiliations:** 1 Department of Biology, Humboldt Universität zu Berlin, Berlin, Germany; 2 Charité-Universitätsmedizin Berlin, Einstein Center for Neurosciences Berlin, Berlin, Germany; 3 Science of Intelligence, Research Cluster of Excellence, Berlin, Germany; 4 Transdisciplinary Research Area Sustainable Futures, University of Bonn, Bonn, Germany; 5 Center for Development Research, University of Bonn, Bonn, Germany; 6 Institute for Food and Resource Economics, University of Bonn, Bonn, Germany; Durham University, UNITED KINGDOM OF GREAT BRITAIN AND NORTHERN IRELAND

## Abstract

Understanding how cognition shapes behavior across contexts remains a fundamental challenge for many disciplines. In particular, for the optimism heuristic–i.e., the tendency to overweight positive (relative to negative) information–knowledge remains fragmented, with models developed in specific domains in isolation. Here, we present a unifying computational framework by deriving the deterministic dynamics of distributional multi-agent reinforcement learning. Our approach discretizes return distributions through a finite set of neurons, consistent with recent empirical findings on distributional coding in the brain. We validate our framework by reproducing established results across three iconic domains spanning individual bandit choice under resource variability, social coordination, and risky choice. Beyond validation, we uncover novel interactions among optimism, return discretization, and temporal discounting. Specifically, we identify conditions under which return discretization generates choice hysteresis and, in extreme parameter regimes, inescapable perseveration. We further reveal “individual dilemmas”: circumstances where agents gravitate toward suboptimal yet stable strategies, offering a mechanistic explanation for incoherent choice patterns. Our framework bridges neuroscience, psychology, and collective behavior, enabling empirically testable hypotheses about how cognitive biases propagate from individual cognition to social outcomes in complex environments.

## Author summary

How do cognitive heuristics shape individual decisions and the collective outcomes that emerge when many agents interact? This question connects psychology, neuroscience, and artificial intelligence, yet computational models have developed separately across these fields. Here we present DDRL, a framework that combines distributional reinforcement learning, in which agents learn the full spread of possible outcomes rather than their average, with deterministic learning dynamics that yield mathematically tractable trajectories in strategy space. DDRL is grounded in neuroscientific evidence: dopaminergic neurons encode outcome distributions rather than expected values alone. We illustrate DDRL with an optimism heuristic across three domains: bandit choice under resource variability, social coordination, and intertemporal risky choice. In each case, DDRL reproduces established results. Beyond replication, DDRL makes two novel predictions. Return discretization generates bistable regimes, in which agents settle into qualitatively different behaviors depending on their history. Under certain combinations of optimism and temporal discounting, agents can become locked into an individual dilemma, in which the better strategy is no longer a stable attractor. Together, these results provide a mechanistic route from neural reward encoding to emergent collective behavior, connecting individual cognitive heuristics to social outcomes in a biologically grounded and analytically tractable framework.

## 1. Introduction

Developing biologically grounded models of learning and collective decision-making is a shared priority across biology, economics, artificial intelligence, and sustainability science [1–7]. Reinforcement learning (RL) is a promising framework for this effort [8–11]: agents improve their decision strategies through trial and error, and dopaminergic neurons in the brain signal reward-prediction errors in a manner that closely matches RL learning rules [12,13]. In multi-agent settings, individual learning dynamics give rise to collective behavior — emergent outcomes arising from individual learning in a shared environment, without requiring explicit communication between agents — opening a route to mechanistic models of social decision-making [11,14,15]. Standard RL approaches, however, derive behavior from a single quantity: the expected return. Human and animal decision-making systematically deviates from this expectation-based framework — exhibiting biases and heuristics that are difficult to capture while preserving analytical tractability in a multi-agent setting with changing environmental contexts.

Behavioral heuristics—cognitive shortcuts that enable effective decisions in uncertain environments—offer one productive lens for this challenge [16,17]. Among the best-studied is the optimism heuristic: the tendency to weight positive over negative information. At the individual level, asymmetric RL documents it in humans, mice, and monkeys [18–21], using a higher learning rate for positive prediction errors than for negative ones. At the collective level, optimistic coordination algorithms have enhanced performance across a range of multi-agent tasks [22–26]. These efforts, however, have been developed in separate disciplinary traditions. Optimism and pessimism are treated as phenomenological hyperparameters: effective, but not grounded in a mechanistic account of how reward outcomes are represented.

Distributional RL (DistRL) provides the mechanistic substrate this picture is missing [27]. Rather than estimating the expected return, distributional agents learn the full distribution of possible returns. Neural signatures of this computation have been found in the dopaminergic system [28], connecting DistRL to the biological machinery of reward processing. Within DistRL, optimism and pessimism arise naturally: an agent selects an upper quantile of the return distribution [29,30] or distorts its shape [31,32], rather than adjusting learning rates. DistRL has been applied primarily in single-agent machine-learning settings; a deterministic formulation for multi-agent collective dynamics does not yet exist.

Deterministic RL (DetRL) dynamics provide analytical tractability in collective settings [33,34]. In DetRL, stochastic fluctuations in the learning process are integrated out: strategies are updated as deterministic functions of expected rewards and state transitions. With a formalism analogous to evolutionary game theory [35,36], this approach enables exact characterization of fixed points, their stability, and bifurcations of collective learning. Prior DetRL work has revealed how caring for the future shapes cooperation in social-ecological dilemmas [37] and how stochastic noise drives coordination [38]. DetRL, however, operates on scalar Q-values—point estimates of expected return. The distributional structure of reward outcomes is absent.

Here, we derive the deterministic dynamics of distributional multi-agent RL, introducing a framework we call DDRL (Section 3.1). DDRL combines the distributional representation of DistRL with the deterministic update dynamics of DetRL. Agents discretize the return spectrum using a finite population of reward neurons, consistent with empirical findings on distributional coding [28]. We apply our framework to the optimism heuristic. Optimism is implemented as a distortion of the learned return distribution. Our deterministic dynamics describe the expected trajectory of a learning agent, averaging out the intrinsic noise of the algorithmic learning update due to stochastic strategies, transitions, and rewards. DDRL is therefore a novel method of analysis for distributional multi-agent RL [39]. We validate it by testing whether its predictions (the fixed points, their stability, and bifurcations) reproduce established behavioral findings. We do so across three canonical task domains: a bandit choice task under resource variability (Section 3.2), a social coordination game (Section 3.3), and an intertemporal risky choice problem (Section 3.4). Our results replicate established behavioral findings in each domain. They also reveal novel interactions among optimism, return discretization, and temporal discounting—specifically, bistable strategy regimes and a form of incoherent individual choice we term the individual dilemma. This constitutes a step toward a unified, biologically grounded framework for studying behavior emerging from cognition across collective decision-making contexts.

## 2. Methods

### 2.1. Stochastic games

Stochastic games, also known as Markov games, are defined by the elements ⟨N,S,A,T, R⟩. N∈N agents reside in an environment of Z∈N states $S=(S_{1},...,S_{Z})$. In each state *s*, each agent i∈N={1,...,N} has M∈N available actions $A^{i}=(A_{1}^{i},...,A_{M}^{i})$ to choose from. $A={⨂}_{i}A^{i}$ is the joint-action set where ${⨂}_{i}$ denotes the Cartesian product over the sets indexed by *i*. Time advances in discrete steps, and agents choose their actions simultaneously. A joint action is denoted by $a=(a^{1},...,a^{N})\in A$. With $a^{-i}=(a^{1},...,$ $a^{i-1},$ $a^{i+1},$ …, $a^{N})$ we denote the joint action except agent *i*’s, and we write the joint action in which agent *i* chooses $a^{i}$ and all other agents choose $a^{-i}$ as $a^{i}a^{-i}$. We choose an equal number of actions for all states and agents out of notational convenience.

The **transition** tensor T:S×A×S→[0,1] determines the probabilistic state change. $T^{s,a,\overset{´}{s}}$ is the transition probability from current state *s* to next state $\overset{´}{s}$ under joint action a.

The **reward** tensor R:N×S×A×S→R maps the triple of current state *s*, joint action a and next state $\overset{´}{s}$ to an immediate reward scalar for each agent. $R^{i,s,a,\overset{´}{s}}$ is the reward agent *i* receives.

At each time step, actions are chosen from the joint **strategy** tensor X:N×S×A→[0,1]. In state *s*, agent *i* chooses action $a^{i}$ with probability $X^{i,s,a^{i}}$.

Each agent *i* aims for a strategy $X^{i,s,a^{i}}$ to maximize their **gain**,


$$\begin{array}{c} G_{t}^{i}=(1-\gamma^{i})\sum_{k=0}^{\infty }(\gamma^{i}{)}^{k}r_{t+k}^{i} \end{array}$$ (1)

where $r_{t+k}^{i}$ denotes the reward agent *i* obtained at time step *t* + *k*. The discount factor, $\gamma^{i}\in [0,1)$, denotes how much agen*t i* cares for future rewards.

### 2.2. Reinforcement learning

Reinforcement learning is a trial-and-error method of mapping situations to actions to maximize a numerical reward signal [9]. When rewards are a delayed consequence of current actions, so-called temporal-difference or reward-prediction learning has been particularly influential. This type of learning summarizes the difference between value estimates from past and present experiences into a reward-prediction error, which is then used to adapt the current behavior to gain more rewards over time. There also exist remarkable similarities between computational reinforcement learning and the results of neuroscientific experiments [13]. Dopamine conveys reward-prediction errors to brain structures where learning and decision-making occur [12].

At time step *t*, in state *s*, agent *i* evaluates action $a^{i}$ to be of quality $Q_{t}^{i,s,a^{i}}$. Learning then means updating these state-action value estimates, $Q_{t}^{i,s,a^{i}}$, after selecting action $a_{t}^{i}$ and having observed state $s_{t}$ according to


$$\begin{array}{c} Q_{t+1}^{i,s_{t},a_{t}^{i}}=Q_{t}^{i,s_{t},a_{t}^{i}}+\alpha^{i}E_{t}^{i,s_{t},a_{t}^{i}}, \end{array}$$ (2)

where $\alpha^{i}\in [0,1]$ denotes agent *i*’s learning rate, which regulates how much new information the agent uses for the update, and $E_{t}^{i,s_{t},a_{t}^{i}}$ the current temporal-difference or reward-prediction error. The expression of the temporal-difference error depends on the chosen RL algorithm.

In this work, we focus on two different RL variants: SARSA learning, and Actor-Critic learning. SARSA learning takes into account the five elements of the current State, current Action, Reward, next State, and next Action for one learning update step. In Actor-Critic learning, an approximation of the State-Action value gets “criticized” by (i.e., compared against) an approximation of the state value. Another well-known variant is Q-learning. A comparison of these three variants can be found in [33].

In SARSA learning, the temporal-difference error is expressed as:


$$\begin{array}{c} E_{t}^{i,s_{t},a_{t}^{i}}=(1-\gamma^{i})r_{t}^{i}+\gamma^{i}Q_{t}^{i,s_{t+1},a_{t+1}^{i}}-Q_{t}^{i,s_{t},a_{t}^{i}}. \end{array}$$ (3)

In Actor-Critic learning, by contrast, the temporal-difference error retains the same one-step bootstrap target $(1-\gamma^{i})r_{t}^{i}+\gamma^{i}Q_{t}^{i,s_{t+1},a_{t+1}^{i}}$ as in SARSA, but replaces the baseline $Q_{t}^{i,s_{t},a_{t}^{i}}$ with the state value $V_{t}^{i,s_{t}}$,


$$\begin{array}{c} E_{t}^{i,s_{t},a_{t}^{i}}=(1-\gamma^{i})r_{t}^{i}+\gamma^{i}Q_{t}^{i,s_{t+1},a_{t+1}^{i}}-V_{t}^{i,s_{t}}. \end{array}$$ (4)

The state value at time *t*, $V_{t}^{i,s_{t}}$, quantifies the overall quality of state *s* at time *t*, regardless of the chosen action $a_{t}$. In Actor-Cri*t*ic learning, it is separately updated according to:


$$\begin{array}{c} V_{t+1}^{i,s_{t}}=V_{t}^{i,s_{t}}+\alpha^{i}E_{t}^{i,s_{t},a_{t}^{i}}. \end{array}$$ (5)

Agents select actions based on the current state-action value beliefs $Q_{t}^{i,s_{t},a_{t}^{i}}$, balancing exploitation (i.e., choosing the action of maximum quality) and exploration (i.e., selecting lower-quality actions to learn more about the environment). We employ the widely used Boltzmann policy function. The probability of choosing action $a^{i}$ under observing state *s* is


$$\begin{array}{c} X_{t}^{i,s,a^{i}}=\frac{\exp\left(\beta^{i}Q_{t}^{i,s,a^{i}}\right)}{\sum_{b\in A^{i}}\exp\left(\beta^{i}Q_{t}^{i,s,b}\right)}, \end{array}$$ (6)

where the *intensity-of-choice* parameters, $\beta^{i}\in R^{+}$, regulate the exploration-exploitation trade-off. For high $\beta^{i}$, agents exploit their learned knowledge about the environment, leaning toward actions with high estimated state-action values. For low $\beta^{i}$, agents are more likely to deviate from these high-value actions to explore the environment further with the chance of finding actions that eventually lead to even higher values. Our choice of the Boltzmann policy is motivated by the maximum-entropy principle [40]. It is the distribution over actions that maximizes entropy subject to a constraint on the expected Q-value $\sum_{a^{i}}X_{t}^{i,s,a^{i}}Q_{t}^{i,s,a^{i}}$ or equivalently, the distribution that maximizes the expected Q-value subject to a constraint on entropy, with $\beta^{i}$ the Lagrange multiplier coupling the two objectives [41]. It also resembles the idea of reward-matching behavior in animal and human choice [42,43]. From a modeling standpoint, the Boltzmann policy is particularly convenient for the deterministic dynamics framework of Section 2.4: because the strategy update depends only on differences in Q-values across actions, action-constant quantities cancel, substantially simplifying the dynamics. Alternative exploration policies such as ε-greedy can also be combined with strategy averaging [38], but at the cost of a larger dynamical state space; more elaborate schemes such as UCB, which track uncertainty explicitly, complicate the dynamics further still, to the point of being analytically intractable unless the exploration process itself is the primary object of study.

### 2.3. Distributional reinforcement learning

Distributional approaches to RL (DistRL) in artificial intelligence and machine learning research were pioneered by [44,45] and recently gained wider prominence [27]. DistRL diverges from the classical approach insofar as learning agents do not compute expected values, but rather the full distribution of outcomes. Therefore, the goal is not to compute an expected value or quality, but rather a **return distribution**, which is described by a distributional Bellman equation [27]:


$$\begin{array}{c} Q^{s,a}\overset{D}{=}(1-\gamma )R^{s,a}+\gamma Q^{\overset{´}{s},\overset{´}{a}} \end{array}$$ (7)

where $R^{s,a}$ is the reward for action *a* in state *s*, $\left(\overset{´}{s},\overset{´}{a}\right)$ is the next-state action, and Q is its random return. The (1−γ) factor reflects the normalized-gain convention used throughout this paper, $G_{t}=(1-\gamma )\sum_{k\geq 0}\gamma^{k}r_{t+k}$.

Over the past years, DistRL researchers have used various supports for discretizing the distributions. In categorical DistRL, the discretization support is a set $\left\{z_{i}=V_{\min}+i\Delta z:0\leq i<N\right\}$ of *N* categorical “atoms,” which are fixed and evenly spaced (with $\Delta z=\frac{V_{\max}-V_{\min}}{N-1}$) [27]. Later versions of DistRL algorithms rely on the distributions’ statistics as discretization support: the atoms are not fixed anymore, but are updated so as to converge towards quantiles or expectiles of the target distribution [46].

In this work, we will use a categorical discretization support because it maps naturally onto a fixed population of neurons, each tuned to a specific return value. In standard categorical DistRL, atoms span the range of possible returns $\left[V_{\min},V_{\max}\right]$. Under the normalized gain convention (Eq. 1) used throughout this paper, returns are convex combinations of rewards and therefore lie in $\left[r_{1},r_{n}\right]$. Our atoms, defined at reward values $r_{1},...,r_{n}$, cover this range exactly. Quantile- and expectile-based representations use adaptive atoms that shift during learning [46,47], which improves approximation quality but would render the deterministic dynamics derivation of this paper incompatible, as the representational state itself would become time-varying.

### 2.4. Deterministic learning dynamics

Deterministic dynamics of RL remove noise from the learning process. Hence, the agents’ strategies over time can be computed and visualized as deterministic trajectories in phase space. To this end, we use temporal-difference RL in the deterministic limit (DetRL), which was developed by Barfuss *et al.*[33],. This method uses strategy-averages as a way to remove the noise from the learning dynamics: the prediction error is averaged over all strategies and state transitions. With a formalism analogous to evolutionary game theory [35,36], deterministic RL dynamics yield analytically tractable trajectories in strategy space and enable the exact characterization of fixed points, their stability, and bifurcations — properties we exploit to identify bistable choice regimes in Section 3.4. More precisely, SARSA dynamics with a Boltzmann strategy function are analogous to the replicator-mutator equation [33] — a mutation term prevents strategies from collapsing to a pure strategy — whereas Actor-Critic dynamics are analogous to the pure replicator equation [35], which drives strategies toward deterministic fixed points. Thus, deterministic RL dynamics can be viewed as distinct level of analysis of multi-agent learning [39]. Stochastic RL algorithms form their noisy counterparts: the stationary distribution of the stochastic system concentrates around the stable fixed points of the deterministic part and spends negligible time near unstable ones, provided the noise is sufficiently weak and the deterministic dynamics have isolated stable fixed points rather than more complex attractors [38,39]. Under these conditions, the deterministic approximation captures average learning behavior while abstracting away intrinsic fluctuations. A related but conceptually distinct line of work is the ODE method for stochastic approximation [48,49], which links stochastic learning algorithms to a limiting ordinary differential equation in the vanishing step-size regime. There, the ODE serves as an asymptotic tool to establish convergence of the stochastic recursion to its invariant sets. These are precisely the conditions under which the link between deterministic dynamics and stochastic algorithms holds. In contrast, however, we do not take a vanishing learning-rate limit, but derive the induced deterministic update dynamics directly at finite step sizes (Section 3.1), treating their discrete-time behavior, including fixed points, stability, and bifurcations, as objects of intrinsic interest rather than as auxiliary constructs for convergence analysis.

First, we compute the different quantities we need to calculate the average temporal-difference error. As explained in Section 2.1, $X^{i,s,a^{i}}$ denotes the probability that agent *i* chooses action $a^{i}$ in state *s*; X:N×S×A→[0,1] denotes the joint strategy tensor.

We call $R_{X}^{i,s,a^{i}}$ the average reward given the joint policy X that agent *i* obtains by taking action $a^{i}$ in state *s*. More precisely, the average is taken over state transitions from *s* to $\overset{´}{s}$, and over other agents’ actions $a^{-i}$:


$$\begin{array}{c} R_{𝐗}^{i,s,a^{i}}=\sum_{\overset{´}{s}}\sum_{a^{-i}}\prod_{j\neq i}X^{j,s,a^{j}}T^{s,a,\overset{´}{s}}R^{i,s,a,\overset{´}{s}}. \end{array}$$ (8)

Similarly, we call ${\;}^{next}Q_{X}^{i,s,a^{i}}$ the average next-state Q-value given the joint policy X for agent *i* taking action $a^{i}$ in state *s*:


$$\begin{array}{c} {\;}^{next}Q_{𝐗}^{i,s,a^{i}}=\sum_{\overset{´}{s}}\sum_{a^{-i}}\prod_{j\neq i}X^{j,s,a^{j}}T^{s,a,\overset{´}{s}}\sum_{b^{i}}X^{i,\overset{´}{s},b^{i}}Q_{𝐗}^{i,\overset{´}{s},b^{i}}, \end{array}$$ (9)

where $Q_{𝐗}^{i,\overset{´}{s},b^{i}}$ denotes the average Q-value of action $b^{i}$ taken by agent *i* in state $\overset{´}{s}$, described by the recursive Bellman equation:


$$\begin{array}{c} Q_{𝐗}^{i,\overset{´}{s},b^{i}}=(1-\gamma^{i})R_{𝐗}^{i,\overset{´}{s},b^{i}}+\gamma^{i}\cdot {\;}^{next}Q_{𝐗}^{i,\overset{´}{s},b^{i}} \end{array}$$ (10)

Throughout this section, a subscript ${𝐗}_{t}$ denotes the strategy-averaged quantity evaluated at the joint strategy at time *t*. In the deterministic approximation, these are the true on-strategy expectations under ${𝐗}_{t}$, not estimates, so $Q_{{𝐗}_{t}}^{i,s,a^{i}}$ satisfies the above Bellman recursion exactly. The time index *t* is therefore inherited entirely through ${𝐗}_{t}$, and substitutions that combine $(1-\gamma^{i})R_{{𝐗}_{t}}+\gamma^{i}\;{\;}^{next}Q_{{𝐗}_{t}}$ into $Q_{{𝐗}_{t}}$ are identi*t*ies by construction. Unlike Eqs. 3 and 4, where *t* indexes the single state $s_{t}$ and action $a_{t}^{i}$ sampled at that step and only $Q_{t}^{i,s_{t},a_{t}^{i}}$ is updated, here $s,a^{i}$ range over every state-action pair. All get updated at each step *t* together as a consequence of the joint strategy upda*t*e (Eq. 13). The joint strategy ${𝐗}_{t}$ is the sole dynamic variable. Only it carries a time index *t*.

Therefore, for a SARSA agent, the average prediction error for agent *i* taking action $a^{i}$ in state *s* at time *t* is


$$\begin{array}{cc} E_{{𝐗}_{t}}^{i,s,a^{i}} & =(1-\gamma^{i})R_{{𝐗}_{t}}^{i,s,a^{i}}+\gamma^{i}\cdot {\;}^{next}Q_{{𝐗}_{t}}^{i,s,a^{i}}-\frac{1}{\beta^{i}}\log X_{t}^{i,s,a^{i}}\\ & =Q_{{𝐗}_{t}}^{i,s,a^{i}}-\frac{1}{\beta^{i}}\log X_{t}^{i,s,a^{i}}. \end{array}$$ (11)

where $\beta^{i}$ is the choice intensity, or inverse temperature, of agent *i*.

For an Actor-Critic agent, by contrast, the average Q-value $Q_{𝐗}^{i,s,a^{i}}$ is compared with the average state value $V_{𝐗}^{i,s}$ (the “critic”). Since this term is constant in action, we can omit it from the formula [33]. Thus, for an Actor-Critic agent, the average prediction error for agent *i* taking action $a^{i}$ in state *s* at time *t* is:


$$\begin{array}{cc} E_{{𝐗}_{t}}^{i,s,a^{i}} & =(1-\gamma^{i})R_{{𝐗}_{t}}^{i,s,a^{i}}+\gamma^{i}\cdot {\;}^{next}Q_{{𝐗}_{t}}^{i,s,a^{i}}\\ & =Q_{{𝐗}_{t}}^{i,s,a^{i}}. \end{array}$$ (12)

The combination of the Q-value update (Eq. 2) and the Boltzmann formula (Eq. 6) yields the deterministic update of strategy $X^{i,s,a^{i}}$, applied to every $\left(s,a^{i}\right)$ pair at once:


$$\begin{array}{c} X_{t+1}^{i,s,a^{i}}=\frac{X_{t}^{i,s,a^{i}}\exp[\alpha^{i}\beta^{i}E_{t}^{i,s,a^{i}}]}{\sum_{b^{i}}X_{t}^{i,s,b^{i}}\exp[\alpha^{i}\beta^{i}E_{t}^{i,s,b^{i}}]} \end{array}$$ (13)

## 3. Results

### 3.1. Theoretical framework

In this section, we derive deterministic distributional reinforcement learning dynamics. In order to model the optimism-pessimism heuristic, we apply deterministic learning dynamics to distributional RL. By doing so, we combine the best of both worlds: on the one hand, DetRL provides a transparent, reproducible, and computationally fast approach to RL; on the other hand, DistRL allows us to bias the return distribution of the learning dynamics and devise a simple model at the neuronal level.

Our approach is presented in Fig 1. The agent-environment interface is formalized as a Markov game ⟨N,S,A,T,R⟩ comprised of *N* agents, a set S of different states, a set A of available actions, a transition tensor *T*, and a reward tensor R. In DDRL, rewards and qualities are not computed as expected values, but as full distributions. These distributions are discretized according to a categorical support and a smoothing procedure. Then, the Q-return distribution is distorted according to a weighting algorithm, implementing the optimism heuristic.

**Fig 1:**
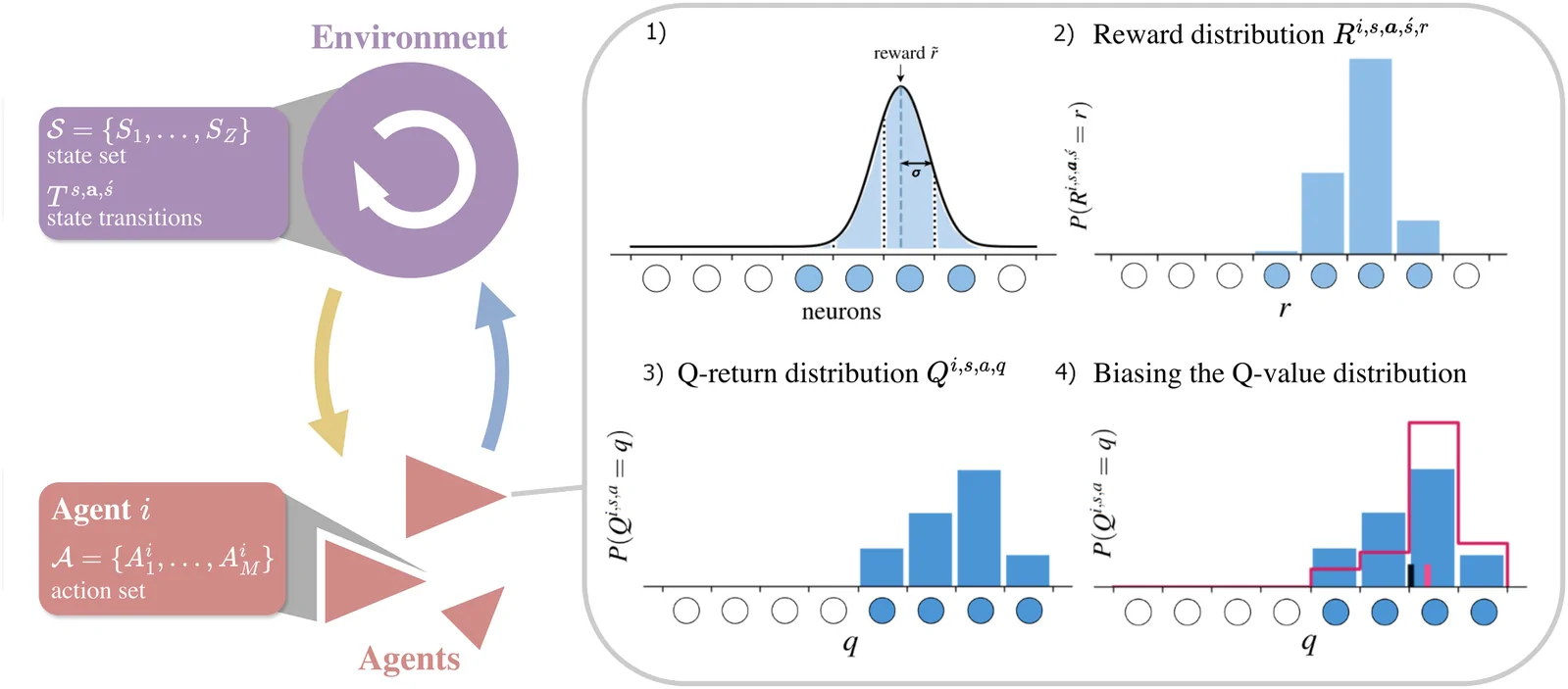
The agent-environment interface. *N* agents play in an environment formalized as a set of *Z* states $S=\{S_{1},...,S_{Z}\}$. In each state, agent *i* can choose between *M* actions from the action set $𝒜=\{A_{1}^{i},...,A_{M}^{i}\}$. The probability that a transition from state *s* to state $\overset{´}{s}$ occurs when agents play the joint action a is $T^{s,a,\overset{´}{s}}$. The right panel represents reward discretization and Q-return distribution estimation for one agent *i*. 1) An environmental reward quantity, $\tilde{r}$, is detected with smoothing width σ by several neurons (in light blue). Each neuron *k* represents the probability that the environmental reward equals a quantity $r_{k}\pm \Delta r$; this probability is the area, taken between $r_{k}-\Delta r$ and $r_{k}+\Delta r$, under the Gaussian curve centered around $\tilde{r}$ with standard deviation σ. 2) This yields the discretized reward distribution $R^{i,s,a,\overset{´}{s},r}$. In DDRL, all quantities are no longer calculated as average estimates, but rather as discretized distributions. 3) Example Q-return distribution, which is calculated according to our DDRL dynamics (Section 3.1). 4) The optimistic heuristic is applied to the Q-return distribution (in blue). It transforms the original distribution into a distorted one (outlined in magenta). The mean of this new distribution (magenta bar) is an optimistic estimate of the average Q-value (black bar).

In the following subsections, we explain how we discretize and compute the relevant distributions.

#### 3.1.1. Discretization and reward distribution.

In contrast to standard distributional RL, we already discretize the rewards and not just the returns. We do so to be able to compute the return distribution in closed form (Sect. 3.1.4), which is necessary to formulate the deterministic dynamics. We assume an agent has n∈ℕ reward “neurons,” each neuron being sensitive to a given range of amount of reward. Throughout this work we use *n* = 29, balancing accuracy and computational cost across environments (see S1 Text, Part IV). We define $r_{1}\in R$ (*resp.* $r_{n}\in R$) the minimal (*resp.* maximal) amount of reward that can be detected. Similarly to categorical DistRL [46], we assume a regular grid $(r_{k}{)}_{k\in [1,n]}$ such that $r_{k}=r_{1}+(k-1)\Delta r$, where $\Delta r=\frac{r_{n}-r_{1}}{n-1}$ denotes the width separating two consecutive grid points.

Fig 1 describes the process of discretization by reward neurons. In particular, it shows how an arbitrary reward amount $\tilde{r}$ gets represented as a discretized distribution by defining a Gaussian kernel centered around $\tilde{r}$. To smooth out the effects of discretization, we assume that each neuron *k* is maximally sensitive to the reward amount $r_{k}$, quantified by a smoothing width, σ. Each reward neuron $r_{k}$ “fires” with intensity $P_{\sigma }(r_{k}|\tilde{r})$, which quantifies the probability that $r_{k}-\frac{\Delta r}{2}<\tilde{r}<r_{k}+\frac{\Delta r}{2}$. More precisely, $P_{\sigma }(r_{k}|\tilde{r})$ is calculated as the area between $r_{k}-\frac{\Delta r}{2}$ and $r_{k}+\frac{\Delta r}{2}$ under the Gaussian bell curve centered around $\tilde{r}$ and with standard deviation σ,


$$\begin{array}{c} P_{\sigma }(r_{k}|\tilde{r})=\int_{r_{k}-\frac{\Delta r}{2}}^{r_{k}+\frac{\Delta r}{2}}\frac{1}{\sqrt{2\pi \sigma^{2}}}\exp\left[-\frac{(\rho -\tilde{r}{)}^{2}}{2\sigma^{2}}\right]d\rho . \end{array}$$ (14)

Therefore, this smoothing procedure captures some uncertainty about the categorization into different bins. This uncertainty is modulated by the smoothing width, σ. This smoothing is a soft projection: each atom or neuron $r_{k}$ receives probability mass $P_{\sigma }(r_{k}|\tilde{r})$ rather than all mass going to the nearest neuron. It is analogous to label smoothing in supervised learning [50] and has been applied in distributional RL by Farebrother et al. [51]. Biologically, it reflects the overlapping tuning curves of reward-sensitive neurons, which respond to nearby stimuli [52,53]. Ultimately, this discretization method allows us to transform any stochastic game’s reward tensor into a reward distribution. For all $r\in \{r_{1},...,r_{n}\}$, we define the reward distribution $R^{i,s,a,\overset{´}{s},r}$ for agent *i*, current state *s*, joint action a, and next state $\overset{´}{s}$, as:


$$\begin{array}{c} R^{i,s,a,\overset{´}{s},r}=P_{\sigma }(r|R^{i,s,a,\overset{´}{s}}). \end{array}$$ (15)

Note that each $R^{i,s,a,\overset{´}{s}}$ is an entry of the original reward tensor R. In other terms, $R^{i,s,a,\overset{´}{s},r}$ is the area between $r-\frac{\Delta r}{2}$ and $r+\frac{\Delta r}{2}$ under the bell curve centered around $R^{i,s,a,\overset{´}{s}}$.

#### 3.1.2. Reward distributions $R_{𝐗}^{i,s,a^{i},r}$ and $R_{𝐗}^{i,s,r}$.

The previous reward distribution can be averaged over joint actions a and next states $\overset{´}{s}$. We call $R_{𝐗}^{i,s,a^{i},r}$ the reward distribution for agent *i* taking action $a^{i}$ in state *s*, averaged over transitions to the next state $\overset{´}{s}$, and over other agents’ actions $a^{-i}$:


$$\begin{array}{c} R_{𝐗}^{i,s,a^{i},r}=\sum_{\overset{´}{s}}\sum_{a^{-i}}\prod_{j\neq i}X^{j,s,a^{j}}T^{s,a,\overset{´}{s}}R^{i,s,a,\overset{´}{s},r}. \end{array}$$ (16)

The reward distribution $R_{𝐗}^{i,s,r}$ denotes the probability that agent *i* receives reward *r* in state *s*. It is averaged over joint actions a (including agent *i*’s) and next states $\overset{´}{s}$:


$$\begin{array}{c} R_{X}^{i,s,r}=\sum_{\overset{´}{s}}\sum_{a}\prod_{j}X^{j,s,a^{j}}\cdot T^{s,a,\overset{´}{s}}\cdot R^{i,s,a,\overset{´}{s},r}. \end{array}$$ (17)

#### 3.1.3. Bellman equation and smoothed Bellman Kronecker $\delta^{i,v,r,\overset{´}{v}}$.

In traditional, expected-value RL, the value of a state *s* for agent *i* is computed via the Bellman equation:


$$\begin{array}{c} V_{X}^{i,s}=(1-\gamma^{i})R_{X}^{i,s}+\gamma^{i}\sum_{\overset{´}{s}}T_{𝐗}^{s,\overset{´}{s}}V_{X}^{i,\overset{´}{s}} \end{array}$$

where $V_{X}^{i,\overset{´}{s}}$ is the value of the next state, $\overset{´}{s}$, and $T_{𝐗}^{s,\overset{´}{s}}$ is the effective state transition matrix given joint policy X.

In distributional form, we want to compute the probability that (discretized) values *r*, *v* and $\overset{´}{v}\in \{r_{1},...,r_{n}\}$ satisfy the Bellman equation $v=(1-\gamma^{i})r+\gamma^{i}\overset{´}{v}$. To this end, we introduce a smoothed Bellman Kronecker delta,


$$\begin{array}{c} \delta^{i,v,r,\overset{´}{v}}=P_{\sigma }(v|(1-\gamma^{i})r+\gamma^{i}\overset{´}{v}), \end{array}$$ (18)

using the same discretization method as above. The idea is that $\delta^{i,v,r,\overset{´}{v}}$ peaks when the Bellman condition $v=(1-\gamma^{i})r+\gamma^{i}\overset{´}{v}$ is fulfilled for agent *i*, and its reward bins *v*, *r*, and $\overset{´}{v}$, allowing for some uncertainty, modulated by the smoothing width, σ, around the discretization into different bins. We use this smoothed Bellman Kronecker delta to compute the V-return distribution $V_{X}^{i,s,v}$ in closed form.

#### 3.1.4. V-return distribution $V_{𝐗}^{i,s,v}$.

The V-return distribution $V_{𝐗}^{i,s,v}$ represents the probability that agent *i* in state *s* receives a long-term return *v*. It results from four instances that must occur together. For each short-term reward bin r∈R, for all possible next states $\overset{´}{s}\in S$, and each long-term return bin $\overset{´}{v}\in R$, i) *v*, *r* and $\overset{´}{v}$ must fulfill the Bellman equation according to probability $\delta^{i,v,r,\overset{´}{v}}$, ii) agent *i* must receive short-term reward *r* in state *s*, according to probability $R_{X}^{i,s,r}$, iii) state *s* must transition to state $\overset{´}{s}$, according to probability $T_{X}^{s,\overset{´}{s}}$, and finally iv) in state $\overset{´}{s}$, agent *i* must receive a long-term return $\overset{´}{v}$. More concisely,


$$\begin{array}{c} V_{X}^{i,s,v}=\sum_{r,\overset{´}{v},\overset{´}{s}}\delta^{i,v,r,\overset{´}{v}}R_{X}^{i,s,r}T_{X}^{s,\overset{´}{s}}V_{X}^{i,\overset{´}{s},\overset{´}{v}}. \end{array}$$ (19)

We now summarize the smoothed Bellman-Kronecker delta, reward, and transition probabilities into a tensor $M_{X}^{i,s,v,\overset{´}{s},\overset{´}{v}}=\sum_{r}\delta^{i,v,r,\overset{´}{v}}R_{X}^{i,s,r}T_{X}^{s,\overset{´}{s}}.$ Doing so, we can rewrite Eq. 19 as $V_{X}^{i,s,v}=\sum_{\overset{´}{v},\overset{´}{s}}M_{X}^{i,s,v,\overset{´}{s},\overset{´}{v}}V_{X}^{i,\overset{´}{s},\overset{´}{v}}$. We now combine the indices s×v=w and $\overset{´}{s}\times \overset{´}{v}=\overset{´}{w}$, which transforms Eq. 19 into,


$$\begin{array}{c} V_{X}^{i,w}=\sum_{\overset{´}{w}}M_{X}^{i,w,\overset{´}{w}}V_{X}^{i,\overset{´}{w}} \end{array}$$ (20)

In other terms, for each agent *i* and joint policy *X*, the V-return distribution $V_{X}^{i}$ fulfills $V_{X}^{i}=M_{X}V_{X}^{i}$. Thus, $V_{X}^{i}$ is the eigenvector of the matrix $M_{X}$ corresponding to eigenvalue 1, normalized in such a way that for each state *s*, the probabilities to receive values v∈R sum up to 1, $\sum_{v}V_{X}^{i,s,v}=1$.

#### 3.1.5. Q-return distribution $Q_{X}^{i,s,a^{i},v}$.

The probability for agent *i* to receive long-term return *v* in state *s* under action $a^{i}$ is the joint probability that from all possible short-term reward values *r*, all possible next states $\overset{´}{s}$, and all possible next long-term returns $\overset{´}{v}$, i) *v*, *r* and $\overset{´}{v}$ fulfill the Bellman equation according to probability $\delta^{i,v,r,\overset{´}{v}}$, ii) agent *i* receives short-term reward *r* in state *s* under action $a^{i}$, according to probability $R_{X}^{i,s,a^{i},r}$, iii) state *s* transitions to state $\overset{´}{s}$ under agent *i*’s action $a^{i}$, according to probability $T_{X}^{i,s,a^{i},\overset{´}{s}}$ (the effective transition given joint strategy X, marginalized over the other agents’ actions), and iv) in state $\overset{´}{s}$, agent *i* receives a long-term return $\overset{´}{v}$, according to probability $V_{X}^{i,\overset{´}{s},\overset{´}{v}}$. More concisely,


$$\begin{array}{c} Q_{X}^{i,s,a^{i},v}=\sum_{\overset{´}{s},r,\overset{´}{v}}\delta^{i,v,r,\overset{´}{v}}\cdot R_{X}^{i,s,a^{i},r}\cdot T_{X}^{i,s,a^{i},\overset{´}{s}}\cdot V_{X}^{i,\overset{´}{s},\overset{´}{v}} \end{array}$$ (21)

#### 3.1.6. Temporal-difference error.

The SARSA and Actor-Critic temporal-difference errors differ slightly (see Section 2.4). Denoting $\langle .\rangle_{d}$ the mean of a distribution on dimension *d*, the deterministic approximation of the temporal-difference error at time *t* for a SARSA agent *i* taking action $a^{i}$ in s*t*ate *s*, $E_{{𝐗}_{t}}^{i,s,a^{i}}$, is:


$$\begin{array}{c} E_{{𝐗}_{t}}^{i,s,a^{i}}=\langle Q_{{𝐗}_{t}}^{i,s,a^{i},v}\rangle_{v}-\frac{1}{\beta^{i}}\cdot \log X_{t}^{i,s,a^{i}}. \end{array}$$ (22)

For an Actor-Critic agent, the last term is not needed, as the “critic” (*i.e.*, $\langle V_{{𝐗}_{t}}^{i,s,v}\rangle_{v}$) is constant in action (see Section 2.4). The remaining $\langle Q_{{𝐗}_{t}}^{i,s,a^{i},v}\rangle_{v}$ in Eq. 23 is therefore not the full TD error in the classical sense; it is the action-dependent component, which yields the same deterministic strategy update as the full TD error because the omitted critic is constant across actions and cancels in the Boltzmann update. Therefore, the deterministic approximation of the temporal-difference error at time *t* for an Actor-Critic agent *i* taking action $a^{i}$ in state *s*, $E_{{𝐗}_{t}}^{i,s,a^{i}}$, is:


$$\begin{array}{c} E_{{𝐗}_{t}}^{i,s,a^{i}}=\langle Q_{{𝐗}_{t}}^{i,s,a^{i},v}\rangle_{v}. \end{array}$$ (23)

#### 3.1.7. Biasing the Q-return distribution.

Optimism (*resp.* pessimism) causes agents to overestimate (*resp.* underestimate) Q-values. There are different ways to obtain an optimistic (*resp.* pessimistic) estimate of a distribution. Here, we adopt a weights method: return distribution mass is rescaled by $w_{U}$ above the threshold $V_{𝐗}^{i,s}$ and by unity below (reversed for pessimism). The primary motivation is a formal equivalence with asymmetric RL at a two-armed bandit choice task: a weights-DDRL agent with upper weight $w_{U}$ reaches the same steady-state Q-values as an asymmetric-RL agent with bias ratio $b=\alpha^{+}/\alpha^{-}=w_{U}$, where $\alpha^{+}$ and $\alpha^{-}$ are the learning rates applied to positive and negative temporal-difference errors, respectively, (proven in S1 Text, Section 12.2), directly connecting this probability distortion to the established cognitive science literature [54]. In S1 Text, Section 12.1 we compare this against quantile- and expectile-based distortions of the return distribution [29]. While the qualitative outcomes are similar, these other distortions exhibit a less smooth parameter dependence as a result of our categorical discretization. In contrast, our weights method yields smooth, monotonic dependence on $w_{U}$.

The weights method involves applying a weights tensor, $w^{i,s,a^{i},v}$, to the discretized distribution $Q_{𝐗}^{i,s,a^{i},v}$ so as to distort it. Although there are different ways to distort a distribution, we use a stepwise function for simplicity, and because it is equivalent with asymmetric RL on a two-armed bandit task (see S1 Text, Section 12.2). For agent *i* in state *s*, the step is located at the scalar $V_{𝐗}^{i,s}$ defined below: the mean of the action-averaged return distribution. When the agent is optimistic (*resp.* pessimistic), a higher weight is put on the part of the distribution which is above (*resp.* below) that step.

We define the threshold $V_{𝐗}^{i,s}$ as the expected value of the action-averaged return distribution at state *s* under joint strategy X. Marginalizing $Q_{X}^{i,s,a^{i},v}$ over agent *i*’s strategy yields the state-value distribution $V_{𝐗}^{i,s,v}=\sum_{a^{i}}X^{i,s,a^{i}}Q_{𝐗}^{i,s,a^{i},v}$ (consistent with Eq. 19); taking its mean over *v* gives the scalar threshold,


$$\begin{array}{c} V_{𝐗}^{i,s}\;=\;\langle V_{𝐗}^{i,s,v}\rangle_{v}\;=\;\sum_{v\in ℛ}v\sum_{a^{i}\in {𝒜}^{i}}X^{i,s,a^{i}}\;Q_{𝐗}^{i,s,a^{i},v}. \end{array}$$ (24)

We define the weights tensor $w^{i,s,a^{i},r_{k}},r_{k}\in R,$ as follows. For all agent *i*, state *s*, action $a^{i}$, and neuron $r_{k}$,

- $w^{i,s,a^{i},r_{k}}=1$ if $r_{k}<V_{𝐗}^{i,s}$;
- $w^{i,s,a^{i},r_{k}}=w_{U}$ if $r_{k}\geq V_{𝐗}^{i,s}$,

where $w_{U}<1$ if the agent is pessimistic, and $w_{U}>1$ if it is optimistic.

We obtain the weighted estimate $\langle Q^{i,s,a^{i},v}\rangle_{w}$ of distribution $Q_{𝐗}^{i,s,a^{i},v}$ by computing the product of the distribution with the weights vector, normalizing this product by a normalization factor $N^{i,s,a^{i}}$, and computing the mean $\langle Q_{𝐗}^{i,s,a^{i},v}\rangle_{w}$ of the resulting distribution:


$$\begin{array}{c} \langle Q_{𝐗}^{i,s,a^{i},v}\rangle_{w}=\frac{1}{N^{i,s,a^{i}}}\sum_{r_{k}\in R}r_{k}\cdot w^{i,s,a^{i},r_{k}}\cdot Q_{𝐗}^{i,s,a^{i},r_{k}} \end{array}$$ (25)

The normalization factor, $N^{i,s,a^{i}}=\sum_{r_{k}\in R}w^{i,s,a^{i},r_{k}}\cdot Q_{𝐗}^{i,s,a^{i},r_{k}}$, helps us make sure the integral of the weighted distribution over R is 1.

To validate our framework and highlight its usefulness for uncovering new insights, we test our model across three different choice environments: an individual bandit choice task under resource variability, a social coordination challenge, and an intertemporal risky choice problem.

### 3.2. Example application: Bandit choice under resource variability

Here, we use our model to confirm previous findings showing that the influence of optimism on performance in a two-armed bandit task is modulated by resource scarcity. The two-armed bandit is a standard task in cognitive psychology to study asymptotic strategy formation between options with different reward probabilities. An agent must choose between two options, *a*_1_ and *a*_2_. *a*_1_ gives a reward +1 with probability *p*_1_, and a punishment −1 with probability $1-p_{1}$; *a*_2_ gives a reward +1 with probability *p*_2_, and a punishment −1 with probability $1-p_{2}$. By repeatedly choosing between the two options, the agent must learn which is most rewarding on average (Fig 2A). We use SARSA rather than Actor-Critic dynamics here because Actor-Critic dynamics (analogous to the pure replicator equation), drive agents toward deterministic strategies, whereas SARSA (analogous to the replicator-mutator equation) admits mixed asymptotic strategies, as observed in previous research on this task [54].

**Fig 2:**
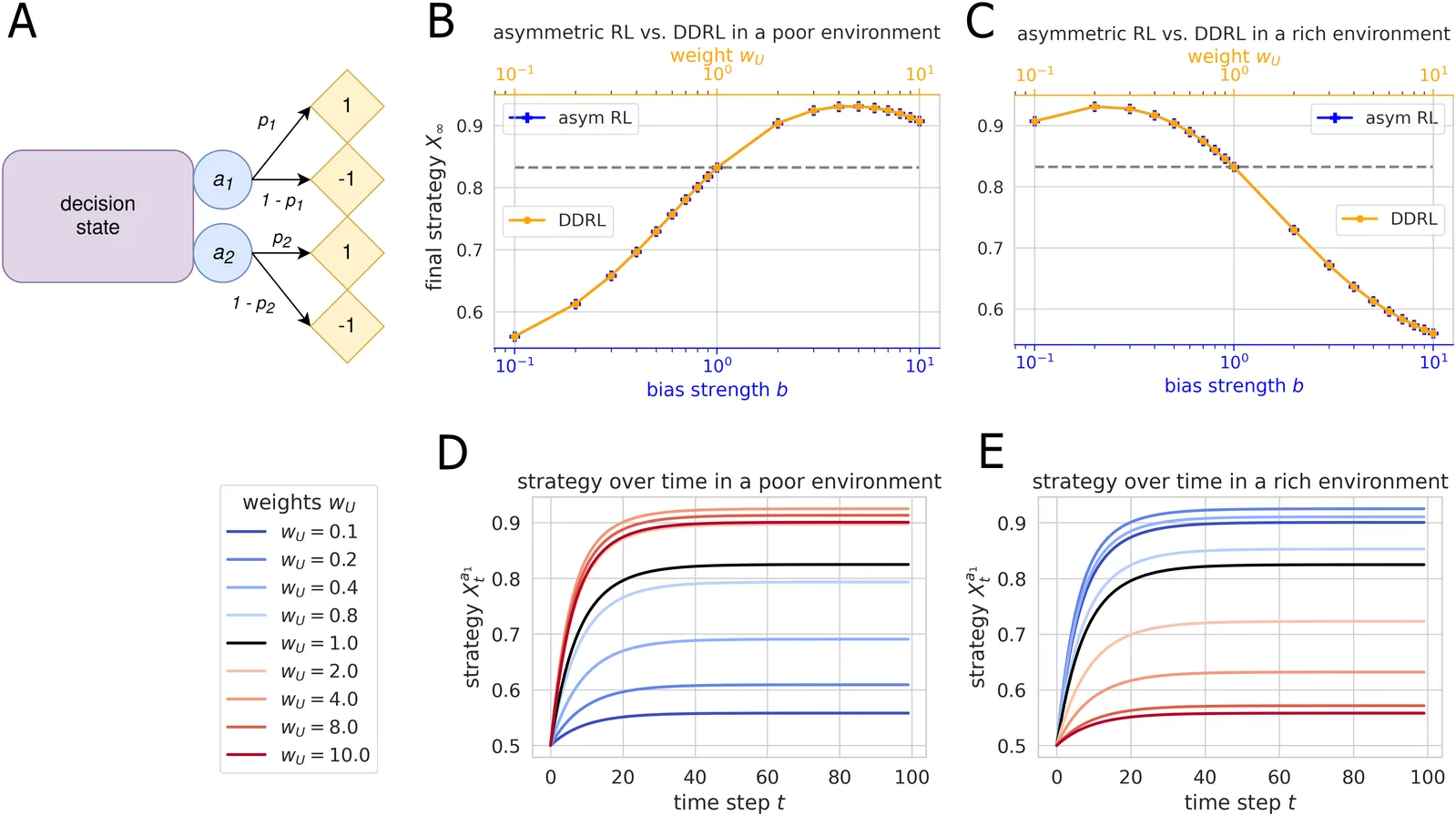
Influence of optimism/pessimism on a two-armed bandit environment with varying resource availability. **A** The two-armed bandit environment. **B**–**C** Comparison between final strategies $X_{\infty }$ obtained *via* DDRL (yellow curve) vs. asymmetric RL (blue curve), **B** on a “poor” two-armed bandit task (*p*_1_ = 0.3, *p*_2_ = 0.1); **C** on a “rich” two-armed bandit task (*p*_1_ = 0.9, *p*_2_ = 0.7). These final strategies vary as a function of bias strength $b=\frac{\alpha^{+}}{\alpha^{-}}$. For DDRL agents, the final strategies were obtained from a trajectory of 150 time steps, after checking that this number of time steps was sufficient for convergence (see Fig 2D and 2E). For asymmetric agents, the final strategies were obtained analytically, using the formula in [54]. **D**–**E** Strategy plots of a DDRL agent performing a two-armed bandit task with various levels of optimism/pessimism, **D** in a poor environment (*p*_1_ = 0.3, *p*_2_ = 0.1); **E** in a rich environment (*p*_1_ = 0.9, *p*_2_ = 0.7). These plots represent the evolution of an agent’s probability to choose the best arm over time. Blue lines indicate pessimistic agents ($w_{U}<1$), while red lines indicate optimistic agents ($w_{U}>1$). Neutral agents ($w_{U}=1$) are represented by a black line. In all panels, all DDRL agents had *n* = 29 neurons, and a smoothing width σ=0.0001 to minimize errors (see S1 Text, Part **IV)**.

In previous research using two-armed bandit tasks, it has been established via computational modeling that optimism (implemented as an asymmetric pair of learning rates) increases or decreases an agent’s performance depending on resource scarcity. Optimism is beneficial for performance in scarce environments, and detrimental in abundant environments [54]. Empirical research on human decision-making also revealed an asymmetric sensitivity to positive and negative prediction errors. Individual differences in the ratio of positive to negative learning rates predict risk-seeking behavior in bandit tasks [55] and reflect variation in dopaminergic signaling [56,57]. Humans tend toward a stable positivity bias across environments rather than adaptively modulating this asymmetry with resource scarcity [58,59], consistent with the non-adaptive optimism weights in DDRL. Therefore, we tested our framework on a “poor” two-armed bandit, in which the arms’ reward probabilities are *p*_1_ = 0.3 and *p*_2_ = 0.1, and on a “rich” version of the same task, in which the arms’ reward probabilities are *p*_1_ = 0.9 and *p*_2_ = 0.7.

First, we find that DDRL closely approximates the behavior of an asymmetric-RL agent on a two-armed bandit task (Fig 2B and 2C). To do so, we checked that both algorithms converged towards the same final strategy $X_{\infty }$–i.e., an agent’s probability to choose the best arm after convergence. We computed final strategies for different bias strengths. In asymmetric RL, bias strength is defined as $b=\frac{\alpha^{+}}{\alpha^{-}}$ (where $\alpha^{+}$ updates positive temporal-difference errors and $\alpha^{-}$, negative temporal-difference errors). An analytical calculation of $X_{\infty }$ as a function of *b* is given in [54]. In DDRL, bias strength is modulated by the upper weight, $w_{U}$. Here, we simulated the deterministic trajectory of an agent’s strategy *X* over time until convergence for different values of $w_{U}$.

Second, we replicate the aforementioned findings on the influence of optimism/ pessimism in poor/rich environments (Fig 2B and 2C). In both scarcity conditions, DDRL closely approximates asymmetric RL. More precisely, the final strategy $X_{\infty }$ of a DDRL agent with weight $w_{U}$ matches the final strategy of an asymmetric-RL agent with bias strength $b=w_{U}$. We give a formal proof of this equivalence in S1 Text, Section 12.2. Fig 2B and 2C shows how the influence of optimism/pessimism on performance is modulated by resource scarcity. In a poor environment (Fig 2B), optimistic agents ($w_{U}>1$) converge to a higher probability of choosing the best arm than neutral ($w_{U}=1$) and pessimistic ($w_{U}<1$) agents. In a rich environment (Fig 2C), by contrast, pessimistic agents converge to a higher probability of choosing the best arm than neutral ($w_{U}=1$) and optimistic ($w_{U}>1$) agents. Furthermore, both curves indicate the presence of an optimal bias strength (or upper-weight value), for which the final strategy $X_{\infty }$ is maximal. In our poor environment, this optimal upper-weight value lies close to $w_{U}=5$; whereas, in our rich environment, it is close to $w_{U}=0.2$.

This interaction between optimism/pessimism and resource scarcity, previously established in Ref [54], is further visualized in Fig 2D and 2E. These strategy plots depict the evolution of a DDRL agent’s strategy–i.e., its probability of choosing the best arm–over time for different levels of optimism/pessimism. In a poor environment (Fig 2D), starting from a uniform random strategy (*X* = 0.5), optimistic DDRL agents ($w_{U}>1$, red lines) converge towards a higher probability of choosing the best arm than the neutral agent ($w_{U}=1$, black line); whereas pessimistic DDRL agents ($w_{U}<1$, blue lines) converge towards lower performance. In line with previous findings [54] and the results mentioned above, this pattern is reversed in a rich environment (Fig 2E).

Overall, our results confirm that DDRL closely approximates asymmetric RL on a two-armed bandit task with varying resource scarcity. In particular, we replicate previous findings indicating that optimism is beneficial in poor environments, while pessimism enhances performance on the rich version of the task. This means DDRL is a suitable model for optimism/pessimism in this basic choice task. Next, we turn to an environment which is widely used in biology, economics, and sustainability science: the Stag-Hunt game.

### 3.3. Example application: Social coordination task

Here, we use our model to confirm previous findings that show optimism improves coordination in the Stag-Hunt game. The Stag-Hunt game is an iconic coordination problem in the space of social dilemmas [60]. A social dilemma is a situation in which agents individually have an incentive to deviate from outcomes that would be best for the group. Each agent must choose between two actions: cooperate (*c*) or defect (*d*). In the two-agent case, if both cooperate, they get a payoff *R* (called “reward”). If both defect, they get a payoff *P* (called “punishment”). If one cooperates and the other defects, the cooperator receives a payoff of *S* (called “sucker”) and the defector gets a payoff of *T* (called “temptation”) (Fig 3A). In the Stag-Hunt game, the payoff ordering is as follows: *R* > *T* > *P* > *S*. Therefore, both mutual defection and mutual cooperation are Nash equilibria. However, if both agents defect, they receive the punishment payoff *P*, which is lower than the maximum reward payoff *R* obtainable by mutual cooperation. This means individual interests align with collective interests, but the best outcome can only be achieved through coordination among agents.

**Fig 3:**
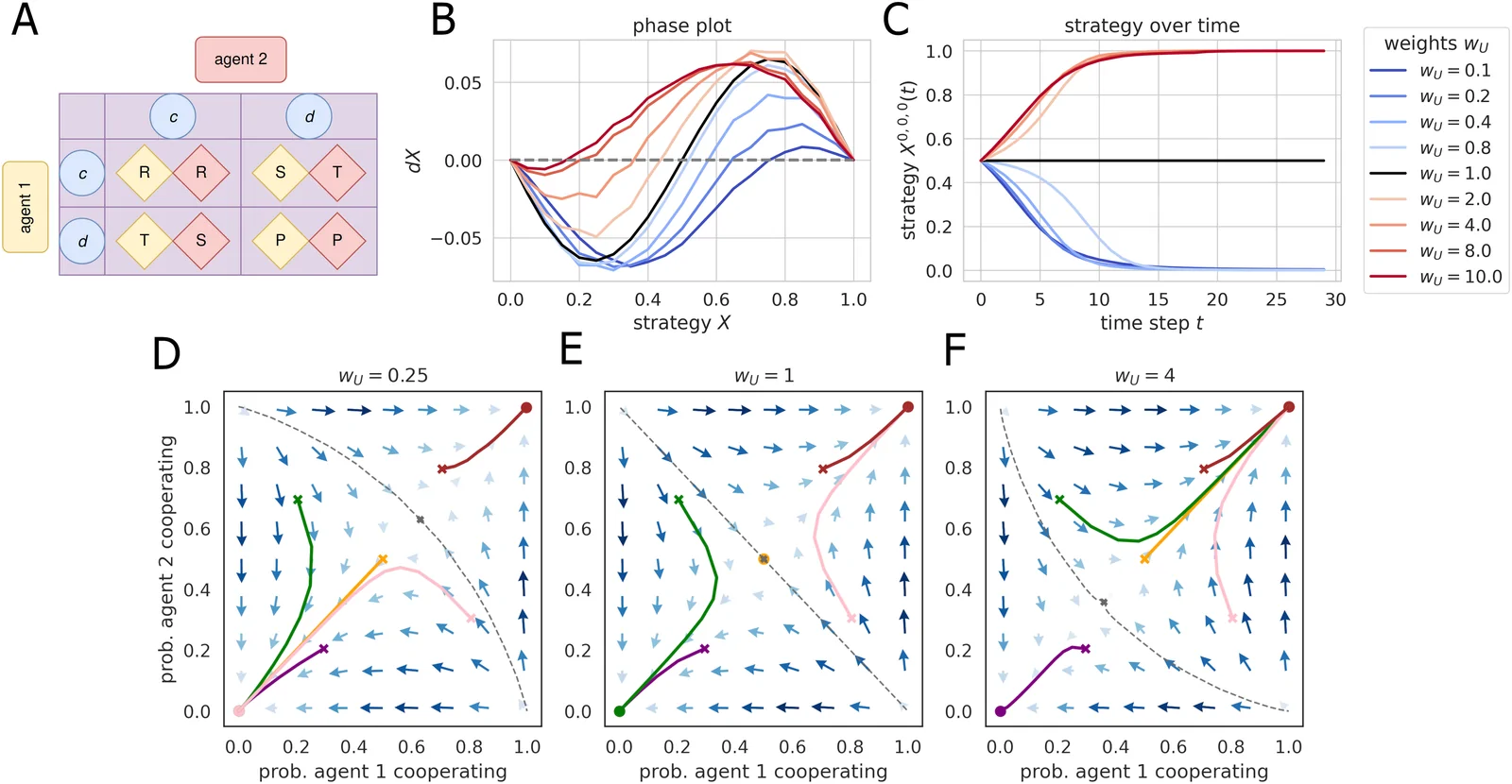
Influence of optimism/pessimism on a Stag-Hunt game. **A** Structure of a social dilemma. In the Stag-Hunt game, *R* > *T* > *P* > ***S*.** For our analyses, we set *R* = 3, *T* = 1, *P* = 0 and S=−2 as illustrative values satisfying this ordering; the qualitative structure of fixed points and their stability depends only on the ordering, not the specific magnitudes. **B** Phase plot and **C** strategy plot of an agent playing the Stag-Hunt game for various levels of optimism/pessimism. Since the Stag-Hunt game is symmetric among agents, we assumed the second agent had the same strategy as the first. On the phase plot **(B)**, *X* = 1 indicates maximal cooperation, and *X* = 0, maximal defection. Intersections of the curves with the *x*-axis indicate stable and unstable fixed points, depending on the sign of the derivative *dX*. The strategy plot (**C**) represents the evolution of an agent’s probability to cooperate over time, given an initial cooperation probability of 0.5. Blue lines represent pessimistic agents ($w_{U}<1$), while red lines represent optimistic agents ($w_{U}>1$). Neutral agents ($w_{U}=1$) are represented by a black line. **D**–**F** Flow plots of two agents playing the Stag-Hunt game **D** with a pessimistic heuristic ($w_{U}=0.25$); **E** with no heuristic (“neutral,” $w_{U}=1$); **F** with an optimistic heuristic ($w_{U}=4$). Arrows represent the direction and intensity of the strategies’ derivative *dX*. Colored lines are sample trajectories starting from various initial conditions (+), while round dots indicate fixed points. Dashed gray lines delineate the flow plots’ separatrices–i.e., the threshold of initial cooperation probability above which agents learn towards maximal cooperation, and below which they learn towards maximal defection. In all panels, all DDRL agents had *n* = 29 neurons, and a smoothing width σ=0.08 to minimize errors (see S1 Text, Part **IV)**.

Previous research in multi-agent reinforcement learning suggests that variants of optimism enhance coordination on various tasks. For instance, Lenient Learning, an algorithm that initially has agents dismiss negative outcomes, has led to performance improvements across multiple coordination games, such as the climb and penalty games [23]. Likewise, Hysteretic Q-learning (a multi-agent equivalent of asymmetric RL) has been shown to improve coordination on several tasks: the aforementioned climb and penalty games, a collaborative ball balancing task, and a pursuit game (where predators need to coordinate to capture prey) [22]. In this section, we investigate whether optimism enhances coordination in the Stag-Hunt game. The climb and penalty games are two three-action versions of the Stag-Hunt problem, which received its name from a story in which hunters need to coordinate to capture a stag, analogous to predators coordinating to capture prey. Thus, the Stag-Hunt game is well-suited to capture the essence of these coordination problems. Human experimental evidence corroborates this connection. When participants reason under time pressure, favoring intuitive, optimistic social expectations, they cooperate more in Stag-Hunt tasks than when reasoning deliberatively [61]. Shared optimistic beliefs about a partner’s trustworthiness increase cooperation by up to 40 percentage points [62], and optimism about partner behavior predicts payoff-dominant choices in experimental coordination tasks [63].

In line with previous findings, we find that optimistic DDRL agents converge to mutual cooperation when starting from a uniformly random strategy. In contrast, pessimistic agents converge to the suboptimal equilibrium of mutual defection. A neutral agent remains at its mixed strategy for the payoff values of our Stag-Hunt game (Fig 3C). Fig 3B shows that the uniform random strategy (i.e., a cooperation and defection probability of 0.5) is an unstable fixed point of the learning dynamics for neutral agents. Therefore, if a neutral agent’s initial strategy is higher than 0.5 (and provided the second agent’s initial strategy is the same), it converges toward cooperation. If, by contrast, both agents’ initial strategies are below 0.5, they converge toward defection. Optimism (red lines in Fig 3B) lowers this threshold–i.e., two optimistic DDRL agents may converge towards cooperation even if they started with a strategy *X* < 0.5–while pessimism heightens it. This is why, in Fig 3C, when all agents start with a uniformly random strategy, optimistic agents end up mutually cooperating, whereas pessimistic agents converge towards mutual defection. Thus, optimism facilitates cooperation, while pessimism hinders it.

The flow plots in Fig 3D, 3E and 3F give an overview of the learning dynamics in the two-dimensional phase space spanned by each agent’s individual strategy. Thus, the phase plots in Fig 3B are equivalent to the diagonal in the flow plots in Fig 3D–3F, for the relevant optimism levels. The strategy changes *dX* are represented by the flow arrows. The separatrix curves separating the two basins of attraction of the two equilibria, mutual cooperation and mutual defection, are shown with dashed gray lines. Optimism distorts these separatrices. When agents are pessimistic, it takes higher initial levels of cooperation to eventually learn to fully cooperate (Fig 3D). When they are optimistic, learning to cooperate fully is possible even from initially lower levels of cooperation (Fig 3F).

Additionally, we tested our model on a prisoner’s dilemma with *T* > *R* > *P* > *S*. Here, agents have the incentive to defect, regardless of what the other agent does. Thus, mutual cooperation is no longer an equilibrium despite being the optimal solution for the collective. We find that optimism/pessimism does not affect the learning dynamics. In other terms, whatever the upper-weight value $w_{U}$, DDRL agents always converge towards mutual defection in this environment (see S1 Text, Section 13.3). In this dilemma, indeed, the reward *R* is lower than the temptation *T*. Therefore, if an agent is optimistic that the other will cooperate, it is still incentivized to defect, since it will then obtain *T* instead of *R*. The mutual-defection equilibrium thus cannot be changed through the optimism/pessimism heuristic. In a Stag-Hunt game, by contrast, if an agent is optimistic that the other agent will cooperate, it increases its incentive to cooperate as well (see S1 Text, Section 13.4 for an analytical explanation of this difference between the two games).

In short, our results confirm that optimism enhances coordination in DDRL agents, while pessimism hinders it. These results echo previous findings across several multi-agent reinforcement learning algorithms in coordination games. Additionally, our flow plot visualization provides further insights into the collective learning dynamics in coordination games and into how the optimistic/pessimistic heuristic distorts this landscape. Next, we consider a lesser-known intertemporal risky choice problem.

### 3.4. Example application: Intertemporal risky choice problem

Here, we use our model to confirm previous findings that show optimism increases risk-taking. We also uncover new findings in this lesser-known choice environment [39,64], as it incorporates a novel component of decision-making into our analysis: intertemporal choice. In the choice environment, an agent must trade off a low but safe reward with a high reward with potentially negative future consequences. This is implemented via two environmental states: a prosperous and a degraded one. The agent must choose between a risky action and a safe action. In the prosperous state, taking the risky action leads to a reward $r_{r}=1$, whereas the safe action leads to a reward $r_{s}=0.5$. However, choosing the risky action in the prosperous state results in a transition to the degraded state with probability $p_{c}=0.2$. There, the agent gets no reward $r_{d}=0$, and can only transition back to the prosperous state with probability $p_{r}=0.1$ if it chooses the safe action (Fig 4A). To simplify the analysis, we use an Actor-Critic agent here, since it has no explicit exploration term (see Section 2.2). We presume that a SARSA agent would exhibit additional, exploration-related effects and interactions.

**Fig 4:**
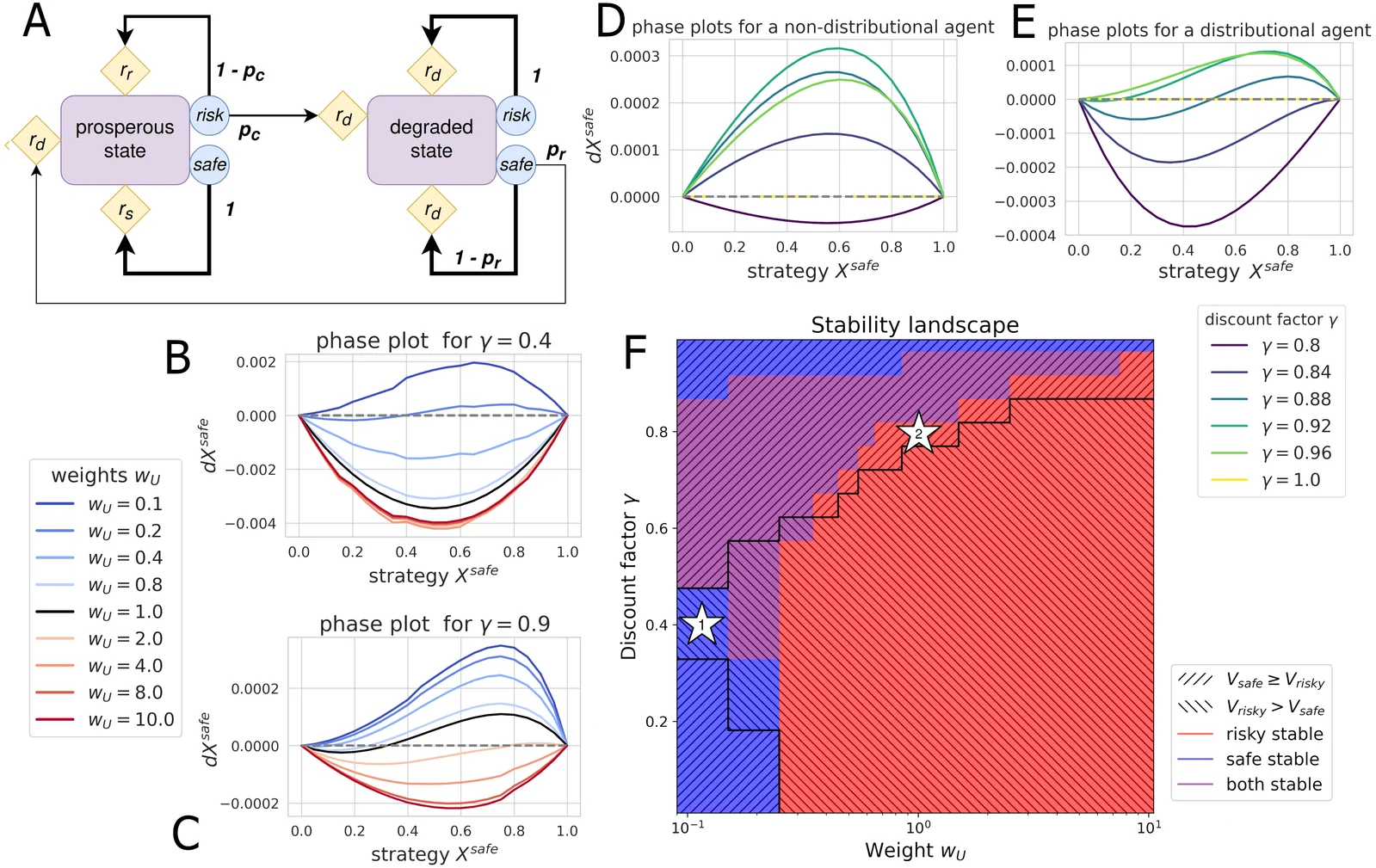
Influence of optimism/pessimism in a risk-reward dilemma. **A** Structure of a risk-reward dilemma. In our analyses, we set the collapse probability $p_{c}=0.2$ and the recovery probability $p_{r}=0.1$; we fixed the risky reward $r_{r}=1$, the safe reward $r_{s}=0.5$, and the degraded reward $r_{d}=0$. **B**–**C** Phase plots of a DDRL agent performing a risk-reward dilemma with various levels of optimism/pessimism, **B** with a low discount factor (γ=0.4); **C** with a high discount factor (γ=0.9). Blue lines indicate pessimistic agents ($w_{U}<1$), while red lines indicate optimistic agents ($w_{U}>1$). Neutral agents ($w_{U}=1$) are represented by a black line. **D**–**E** Phase plots of an agent performing a risk-reward dilemma with various discount factors, **D** using a regular deterministic Actor-Critic algorithm, **E** using a neutral ($w_{U}=1$) DDRL Actor-Critic algorithm. On all phase plots **(B–E)**, *X* = 1 corresponds to maximal safety, and *X* = 0, to maximal risk. Intersections with the *x*-axis indicate stable and unstable fixed points (depending on the sign of the derivative *dX*). **F** Stability landscape of a DDRL on a risk-reward dilemma for various combinations of weights $w_{U}$ and discount factors γ. Red patches correspond to regions in the parameter space where the risky policy ($X^{\text{prosp, risky}}=1$) is stable, while blue patches indicate that the safe policy ($X^{\text{prosp, safe}}=1$) is stable. Purple patches correspond to regions in the parameter space where both policies are stable. Stars mark regions of interest, where the safe or risky policy has highest value but is unstable. In all panels, the agent had *n* = 29 neurons, and a smoothing width σ=0.05 to minimize errors (see S1 Text, Part **IV)**.

To our knowledge, no behavioral experiment has studied an environment combining these features: repeated stochastic choice with state-dependent consequences and temporal recovery dynamics. Risk-taking and intertemporal discounting are each extensively studied in isolation; their combination has been explored in simplified choice tasks [65], but not in a multi-state repeated structure of this form. Previous research on risk-sensitivity in RL agents uses asymmetric learning rates to implement different attitudes towards risk, where optimism corresponds to risk-seeking and pessimism to risk-aversion [66,67]. Thus, we hypothesize that in our intertemporal risky choice problem, an optimistic DDRL agent is more likely to choose the risky action than a neutral agent. We also hypothesize that this pattern is reversed for pessimistic DDRL agents.

We confirm both hypotheses. Optimism increases risky behavior while pessimism favors safe behavior (Fig 4B and 4C). Fig 4B and 4C show the strategy change $dX^{\text{prosp, safe}}$ versus the strategy $X^{\text{prosp, safe}}$ to choose the safe action in the prosperous environmental state for different levels of optimism and pessimism and two discount factors. In the degraded state, only the safe action leads to a recovery to the rewarding prosperous state. Thus, we assumed $X^{\text{deg},\text{safe}}=1$ for Fig 4B and 4C. Flow plots of the entire strategy space for given discount factors and weight values can be found in S1 Text, Fig 14.6 and S1 Text, Fig 14.7.

When the agent cares little about future rewards (low discount factor γ=0.4, Fig 4B), only intense pessimism can push an agent to act safely. Only the most pessimistic agents ($w_{U}=0.1$ and $w_{U}=0.2$) have a chance to converge towards complete safety in the prosperous state ($X^{\text{prosp},\text{safe}}=1$). All the other agents, including the moderately pessimistic and neutral ones, converge towards maximal risk-taking ($X^{\text{prosp},\text{safe}}=0$). This means that, in such situations, to act safely, intense pessimism can compensate for an agent’s lack of concern for future rewards.

When the agent cares more about future rewards (high discount factor γ=0.9, Fig 4C), the more optimistic an agent is, the more likely it is to converge toward the risky strategy. In fact, for a moderately-to-strongly optimistic agent ($w_{U}\geq 4$), the risky strategy is the only stable one. A mildly optimistic agent ($w_{U}=2$) could converge to a maximally safe approach, but only if its initial safety levels are very high. A reversed pattern is found for the different levels of pessimism. Because γ is high, risk-seeking is overall lower than with a low discount factor.

In both cases, we observe instances of bistability in the strategy space. Which strategy the agent converges to depends on the initial strategy. This is particularly interesting as non-distributional agents (using expected values from a continuous reward spectrum) do not exhibit any bistable regimes (Fig 4D). This observation aligns with the finding that an optimal strategy in a Markov Decision Process is always deterministic [68]. Fig 4E compares our distributional agent dynamics without any bias ($w_{U}$ = 1) for different discount factors. We find certain discount factors γ (e.g., γ=0.88, γ=0.92) at which the learning dynamics are bistable. The neutral weight $w_{U}=1$ rules out optimism or pessimism as a cause; the only remaining structural difference between Fig 4D and 4E is therefore the discretization of the return distribution. Thus, bistability does not solely emerge from optimism or pessimism, but from an intrinsic feature of our DDRL framework: the discretization of the return distribution. Fig 4F visualizes how the stability of the safe and risky strategies depends on the interaction of bias weight values $w_{U}$ and discount factors γ. The red zone denotes the stability of the risky strategy ($X^{\text{prosp, risky}}=1$); the blue zone, that of the safe one ($X^{\text{prosp, safe}}=1$); whereas, in the purple zone, both strategies are stable. Thus, bistability emerges for a broad range of weights and discount factors, including for neutral agents ($w_{U}=1$). Overall, we find that these bistable regimes depend on the interplay among the discount factor, the optimism weight, and return discretization, whose granularity is set by the smoothing width σ.

We also find a kind of incoherent choice pattern, which we coin “individual dilemma.” Just as in a social dilemma, an agent would be better off playing the strategy that yields the highest value, but it cannot realize this, as it is pulled towards the other, suboptimal strategy, which is stable (Stars in Fig 4F). To see this, we highlighted the region in parameter space where the safe policy yields a higher value to the agent than the risky policy ($V_{\text{safe}}>V_{\text{risky}}$) and likewise, $V_{\text{risky}}>V_{\text{safe}}$. We define $V_{\text{safe}}=\langle V_{X}^{\text{prosp}}\rangle_{w_{U}}{|}_{X^{\text{prosp, safe}}=1}$ and $V_{\text{risky}}=\langle V_{X}^{\text{prosp}}\rangle_{w_{U}}{|}_{X^{\text{prosp, risky}}=1}$. For most parameter values, when a strategy yields a higher overall value, it is also stable. Nevertheless, Fig 4F exhibits some deviations from this pattern. For example, at $w_{U}=0.1$ and γ=0.402, the safe strategy is stable while the risky strategy yields a higher value (Star 1 in Fig 4F). At $w_{U}=1$ and γ=0.794, the risky strategy is stable, while the safe strategy yields a higher value (Star 2 in Fig 4F). Although the state value of the safe strategy is higher than that of the risky one, the state-action value of the risky action is higher than that of the safe action. In S1 Text, Fig 14.10, we show the corresponding agent’s V-return and Q-return distributions.

Overall, our results confirm previous findings regarding the relationship between optimism, pessimism, and risky behavior. Optimism enhances risky behavior, whereas pessimism favors risk-averse behavior. Additionally, our framework generates two novel predictions: bistable regimes and individual dilemmas. Both have no counterpart in non-distributional dynamics and await empirical test.

## 4. Discussion

In this work, we derived the deterministic dynamics of distributional multi-agent reinforcement learning to obtain a unifying, biologically plausible, and tractable framework for modeling individual cognition and behavior across contexts. We applied our framework to model the widely studied optimism heuristic. We established the soundness of our framework by reproducing results across three iconic choice domains: a bandit choice task under resource variability, a social coordination challenge, and a risky choice problem. This demonstrates the potential of our model as a unifying framework to study the optimism-pessimism heuristic in collective learning dynamics.

Our framework rests on a simple, biologically plausible mechanism in which outcome distributions are discretized by a set of neurons. Empirical research has recently provided evidence for an analogous computation of distributions during learning and decision-making in the brain [28,69]. Thus, our return discretization should not be viewed simply as a technical trick, but as a meaningful biological feature. Distributional coding is empirically distinguishable from a population code for expected value. A code representing only the mean return would not generate the systematic diversity in prediction-error asymmetries observed in dopaminergic neurons: some neurons learn faster from better-than-expected outcomes, others from worse-than-expected outcomes, together spanning the full distribution of returns [28]. D1 and D2 striatal neurons encode opposite tails of the return distribution through an opponent circuit [70], and distributional coding has also been observed in prefrontal cortex [71]. These findings point to a specific neural substrate for distributional rather than mean-value coding.

In this regard, our framework could help enhance understanding of how reward information is encoded neurologically. How many neurons are involved in categorizing environmental rewards, and how does this number impact the accuracy of this distribution? In S1 Text, we show how the smoothing width, σ, affects the error between the expected values of the continuous and discretized reward spectra across various environments (see S1 Text, Part IV). In particular, we found that, for a fixed number of neurons *n*, the smoothing width σ that minimizes this error is environment-dependent. Thus, is there a context-dependent mechanism that modulates the smoothing width? Recent studies suggest such a context-dependent mechanism. It has been found that humans do not encode objective outcomes but rather evaluate options with respect to available alternatives [72]. This has been modeled with RL as range normalization [73], in which the value of an option is rescaled by the difference between its maximum and minimum values in the environment. Our framework could incorporate such a mechanism to explore how varying assumptions about neurological reward representations affect outcomes, leading to empirically testable hypotheses.

Furthermore, our framework could help uncover the empirical basis for optimistic reinforcement learning in the brain. In particular, it is an open question whether optimism in the brain manifests at the level of state-action values or temporal-difference errors. Indeed, asymmetric RL, which is widely used in psychological experiments, locates optimism/pessimism at the level of temporal-difference errors. These are updated differentially through asymmetric learning rates. By contrast, distributional reinforcement learning distorts the state-action return distribution by applying weights. According to prior work, these implementations should produce the same results in stateless environments, but they are expected to yield different outcomes in stateful environments [29]. Thus, comparing our framework with asymmetric RL algorithms in stateful choice environments could generate empirically testable hypotheses that would distinguish between signatures of state-action values versus temporal-difference-error optimism in the brain.

Applying our model to a single-agent intertemporal risky-choice task, we found that return discretization with Gaussian smoothing can lead to a bistable choice regime. In other words, strategies become path-dependent. This bistable structure is analytically tractable precisely because of the deterministic dynamics framework: fixed points and their stability can be identified exactly from the flow equations, whereas stochastic algorithms offer no such direct access to the stability landscape. By contrast, non-distributional agents that compute expected values of a continuous reward spectrum do not exhibit bistable regimes. This finding suggests a mechanistic account of choice hysteresis [74]. Choice hysteresis is the tendency to repeat past choices as a function of one’s history, reflecting path-dependence in an agent’s choice history [75]. Hysteresis in intertemporal choice has been documented from eye-tracking studies [76] to studies about addictive behaviors [77]. It has been shown that hysteresis in sequential decision-making increases in aging rats [78], and hysteresis has even been accounted for in terms of attractor models [79] — a description that aligns naturally with the bistable structure identified here.

In experimental psychology, there is a debate about whether optimism can be reduced to perseveration. While related, choice hysteresis and perseveration are distinct phenomena [80]: stuck-in-set perseveration [81] refers to the deployment of a previously appropriate strategy that is no longer goal-congruent. Several studies and meta-analyses have striven to disentangle optimism from perseveration in empirical datasets [75,82]. They concluded that both heuristics were present and irreducible to one another–but it was also shown that the optimism heuristic leads to higher performance in a wide range of two-armed bandit tasks, compared to perseveration [83]. These findings establish that the two phenomena are empirically distinguishable, but leave open the mechanistic question of what generates perseveration in the first place. DDRL provides a mechanistic answer. Bistability alone does not produce perseveration: it does so only when the agent is locked by its history into the lower-valued attractor–that is, when parameters lie on the suboptimal side of the value-ordering boundary (thick black line, Fig 4F). In that regime, the agent’s strategy is goal-incongruent; yet, in a stochastic learning system, noise-induced transitions across the basin boundary could in principle restore goal-congruent behavior. DDRL further predicts a more extreme case: in the individual dilemma, the better strategy is no longer a stable fixed point, and no noise-induced correction is possible. This constitutes inescapable stuck-in-set perseveration, consistent with accounts that trace perseverative behavior to constraints on policy representation [80,84]. Return discretization thus generates a hierarchy of path-dependent phenomena–from choice hysteresis, to escapable perseveration, to fully inescapable stuck-in-set perseveration–none of which arise in non-distributional dynamics.

The individual dilemma–the inescapable extreme of the perseverative hierarchy described above–offers a novel mechanistic account of incoherent choice. In analogy with the well-known social dilemma, an agent would be better off choosing the strategy that provides the highest value, but cannot do so because it is drawn toward the other, suboptimal strategy that is stable. Our results show that this situation arises in a wide range of combinations between discount factors and optimism weights. While individual dilemmas might appear as flaws in our model, there are many cases where human choices are inconsistent. Perhaps one of the most compelling illustrations lies in compulsive behavior. Compulsion occurs when one keeps engaging in actions that feel immediately relieving but are harmful in the long run. It is a key component of, e.g., Tourette’s syndrome and obsessive-compulsive disorder (OCD), and can occur even when patients are completely aware of the counterproductivity of their behavior. On the one hand, because of the ritualistic nature of compulsions, it has been suggested that habit plays a central role in compulsivity; on the other hand, there is a significant overlap between compulsivity and impulsivity–i.e., the reduced ability to delay rewards, which is quantified by the discount factor γ [85,86]. Our findings suggest there could be a complex interplay between reward discounting, optimism/pessimism, and discretization/habit in the emergence of compulsive behavior. Indeed, individual dilemmas appear for certain values of the discount factor and optimism weight. Thus, our framework could help disentangle the contributions of impulsivity, optimism and habit formation in giving rise to incoherent choice patterns. Moreover, our results show that an individual dilemma can be escaped by tuning up the optimism weight or the discount factor, depending on the region of the parameter space one finds oneself in. This opens the door to developing treatments which, by acting on impulsivity and/or optimism levels, could alleviate compulsive symptoms.

Empirically testing the behavioral predictions of DDRL — including the presence of individual dilemmas and their dependence on discount factor and optimism weight — would require fitting the framework to individual-subject trial-by-trial data. Adapting the deterministic dynamics to a stochastic likelihood model is non-trivial but constitutes an important direction for future empirical work.

Because of its distributional basis, our framework opens the door to representing decision-making heuristics as distortions on state-action return distributions. The optimism/pessimism distortion implemented here via the weights method is only one of several available approaches; cumulative prospect theory, CVaR, and the Wang distortion [87] provide alternative formal representations, and comparing outcomes across methods remains an open question. Among candidate heuristics, self-efficacy [88] and confirmation bias [59] could be interesting candidates to model in individual and collective scenarios. This would help address outstanding debates and questions in several disciplines. For instance, it has been suggested that optimism could be a confirmation bias in disguise, at the individual [59,89] and collective levels [90]. Thus, distinguishing between optimism and confirmation heuristics in individual and collective settings is an essential task for experimental researchers. In this regard, our framework could be used to generate hypotheses and predictions.

Besides empirical questions, our model could be applied to study the role of optimism/pessimism in complex collective-action problems, such as climate change. For tractability reasons, cognitive, social, and environmental factors are often studied in isolation, missing an integrated perspective [11]. Our model can contribute to such an integrated perspective. For example, it could enable insights into the influence of optimism/pessimism on cooperation in the face of climate tipping points [91]. Previous work found that the severity of the tipping impact, the certainty of the tipping threshold, and caring for future rewards are conducive to cooperation [37,92,93]. But what if these factors are not enough? Is it better to be optimistic, to induce coordination in the social dilemma, as we have seen in our social coordination task? Or is it better to be pessimistic and risk-averse, to avoid triggering the tipping element, as we have seen in our intertemporal risky-choice problem? DDRL is suited to address this tension: its deterministic dynamics yield an analytically tractable stability landscape — analogous to Fig 4F — in which the conditions for coordination, collapse, and bistability can be identified as functions of optimism weight, discount factor, and return discretization. The climate scenario combines both a coordination structure (as in our Stag-Hunt) and an intertemporal collapse risk (as in our risk-reward dilemma), and DDRL provides a natural framework for analyzing their interaction in a stateful multi-agent setting. More broadly, DDRL opens a route to studying how neurocognitive heuristics shape collective behavior across complex social-ecological systems [6,7,11].

We also highlight some limitations of our framework. First, although it builds upon stochastic games, which are the foundation of multi-agent reinforcement learning in machine learning [94], our framework does not scale to large environments (such as large grid worlds or 3D computer games). Such choice environments have large state and reward spaces, leading to a combinatorial explosion and the curse of dimensionality. The analysis of collective dynamics in such high-dimensional environments requires the use of more suitable methods. Second, our framework presupposes categorically discretized neurons. This limits the precision of approximating return distributions, compared to more sophisticated parameter-estimation methods used in the machine-learning literature [46,47]. Thus, our framework’s application to machine learning might be limited, as it is not an optimization method. It is a biologically inspired method of analysis designed to improve understanding. Third, the Gaussian smoothing projection used throughout is a biologically motivated choice — its graded, overlapping allocation reflects the tuning-curve structure of reward-sensitive neurons — but it is not the only option. Mean-preserving projections such as the Cramér projection [27,95] deterministically allocate mass to at most two adjacent atoms and are standard in categorical distributional RL. These projections would be integrable into our framework in principle, but would deviate from our initial motivation. Using a mean-preserving projection would align the framework more closely with standard categorical distributional RL, potentially opening it to a broader range of questions and applications.

In conclusion, our deterministic distributional multi-agent reinforcement learning framework bridges the gap between the microscopic constraints of neural reward encoding and the macroscopic dynamics of collective behavior. It offers a unified perspective through which seemingly disparate phenomena–from optimism and choice hysteresis to compulsive behavior and social coordination–can be understood as emergent properties of a common underlying mechanism. While we acknowledge the trade-off between tractability and computational scalability, our approach provides a necessary middle ground for cross-disciplinary inquiry across the animal, human, and machine behavioral sciences. Moving forward, this framework offers a robust toolkit for generating testable hypotheses and investigating how individual-level cognitive biases shape collective outcomes in complex real-world challenges, ultimately advancing our understanding of the intricate interplay between neural computation, individual choice, and collective dynamics.

## Supporting information

S1 Text Supplementary Information for “Deterministic dynamics of distributional multi-agent reinforcement learning. ” Contains full mathematical derivations, supplementary analyses, and all simulation code rendered as a self-contained document, ensuring that results remain reproducible independently of the availability of external code repositories.
(PDF)
