# Supplementary material for: Deterministic dynamics of distributional multi-agent reinforcement learning
Source: PLoS Comput Biol. 2026 Sep 23;22(9):e1014723. doi: 10.1371/journal.pcbi.1014723 (PMC13630324; doi:10.1371/journal.pcbi.1014723)
Supplement: Supplementary file 2 [file pcbi.1014723.s002.pdf]

Summary

This manuscript introduces a "distributional deterministic RL" (DDRL) framework that combines distributional reinforcement learning, where agents learn full reward distributions, with deterministic learning dynamics based on strategy averaging. Optimism and pessimism are implemented through distortions of the learned value distribution, and the model is evaluated across three canonical contexts: exploration-exploitation, social coordination, and intertemporal risky choice. In each case, the authors reproduce qualitative effects previously associated with optimism in related models.

The modeling framework is elegant and technically well presented. I particularly appreciate the care devoted to the methodological exposition and the accessibility of the code, which makes the model transparent and potentially useful as a tool for the reinforcement learning community.

At the same time, I have substantial concerns regarding the claimed scope and positioning of the contribution. The introduction is long and at times difficult to follow, and the central objective of the paper remains somewhat unclear. More importantly, several claims about the added value of DDRL in the study of heuristics and collective behavior appear stronger than what is directly supported by the analyses. In particular, the added value of this model relative to prior work is not clearly established. In its current form, the conceptual advance of this manuscript over the existing literature remains somewhat unclear. Below, I outline major points that, in my view, need to be addressed to clarify the paper's novelty, delimit its claims, and strengthen its relevance for cognitive scientists.

Introduction

- **Lines 22-23:** The statement that "an overarching explanation for [behavioral biases'] origin or adaptive character is lacking" feels somewhat too broad. While this may hold in a general sense, important explanations have been proposed for many specific biases (e.g., Gershman et al., 2015; Summerfield & Parpart, 2022; Shenhav et al., 2017), including within the heuristics tradition mentioned later in the manuscript. As written, the sentence may give the impression that the present study aims to provide a unifying account of all behavioral biases, which seems to extend beyond its actual scope. Clarifying this point would help position the contribution more precisely.

- **Lines 37-38:** The claim that "it is unclear why heuristics are so pervasive among humans and animals, and which brain mechanisms support them" overlooks a substantial body of existing work addressing these questions. Including relevant

prior studies would strengthen the introduction and avoid suggesting that this issue has not yet been explored.

- **Lines 66-67:** The statement that existing models treat optimism and pessimism as ad hoc parameters would benefit from further clarification, in order to clearly distinguish DDRL from them. As currently phrased, the sentence suggests that contrary to existing models, DDRL avoids ad hoc parameterization altogether, whereas optimism/pessimism appears to be implemented through a very specific distortion of the value distribution.

- **Lines 67-68:** The statement that previous models “cannot explain why specific levels of optimism or pessimism are helpful in a given situation” seems overstated. In RL, the impact of a feature can typically be quantified by comparing cumulative reward with and without that feature.

- **Lines 69-71:** The claim that distributional RL uniquely provides the “representational richness” required to meaningfully implement and reason about optimism/pessimism is not sufficiently justified. It is unclear why previously cited approaches would not allow meaningful implementation or analysis of these constructs.

- **Lines 75-76:** The sentence “doing so allows for improved treatment of uncertainty” is potentially misleading. It seems that the representation of the full return distribution that enables richer treatment of uncertainty, rather than the distortion of that distribution per se.

- **Lines 78-81:** The claim that the methodology allows agents to differentiate between environmental stochasticity and uncertainty due to insufficient exploration requires substantially more explanation. It is not clear how this distinction is implemented in the model. Moreover, the agents in the present study appear to have fixed levels of optimism/pessimism and thus cannot decide whether to be optimistic or pessimistic depending on the context.

- **Lines 84-86:** The statement that existing approaches have mostly been used in specific contexts (e.g., risky or collective choice) suggests that the present study goes beyond this limitation. However, the current manuscript also evaluates the model in specific contexts (albeit several of them). This distinction should be framed more accurately.

- **Lines 94-95:** The claim that the field lacks a “unified, biologically plausible framework for modeling heuristics in RL” again suggests a level of generality not reflected in the paper. The present study focuses specifically on optimism and pessimism, not on heuristics more broadly.

- **Lines 96-97:** The phrase “combining multi-agent reinforcement learning with complexity science and evolutionary dynamics” is unclear. It would be helpful to specify how “complexity” science is applied to the model in practice, and where evolutionary dynamics enter the analysis.

- **Lines 104-106:** The manuscript repeatedly refers to “collective behavior.” However, in the current implementation, agents interact only indirectly through reward contingencies and do not model or predict each other’s behavior. There is no explicit social inference or communication. Referring to this as “collective behavior” may therefore be misleading and should be reconsidered or more carefully defined.

- **Line 128-129:** The manuscript mentions that the deterministic aspect of the model can help understand the role of noise in learning, yet this issue is not revisited, and the impact of noise is not systematically explored.

Claim that perseveration results from neural reward discretization

In both the Abstract (lines 13–14) and the Discussion (lines 635–638), the authors suggest that perseveration may arise from low-level biological mechanisms such as distributed reward encoding, because this mechanism produces path dependence in the model’s strategy. However, while path dependence is necessary for perseveration, it is not sufficient. The manuscript does not clearly demonstrate that the model produces perseverative behavior in the behavioral sense, nor does it disentangle path dependence from perseveration per se. This claim would benefit from either additional supporting analysis or more cautious phrasing.

Comparison with behavior

The manuscript reproduces three empirical findings, previously captured by different models in distinct contexts, within a single framework. However, if the goal is to bridge machine learning and experimental psychology, it is essential to explain how the predicted qualitative effects are observed in human behavior (and not only in previous RL models).

Ideally, the authors could go further and fit the DDRL model to human behavioral data to demonstrate its empirical adequacy. At minimum, the manuscript should explicitly connect the simulated patterns to established behavioral findings. Without such comparison, it is difficult to assess the model's relevance for cognitive science.

Comparison with existing models

Although several reinforcement learning approaches are discussed and partially compared to DDRL (Figures 2B, 2C and 4D, 4E), the results are not fully contextualized. In the intertemporal risky choice task, DDRL predicts quantitatively different patterns compared to a non-distributional agent. However, it remains unclear which pattern aligns better with human data, or which is theoretically more plausible.

The authors argues their model is biological plausibility because it relies on distributed value encoding across simple units. However, classical RL models could also be implemented in population codes that represent expected values rather than full distributions.; biological inspiration alone does not necessarily establish greater plausibility. A more explicit conceptual or empirical comparison with alternative models would therefore, in my opinion, considerably strengthen the manuscript.

If relevant behavioral datasets are available, comparing model fits would be particularly informative. If such comparisons have already been performed elsewhere, discussing them would be valuable.

Applications to complex collective decision-making

In the Discussion (lines 665-678), the authors suggest that the model could be applied to complex collective decision-making problems such as responses to climate change. While this is an interesting perspective, the mechanisms described appear to rely primarily on risk-seeking versus risk-averse tendencies, which are already standard components of many decision-making models. It would be helpful to clarify what unique contribution the optimism/pessimism mechanism provides in this broader context.

Minor points

The choice to model a population of 29 neurons is quite unusual, is there any specific reason for that?
