# Supplementary material for: Deterministic dynamics of distributional multi-agent reinforcement learning
Source: PLoS Comput Biol. 2026 Sep 23;22(9):e1014723. doi: 10.1371/journal.pcbi.1014723 (PMC13630324; doi:10.1371/journal.pcbi.1014723)
Supplement: Supplementary file 3 [file pcbi.1014723.s004.pdf]

PLOS Computational Biology

Jun.-Prof. Dr. Wolfram Barfuss
Argelander Professor for
Integrated System Modelling
for Sustainability Transitions
University of Bonn

Phone: +49-228-73-4476
Email: wbarfuss@uni-bonn.de
Web: https://wbarfuss.github.io

May 27, 2026

PCOMPBIOL-D-25-02733
Deterministic dynamics of distributional multi-agent reinforcement learning

> Dear Dr. Barfuss,
> Thank you for submitting your manuscript to PLOS Computational Biology. After careful consideration, we feel that it has merit but does not fully meet PLOS Computational Biology's publication criteria as it currently stands. Therefore, we invite you to submit a revised version of the manuscript that addresses the points raised during the review process.
> The paper was read by the editorial team, as well as by two reviewers who provided detailed comments. In general, there was an appreciation of the detailed modeling work, but the reviewers highlighted some deficiencies in the writing. Especially (but not exclusively) the description of the modeling framework (reviewer 1) and the positioning of the contribution relative to the literature (reviewer 2) would need to be better explained in a resubmission.
> [...]
> We look forward to receiving your revised manuscript.
> Kind regards,
> Ulrik R. Beierholm, Academic Editor, PLOS Computational Biology
> Andrea E. Martin, Section Editor, PLOS Computational Biology

Dear Dr. Beierholm and Dr. Martin,

Thank you for the opportunity to revise our manuscript. We have carefully addressed all comments raised by both reviewers. Following Reviewer 1's feedback, we have corrected several technical inaccuracies in the derivations, added explicit justification for each methodological choice, and restructured the methods section to separate background material from new contributions. Following Reviewer 2's feedback, we have substantially revised the Introduction to tighten scope claims, better position the contribution relative to the literature, and add empirical connections to each of the three task domains. Additionally, and following correspondence with Nithya Venkatesan from the Editorial Office, we have replaced the left side of Figure 1 with a newly created original illustration to fully resolve any copyright concerns. We have also restructured the sections to comply with PLOS Computational Biology's guidelines: 'Methods' and placed before 'Results', which we consider more appropriate for a theoretical contribution where the notational conventions must be established before the main results can be appreciated. The former subsection '2.2 Deriving deterministic distributional reinforcement learning dynamics' now opens 'Results' as its first subsection to better highlight our paper's contribution. The three task-domain sections follow as further subsections, each prefixed 'Example application:'. Lastly, we have referenced and included our Supplementary Information in

accordance with the journal's guidelines. Let us thank all members of the editorial office here for their helpful guidance.

Detailed responses to each comment of the reviewers are provided below.

We hope that the revised manuscript now meets the journal's publication criteria, and we look forward to hearing from you.

With kind regards,

Wolfram Barfuss, on behalf of all authors

Response to Reviewer 1

Comment 1.1

Main claims of paper
This paper studies multi-agent interactions from the perspective of stochastic games, a well-established approach in game theory. The paper is specifically concerned with the behavior of agents that interact and learn through the use of distributional reinforcement learning algorithms, and the use of such a framework in modeling and understanding heuristics in human decision-making.
From a methodological point of view, the primary focus of the paper is on a variant of the categorical approach to distributional reinforcement learning, with a specific choice of rule for inducing behavior based on optimistic/pessimistic summaries of learned distributions. The authors focus on a simplified model of the multi-agent interactions, in which discrete updates are made on the basis of expected behavior (rather than random updates that may arise from e.g. agents randomly sampling actions, or stochastic environments).
Interactions are simulated in three domains, and several connections are drawn between the methodological parameters, such as reward discretization and discount factor, and phenomena arising in human-decision making, such as perseveration, choice hysteresis, and "individual dilemma".
High-level discussion
The direction of building a framework to model aspects of human decision-making through distributional reinforcement learning is an interesting, novel one. In this regard, the work naturally complements prior work on distributional reinforcement learning in machine learning and neuroscience. I expect the emphasis here on studying the dynamical systems that arise from distributional learning algorithms in a variety of contexts to spur on future work in this area.
That said, I think the paper would benefit from expanded discussion of the framework that the authors introduce here. In particular, there are a few specific choices made (categorical approximations of distributions, use of deterministic dynamics, a specific form of optimism/pessimism), and it was not clear to me whether the authors are advocating for these choices over others (or just making one of many possible choices). For example, it would also be possible to investigate what happens when a quantile-based approximation of distributions is used, with a different form of optimism/pessimism, and simulating dynamics that take into account policy randomness. I think exhaustively examining other possibilities like this is beyond the scope of the paper, and studying the consequences of one possible set of choices is a nice contribution; I think though it would strengthen the paper to emphasize that these other choices exist, to what extent the authors expect the findings to translate to these other settings, and why the paper

focuses on the choices made here.
The simulation findings in Sections 3.1/3.2 replicate earlier findings regarding benefits of optimism/pessimism depending on the reward structure of the environment. I found the findings in Section 3.3 regarding the interaction of discount factor and optimism levels, and resulting (bi)stability of policies interesting, and I expect there to be interest in investigating these phenomena further in future work.
There are several deviations in the methodology presented here from standard distributional RL practice. Some of these deviations (in particular, using discretization rather than Cramer projections in approximating reward/return distributions) seem important in several of the claims, so it would be useful to have further discussion of these choices in the paper. Further details are given below.
The authors have provided detailed explanations and code in the supplementary material that greatly aids understanding of the nuances of the simulation protocols, and should be highly useful to other researchers in building on this work.

Response. We thank the reviewer for a careful and constructive reading. We have addressed each comment in detail below. In brief, we have added justification for the methodological choices, corrected several technical inaccuracies in the derivations, and clarified deviations from standard distributional RL practice that went unaddressed.

Main comments and concerns

Comment 1.2

Some lack of discussion of choices made
As mentioned in the high-level discussion above, there are several methodological choices made in the paper that could benefit from further discussion; in brief, why are these specific choices made, and what might we expect to find if we make other choices? Some important examples are given below.

- Eqn (6) commits to an exploration policy given by a Boltzmann distribution. However, there is no discussion of why this choice is made over e.g. epsilon-greedy exploration, or more involved approaches such as UCB-based exploration.
- The work commits to a categorical representation of the return distribution. Other representations are available, such as quantile-based approaches (Dabney et al., 2018), or approaches that learn other properties of distributions, such as expectiles.
- In Section 2.1.4, the paper describes analyzing learning dynamics with the noise in TD updates integrated out. Further discussion as to why this choice is made would be very beneficial; for example, presumably this approach makes simulating dynamics more straightforward and easier to replicate. On the other hand, it is not clear what might be lost by using this simplification of the dynamical system. An important case for this paper might be when both agents in a symmetric two-player game are initialized to the same strategy (as in Section 3.2). Under deterministic dynamics, their strategies remain coupled throughout the dynamics, but in real stochastic play, they will become uncoupled. Does this more general situation in the stochastic case change the conclusions of the paper? Perhaps a brief discussion/comparison to the “ODE method” in dynamical systems

> could be made (e.g. Borkar, 2008 as a general reference, or Tsitsiklis and Van Roy, 1997, for specific application to reinforcement learning); this technique also uses deterministic dynamics to analyse behavior of dynamical systems, and provides results that guarantee the same asymptotic behavior of stochastic dynamics and their deterministic counterpart, under certain conditions.
>
> - The use of Gaussian smoothing of returns in Section 2.2.1 is introduced without discussion of why this choice is made. This is not a default approach in distributional reinforcement learning, so it would be interesting to discuss why the choice was made here. There is however some precedent for this idea in distributional RL (see below) which could be cited.
> - Section 2.3 defines one way of obtaining optimistic/pessimistic value summaries of a distribution (although the definition is somewhat unclear; see below). It's unclear why this precise choice is made, how alternatives would affect the conclusions of the paper, and how this choice compares to earlier approaches in distributional RL (such as the use of expectiles in Rowland et al., 2021, or distortion risk measures in Dabney et al., 2018b).
>
> In summary, there are a few particular methodological choices here (categorical distributions, particular types of optimistic/pessimistic distribution summaries) that I can imagine researchers in future expanding on (for example, by using quantile-based representations of distributions, or using conditional-value-at-risk as an optimistic summary). The paper's impact and utility for these future works could be increased with some indication that other methodological choices are possible, and perhaps more discussion of why the choices made in this paper are reasonable first choices to study.

Response. We thank the reviewer for raising these points; they improve the paper's transparency. We address each sub-point in turn.

Exploration scheme (Eq. 6). We concede that the choice of Boltzmann policy was previously unjustified. We have added a paragraph to Section 2.2 (old: 2.1.2) explaining the choice on three grounds. First, it follows from the maximum-entropy principle (Jaynes and Bretthorst, 2003): among all distributions over actions consistent with current Q-value beliefs, the Boltzmann distribution is the one that maximises entropy — committing to no assumptions beyond those beliefs — and is therefore the least arbitrary choice given the agent's current state of knowledge (Wolpert et al., 2012). It has also been connected to reward-matching behaviour in animal and human choice (Herrnstein, 1961; Soltani and Wang, 2006). Second, the policy is particularly convenient for the deterministic dynamics framework (Section 2.4, old: 2.1.4), since the strategy update depends only on differences in Q-values across actions, action-constant quantities cancel, substantially simplifying the dynamics. Third, while ε-greedy policies can also be combined with strategy averaging (Barfuss and Meylahn, 2023), they expand the dynamical state space. More elaborate schemes, such as UCB, complicate the dynamics further still, to the point of analytical intractability, unless the exploration process itself is the primary object of study. The Boltzmann policy is therefore the simplest choice that is both principled and compatible with the deterministic dynamics approach.

Return-distribution representation (categorical vs quantile/expectile). We have replaced the phrase "for the sake of simplicity" with an explicit justification in Section 2.3 (old: 2.1.3). Categorical DistRL uses a fixed discretization support, which maps naturally onto a population of neurons each tuned to a specific return value — a stable property throughout learning. Quantile- and expectile-based representations (Dabney et al., 2018b; Rowland et al., 2019) use adaptive atoms that shift during

learning, improving approximation quality but with no clear neural analogue. More fundamentally, adaptive atoms would render the deterministic dynamics derivation of this paper incompatible: the representational state itself would become time-varying, precluding the fixed-point strategy-averaging approach on which the derivation rests.

Deterministic vs stochastic dynamics. We agree that the rationale for the deterministic limit deserved explicit discussion. We have added a paragraph to both the Introduction (Section 1) and to Section 2.4 (old: 2.1.4) explaining the choice. Deterministic RL dynamics, with a formalism analogous to evolutionary game theory (Hofbauer and Sigmund, 1998; Börgers and Sarin, 1997), yield analytically tractable trajectories in strategy space and enable the exact characterisation of fixed points, their stability, and bifurcations. Stochastic dynamics can be understood as a noisy counterpart: the stationary distribution of the stochastic system concentrates around the stable fixed points of the deterministic part and spends negligible time near unstable ones (Barfuss, 2022; Barfuss and Meylahn, 2023). The deterministic approximation therefore captures average learning behaviour while abstracting away intrinsic fluctuations — a limitation we already acknowledge in the Discussion. Regarding the reviewer's point about symmetric initialisation in Section 3.3 (old: 3.2): under deterministic dynamics, identically initialised agents follow identical trajectories; in stochastic play, noise would break this coupling. However, the qualitative attractor structure — which attractors exist and the shape of their basins — is determined by the deterministic dynamics and is robust to this symmetry breaking. Concerning the ODE method (Borkar, 2008; Tsitsiklis and Van Roy, 1996): this is a related but distinct approach, where the ODE serves as an asymptotic tool to establish convergence of the underlying stochastic recursion algorithm, given some assumptions, e.g., a decreasing learning rate. Our approach differs in that we directly formulate and study the deterministic strategy update as a discrete-time dynamical system, without relying on a slow-learning limit.

Gaussian smoothing. The Gaussian projection was chosen on biological grounds: reward-sensitive neurons have overlapping tuning curves (van Duuren et al., 2008; Ferrari-Toniolo and Schultz, 2023) and therefore respond to nearby values, producing a graded, distributed activation rather than a binary one. A Gaussian kernel captures this naturally, with width σ encoding representational precision. The choice is also analogous to label smoothing in supervised learning (Imani and White, 2018) and has been applied in distributional RL by Farebrother et al. (2024) — prior work we now cite in the manuscript.

Optimism/pessimism mechanism (weights vs expectile/distortion risk measures). We concede that the choice of distortion method deserved explicit justification. We have added a paragraph to Section 3.1.7 (old: 2.3) explaining the choice on two grounds. First, the weights method is formally equivalent to asymmetric RL on the two-armed bandit task: a weights-DDRL agent with upper weight w_U reaches the same steady-state Q-values as an asymmetric-RL agent with bias ratio $b = \alpha^+/\alpha^- = w_U$ (proven in SI-Section 12.2), directly grounding the DDRL optimism parameter in the established cognitive science literature (Cazé and van der Meer, 2013). Second, in SI-Section 12.1 we compare the weights method against quantile- and expectile-based distortions of the return distribution applied to the same fixed categorical support. The quantile method exhibits discontinuous, non-smooth parameter dependence — an artefact of the discrete-distribution quantile being a step function of τ that can only jump by whole-atom increments — whereas the weights method yields smooth, monotonic dependence on w_U that closely tracks asymmetric RL across the full bias-strength range. Expectile-based distortions (Rowland et al., 2021) are smoother than quantile-based ones and admit a theoretical equivalence with asymmetric RL in stateless environments, but do not match as closely in practice — likely also because of our categorical discretization. We did not implement the distortion risk measures studied in Dabney et al. (2018a) (CVaR, Wang risk measure); we acknowledge these as

open alternatives in a new sentence added to the Discussion (Section 4, Other heuristics and future directions).

Comment 1.3

Unclear derivations/possible inaccuracies

- Reward discretization. In the introduction (Line 111), the authors mention discretization of rewards as a property of their framework in line with previous literature, citing Dabney et al.'s work on dopaminergic modelling. However, in this cited work, as in the vast majority of distributional RL literature, rewards themselves are typically not discretized. In general, returns may be discretized with a categorical projection (see below), or some other encoding scheme may be used; Dabney et al. study a model in which a discrete set of expectiles are learnt. From my reading, I think the current paper over-emphasizes how commonplace reward discretization is in distributional RL, and the paper would benefit from clarification here.

- Eqn (5): I don't think this Bellman equation holds for Q__t, since Q__t is in general an arbitrary estimate of the Q-function.

- Eqn (11): I don't think the move from the first line to the second line is valid. I think it relies on a Bellman equation being satisfied by Q__{X,t}, but I don't believe one holds, because Q__{X,t} is just an approximation to Q__X.

- I don't understand the discussion in Section 2.3. The definition of w at Lines 354-356 seems to have an unbounded v variable on the right-hand side. The inequalities here seem to be comparing a return outcome (r__k) to a probability (Q^{i,s,v}__X). I expect there may be a typo here.

Response. The reviewer is right on all three counts. We have re-checked the derivations and revised them as follows.

Reward discretization. We agree that reward-level discretization is uncommon in the distributional RL literature, where the standard object is the return distribution. We address this overlap of issues — the discretized object, the projection rule, and the "perseveration from discretization" claim — together in the substantive rewrite that responds to Comment 1.4 (and Reviewer 2's Comment 2.14); see the response there for the full set of changes. In short, we now align our exposition with the convention that returns (not rewards) are discretized.

Eq. 5, Bellman for Q_t. The reviewer is correct: the time-indexed estimate Q_t does not satisfy the Bellman equation as an identity — it is precisely the deviation from this fixed point that the TD errors in Eqs. 2 and 3 measure. The standalone Bellman equation in Eq. 5 was stated as a motivation for the form of the TD error, but the way it was written confused fixed-point identity with estimator identity. We have **deleted Eq. 5** and the surrounding sentences. We have also removed the parallel "Given that $Q_t = (1-\gamma)r + \gamma Q_t$" clause that preceded the Actor-Critic TD error in the previous version, and now present Eq. 3 directly as a definition: AC's TD error replaces SARSA's $Q_t^{s_t,a_t^i}$ baseline with the state-value $V_t^{s_t}$.

Eq. 11, line 1 → line 2. The substitution $(1-\gamma^i)R^{i,s,a^i}_{\mathbf{X},t} + \gamma^i \, {}^{\text{next}}Q^{i,s,a^i}_{\mathbf{X},t} \to Q^{i,s,a^i}_{\mathbf{X},t}$ is in fact an identity by construction: in the deterministic limit there is no estimation error, so the strategy-averaged Q-values always equal the true on-policy Q-values evaluated at the current strategy and therefore

satisfy the recursive Bellman equation (Eq. 10) exactly. We agree that the original notation $Q_{\mathbf{X},t}$ invited a misreading as an estimate (analogous to the time-indexed estimate Q_t in stochastic SARSA). To remove the ambiguity, we have **switched to the notation** $Q_{\mathbf{X}_t}$ (and $R_{\mathbf{X}_t}$, ${}^{\text{next}}Q_{\mathbf{X}_t}$, $V_{\mathbf{X}_t}$, $E_{\mathbf{X}_t}$) throughout the deterministic-dynamics section, making explicit that the time index is inherited entirely through the strategy $\mathbf{X}_t$ and that these are policy-conditional ground-truth quantities, not estimates. We have added an explanatory paragraph after Eq. 10 stating this convention, so that the algebraic step from line 1 to line 2 of Eq. 11 is recognised as an identity rather than as an approximation requiring a Bellman fixed-point assumption on Q_t.

Section 3.1.7 (old: 2.3), Definition of w. The reviewer correctly identified two related typos: the threshold variable carried an unbound v index on the RHS, and the inequalities compared a reward bin r_k with a quantity that — as written — was a probability rather than a scalar. The intended threshold was the *expected value* of the action-averaged return distribution, i.e. a scalar. We have **rewritten the equation that previously defined** $Q_{\mathbf{X}}^{i,s,v}$ so that it now defines the scalar

$$V_{\mathbf{X}}^{i,s} \;=\; \langle V_{\mathbf{X}}^{i,s,v} \rangle_v \;=\; \sum_v v \sum_{a^i} X^{i,s,a^i}\, Q_{\mathbf{X}}^{i,s,a^i,v},$$

and have updated the inequalities in the bullets that follow accordingly: $r_k \lessgtr V_{\mathbf{X}}^{i,s}$. The new form makes the dimensional comparison transparent (reward bin vs. scalar return) and ties back to the state-value distribution $V_{\mathbf{X}}^{i,s,v}$ defined in Eq. 18 by the identity $V_{\mathbf{X}}^{i,s,v} = \sum_{a^i} X^{i,s,a^i} Q_{\mathbf{X}}^{i,s,a^i,v}$, addressing Comment 1.10's point about Q vs. V on the LHS at the same time.

Comment 1.4

Non-standard distributional RL algorithms

- One important deviation from the distributional RL literature is that this paper describes discretizing reward distributions. In distributional RL, it is typically return distributions that are discretized (as in e.g. Bellemare et al., 2017), rather than reward distributions. Further, rather than the nearest-neighbor discretization described here, which allocates each reward to the neuron corresponding to the closest value, categorical distributional RL methods typically use a projection that preserves the mean (see for example Eqn (7) of Bellemare et al., 2017, or Eqn (7) of Rowland et al., 2018).

- There are therefore two important differences from standard categorical distributional RL algorithms: discretization of reward, rather than return, and discretization using a nearest-neighbor projection, rather than a mean-preserving projection. The latter may be quite important to the conclusions of the paper, since reward discretization is a hypothesized mechanism for several observations reported in the paper.

- In addition, the idea of using a Gaussian smoothing kernel in the context of distributional RL is explored by Farebrother et al. (2024), who in turn took this approach from Imani and White (2018) who used it in the context of supervised learning. This relates more generally to the concept of label smoothing in machine learning and statistics; some discussion of this prior work would be helpful in contextualizing the contributions of the paper.

- I don't think it's necessary for the paper to align all algorithmic choices with the most commonly used ones in distributional RL in the context of machine learning, but some discussion about where deviations are made would aid the clarity of the paper.

Response. We address the reviewer's three points in turn.

Reward vs. return atoms. The reviewer correctly observes that standard categorical DistRL — e.g. C51 (Bellemare et al., 2017) — places atoms over the return range $[V_{\min}, V_{\max}]$, whereas our atoms are defined at reward values $r_1, \dots, r_n$. The two coincide in our framework by design. Under the normalized gain convention $G = (1-\gamma)\sum_{k\geq 0}\gamma^k r_{t+k}$ used throughout the paper, returns are convex combinations of rewards and therefore lie in $[r_1, r_n]$. We have added a sentence to Section 2.3 (old: 2.1.3) (Distributional reinforcement learning) making this explicit.

Projection: Gaussian smoothing, not nearest-neighbor. Our discretization uses Gaussian smoothing throughout — for both the initial reward discretization ($P_\sigma(r_k|\tilde{r})$, Section 3.1.1 (old: 2.2.1)) and the Bellman projection ($\delta^{i,v,r,\acute{v}} = P_\sigma(v|(1-\gamma)r + \gamma\acute{v})$, Section 3.1.3 (old: 2.2.3)). According to our understanding, nearest-neighbor would concentrate all probability mass at the single closest atom; Gaussian smoothing distributes it across neighboring atoms with Gaussian weights — a softer and categorically different assignment. The reviewer may have read the Gaussian kernel as an additional smoothing step applied on top of nearest-neighbor binning, but it is the projection itself. We have clarified this in the manuscript.

The biological motivation and prior-art citations are addressed in our response to Comment 1.2 above.

Projection alternatives. We have added a paragraph to the Discussion (Section 4 (old: 4), Limitations) comparing Gaussian smoothing to the Cramér projection. Mean-preserving projections such as Cramér deterministically allocate mass to at most two adjacent atoms — standard in categorical distributional RL for algorithmic reasons — but lose the soft, graded character that motivates our biologically inspired choice. Such projections would be integrable into our framework in principle, and using them would align it more closely with standard categorical distributional RL, potentially opening it to a broader range of questions and applications. We do not claim that qualitative results would be unchanged under alternative projection rules.

Terminology. We retain "reward neurons" where the biological framing is primary (Section 3.1.1 (old: 2.2.1) and Fig. 1 caption) and use "return distribution atoms" where the distRL framing is primary. This is also addressed in our response to Comment 1.10.

Comment 1.5

Experiments/Section 3.1.
This is introduced as an exploration/exploitation challenge, but the agents are not evaluated on a metric that introduces such a trade-off (a classical example would be regret minimization, or comparison of cumulative reward). Instead, the policy at convergence is compared. This could be clarified by de-emphasizing the notion of exploration/exploitation in the discussion in this section.
"DDRL closely approximates the behavior of an asymmetric RL agent". As the authors mention, from Fig 2B-C (and the proof in the supplementary material), it looks like there is exact agreement here. As a general comment (not necessary to address in revisions), it would be interesting to know if there is exact agreement in the dynamics of the two approaches, or just in their limiting strategies.

Response. We agree. The section studies the asymptotic strategy under varying resource availability, not a regret- or cumulative-reward-based exploration–exploitation tradeoff. We have renamed the section "Bandit choice under resource variability" and replaced the two opening sentences accordingly.

SARSA vs Actor-Critic. The original phrase "accounts for exploratory behavior" was an imprecise attempt to convey a real distinction. SARSA dynamics with a Boltzmann policy are analogous to the replicator-mutator equation: an entropy term in the prediction error acts as a permanent mutation pressure, keeping asymptotic strategies mixed. Actor-Critic dynamics, by contrast, are analogous to the pure replicator equation, which drives strategies toward deterministic fixed points. In a two-armed bandit, an Actor-Critic agent would therefore converge to always choosing one arm, precluding the graded strategy curves in Fig. 2B–E. We have replaced the original sentence with this clarification, and added the replicator-mutator / pure-replicator distinction to Section 2.4 (old: 2.1.4) to prepare the reader.

Exact vs approximate agreement. The asymptotic strategies agree exactly: SI-Section 12.2 proves that a DDRL agent with weight w_U converges to the same fixed-point strategy as an asymmetric-RL agent with bias ratio $b = w_U$. We retain "closely approximates" for the overall behavioral claim because the transient dynamics are not directly comparable: Cazé and van der Meer (2013)'s derivation operates in a stochastic small-learning-rate limit and only applies to the steady state, whereas DDRL derives fully deterministic dynamics. Comparing the transient trajectories would conflate two different mathematical objects.

> Comment 1.6
>
> *Experiments*/Section 3.2.
> In Section 3.2, some specific values are given for the Stag-Hunt rewards. Why are these specific values used? Do they have precedent in the literature?

Response. The specific values are illustrative examples chosen to satisfy the canonical stag-hunt ordering $R > T > P > S$. They are not drawn from a particular empirical paradigm. The qualitative conclusions — which Nash equilibria exist, whether optimism shifts the stable attractor from mutual defection to mutual cooperation, and the existence of an unstable mixed-strategy fixed point — depend only on this ordering, not on the specific magnitudes. Basin sizes do vary with the exact values, so we do not claim quantitative robustness beyond the ordering constraint. We have added a parenthetical note to the figure caption making this explicit.

> Comment 1.7
>
> *Experiments*/Section 3.3
> I found the bistability that arises here very interesting. My understanding is that this is one of the benefits of studying the deterministic dynamics of the learning algorithm; the concepts of stability are much easier to pin down than if we were dealing with stochastic algorithms (e.g. incorporating randomness from the agent's policy), because certain conditions are required to guarantee convergence in these cases. If my understanding is correct, it could be nice to emphasize this point here, and when introducing the deterministic dynamics perspective earlier in the paper, to communicate to readers why these choices are made.
> The point in this section about discretization of the reward signal playing a key role in stability, in reference to Fig 4E, was not clear to me. As far as I can tell, the number of distributional predictions, and degree of discretization, is not varied in this experiment, so I am unsure how this conclusion is arrived at. It would be great if the authors can expand on this.

Response. We thank the reviewer for this opportunity to clarify and strengthen these points.

Bistability and deterministic dynamics. The reviewer's understanding is exactly right: the deterministic

dynamics framework enables a clean analytical characterisation of the stability landscape that would be inaccessible under stochastic algorithms. Because fixed points can be identified exactly from the flow equations and their stability assessed from the sign of dX, the bistable regime in Section 3.4 (old: 3.3) can be mapped exhaustively across parameter space (Fig. 4F). We have added a sentence to the Discussion (Section 4 (old: 4), bistability paragraph) making this explicit: "This bistable structure is analytically tractable precisely because of the deterministic dynamics framework: fixed points and their stability can be identified exactly from the flow equations, whereas stochastic algorithms offer no such direct access to the stability landscape." The general rationale for the deterministic dynamics approach — including the relationship to the stochastic case — is stated in the Introduction and Section 2.4 (old: 2.1.4) in response to Comment 1.2; the new Discussion sentence ties the specific finding back to that broader motivation.

Fig. 4E and the role of discretization. The reviewer is right that no discretization parameter is varied within Fig. 4E. The confusion arises because the argument is *eliminative* — a comparison between two agents — rather than variational. The two panels use:

- **Fig. 4D**: a non-distributional DetRL agent, operating on expected values from a continuous return spectrum with no discretization.
- **Fig. 4E**: a neutral DDRL agent ($w_U = 1$), using the discretized return distribution but with the optimism/pessimism bias switched off.

Setting $w_U = 1$ rules out optimism or pessimism as the cause of bistability; the only remaining structural difference between the two panels is then the discretization of the return distribution. Since only Fig. 4E exhibits bistability, discretization is identified as the driver by elimination. We have added a sentence to Section 3.4 (old: 3.3) making this chain of reasoning explicit before the conclusion statement: "The neutral weight $w_U = 1$ rules out optimism or pessimism as a cause; the only remaining structural difference between Fig. 4D and Fig. 4E is therefore the discretization of the return distribution."

Detailed notes

Comment 1.8

Optional/minor formatting notes
Using superscript on discount factors to index by agent, as well as for exponents, could cause confusion. Does this paper require the introduction of different discount factors for each agent? I don't believe so, and in this case, Section 2.1.1 could introduce stochastic games with a global discount factor and avoid introducing this notation.
The formatting of Line 140 seems odd. Is there a full stop after ">" which is missing/obscured by the line number?
Section 2 is very long, and contains both review material and new contributions. The authors could consider splitting this section into a few smaller sections, making clearer the distinction between new and existing material.
Line 332: "Noting" -> "Denoting".

Response.

On agent-indexed discount factors. We agree that all examples presented in the paper use homogeneous parameters. However, after careful consideration, we have decided to retain the agent superscript γ^i in

the general framework. The learning rate α^i and choice-intensity parameter β^i also carry agent indices throughout — dropping γ^i alone would create a notational asymmetry without principled justification. In the behavioral modeling context the paper targets, individual differences in temporal discounting are empirically well-documented and meaningful, and stating the framework in full generality — with $\gamma^i = \gamma$ recoverable as a special case — is deliberate. The section restructuring, described below, additionally highlights the framework in its own right.

On the length and structure of the methods. We thank the reviewer for this constructive suggestion. In compliance with PLOS Computational Biology's section guidelines, the DDRL derivation and bias mechanism are gathered under a subsection 'Theoretical Framework', which opens the 'Results' section. The three example task domains appear as further subsections of 'Results', each prefixed 'Example application:' to clarify their illustrative role.

On the formatting of line 140. We added a line break.

On "Noting" → "Denoting." Corrected.

> Comment 1.9
>
> *Citations*
> In providing references for distributional reinforcement learning, the authors could consider citing earlier work than Bellemare et al. (2017) as well, such as Morimura et al. (2010a;b).

Response. We thank the reviewer for this suggestion. We have added citations to Morimura et al. (2010a;b) at the opening of the DistRL background subsection, where we now acknowledge that distributional approaches to RL predate the categorical formulation of Bellemare et al. (2017).

> Comment 1.10
>
> *More minor technical notes*
> This section notes technical issues with the presentation, though ones which do not affect the key messages of the paper.
> Eqn (7): Based on the conventions of the paper, the R^{s,a} term is missing a factor of (1-gamma^i).
> "We assume an agent has $n \in \mathbb{N}$ reward"neurons", each neuron being sensitive to a given range of amount of reward." Should "reward" be "return"?
> I don't understand the discussion prior to Eqn (23) that the critic can be removed in the definition of the temporal-difference error. If we remove the critic, it seems that what we are measuring is no longer an error between predicted and realized returns?
> Eqn (24): Why not use "V", rather than "Q", on the left-hand side, since this is an action-independent quantity?
> Line 436: "However, if both agents defect, they receive the sucker's payoff S" Shouldn't this be "However, if both agents defect, they receive the punishment payoff P"?

Response. We thank the reviewer for these careful technical observations. Each is addressed below.

Eq. 7, missing $(1-\gamma^i)$ *factor.* The reviewer is correct. Throughout the manuscript we use the normalised gain convention $G_t^i = (1-\gamma^i)\sum_{k\geq 0}(\gamma^i)^k r_{t+k}^i$ (cf. the gain definition in Section 2.1 (old: 2.1.1)), under which the Bellman target carries an explicit $(1-\gamma^i)$ on the immediate reward — visible, for instance, in Eqs. 2, 3, and 10. Eq. 7 had been transcribed in the non-normalised form used in Bellemare et al. (2017) and was therefore inconsistent with our convention. We have **added the**

$(1-\gamma)$ **factor** so that Eq. 7 now reads $\mathcal{Q}^{s,a} \overset{D}{=} (1-\gamma)R^{s,a} + \gamma\mathcal{Q}^{\acute{s},\acute{a}}$, and we note the convention explicitly in the surrounding text.

"reward" vs. "return" neurons (Section 3.1.1 (old: 2.2.1), originally line 244). This wording is part of the broader question of whether our model discretises rewards or returns — the same point raised in Comment 1.4 and Reviewer 2's Comment 2.14. We resolve all three together in our response to Comment 1.4. In brief, we retain "reward neurons" in Section 3.1.1 (old: 2.2.1) and the figure caption where the biological framing is primary, and use "return distribution" where the DistRL framing is primary; see the Comment 1.4 response for the full discussion.

Critic removal in the deterministic-limit TD error (pre-Eq. 23). The reviewer's concern is well-taken: removing the critic does mean that what remains is no longer literally a "TD error" in the variance-reduction sense — it is the *action-dependent component* of the TD error, with the action-constant critic $\langle V^{i,s,v}_{\mathbf{X}_t} \rangle_v$ omitted. The justification, inherited from DetRL (Barfuss et al., 2019), is that since the omitted term depends only on $(i, s, \mathbf{X}_t)$ and not on a^i, it cancels in the Boltzmann strategy update and therefore the deterministic policy dynamics are identical whether or not we keep it. We have **added a clarifying sentence** before Eq. 23 making this explicit.

Eq. 24, Q vs. V on the LHS. The reviewer is correct that the LHS quantity is action-independent and so should carry a V, not a Q. As described in our response to Comment 1.3, we have rewritten this equation: it now defines the scalar $V^{i,s}_{\mathbf{X}} = \langle V^{i,s,v}_{\mathbf{X}} \rangle_v$ used as the threshold for the weights step, and we note the equivalence $V^{i,s,v}_{\mathbf{X}} = \sum_{a^i} X^{i,s,a^i} Q^{i,s,a^i,v}_{\mathbf{X}}$ with the state-value distribution defined in Eq. 18. This addresses both the Q/V issue and the unbound-v issue raised in Comment 1.3 in a single edit.

Line 436, sucker → punishment. Corrected; thank you.

Comment 1.11

Terminology
The manuscript makes use of the term "value distributions" to refer to the distributions learned in the paper. While Bellemare et al. (2017) make use of this term, earlier and later work both use the more technically correct "return distribution".

Response. We thank the reviewer for pointing us to this. We agree: in classical RL, "value" denotes the expected return — a scalar — so "value distribution" conflates a distribution over returns with its own expectation; "return distribution" is more precise. We have replaced all generic uses of "value distribution" with "return distribution" throughout, including the bolded introductory use in Section 2.3 (old: 2.1.3) where the distributional Bellman equation from Bellemare et al. (2017) is presented. For our two named mathematical objects, $Q^{i,s,a^i,v}_{\mathbf{X}}$ and $V^{i,s,v}_{\mathbf{X}}$, we decided on the compound terms "Q-return distribution" and "V-return distribution" respectively: these are the distributional analogues of the classical Q- and V-functions, and the compound names make that analogy explicit. The distRL literature does not use fixed compound names for these objects — Bellemare et al. (2023) simply write "return distribution associated with the state-action pair" when disambiguation is needed — so we introduce our own terms for brevity and internal consistency.

References

Borkar, V.S, 2008. Stochastic approximation: a dynamical systems viewpoint. Cambridge University Press.

Dabney, W., Ostrovski, G., Silver, D. and Munos, R., 2018b. Implicit quantile networks for distributional reinforcement learning. In Proceedings of the International Conference on Machine Learning.
Farebrother, J., Orbay, J., Vuong, Q., Taïga, A.A., Chebotar, Y., Xiao, T., Irpan, A., Levine, S., Castro, P.S., Faust, A. and Kumar, A., 2024. Stop regressing: Training value functions via classification for scalable deep RL. In Proceedings of the International Conference on Machine Learning.
Imani, E. and White, M., 2018. Improving regression performance with distributional losses. In Proceedings of the International Conference on Machine Learning.
Morimura, T., Sugiyama, M., Kashima, H., Hachiya, H. and Tanaka, T., 2010a. Nonparametric return distribution approximation for reinforcement learning. In Proceedings of the International Conference on Machine Learning.
Morimura, T., Sugiyama, M., Kashima, H., Hachiya, H. and Tanaka, T., 2010b. Parametric return density estimation for reinforcement learning. In Proceedings of the Conference on Uncertainty in Artificial Intelligence.
Rowland, M., Bellemare, M., Dabney, W., Munos, R. and Teh, Y.W., 2018. An analysis of categorical distributional reinforcement learning. In Proceedings of the International Conference on Artificial Intelligence and Statistics.
J. N. Tsitsiklis and B. Van Roy, 1997. An analysis of temporal-difference learning with function approximation.In Proceedings of the IEEE Transactions on Automatic Control.

Response to Reviewer 2

Comment 2.1

Summary. This manuscript introduces a "distributional deterministic RL" (DDRL) framework that combines distributional reinforcement learning, where agents learn full reward distributions, with deterministic learning dynamics based on strategy averaging. Optimism and pessimism are implemented through distortions of the learned value distribution, and the model is evaluated across three canonical contexts: explorationexploitation, social coordination, and intertemporal risky choice. In each case, the authors reproduce qualitative effects previously associated with optimism in related models.

The modeling framework is elegant and technically well presented. I particularly appreciate the care devoted to the methodological exposition and the accessibility of the code, which makes the model transparent and potentially useful as a tool for the reinforcement learning community.

At the same time, I have substantial concerns regarding the claimed scope and positioning of the contribution. The introduction is long and at times difficult to follow, and the central objective of the paper remains somewhat unclear. More importantly, several claims about the added value of DDRL in the study of heuristics and collective behavior appear stronger than what is directly supported by the analyses. In particular, the added value of this model relative to prior work is not clearly established. In its current form, the conceptual advance of this manuscript over the existing literature remains somewhat unclear. Below, I outline major points that, in my view, need to be addressed to clarify the paper's novelty, delimit its claims, and strengthen its relevance for cognitive scientists.

Response. We thank the reviewer for the constructive assessment and for the positive reception

of the technical framework. We have addressed each comment in detail below. In brief, we have completely revised and tightened the Introduction section, added empirical connections for each of the three task domains, and clarified the contribution relative to prior work.

Comment 2.2

Introduction/Lines 22-23: The statement that "an overarching explanation for [behavioral biases'] origin or adaptive character is lacking" feels somewhat too broad. While this may hold in a general sense, important explanations have been proposed for many specific biases (e.g., Gershman et al., 2015; Summerfield & Parpart, 2022; Shenhav et al., 2017), including within the heuristics tradition mentioned later in the manuscript. As written, the sentence may give the impression that the present study aims to provide a unifying account of all behavioral biases, which seems to extend beyond its actual scope. Clarifying this point would help position the contribution more precisely.

Response. We agree. The claim that an overarching explanation for behavioral biases is lacking is too broad — important explanations have been proposed for many specific biases, including the work cited by the reviewer (Gershman et al., 2015; Summerfield & Parpart, 2022; Shenhav et al., 2017). We have rewritten the opening paragraph of the Introduction. The revised version does not assert a universal explanatory gap; instead, it frames the paper's aim precisely: developing a framework that is analytically tractable, biologically grounded, and applied to the optimism heuristic across multiple task domains.

Comment 2.3

Introduction/Lines 37-38: The claim that "it is unclear why heuristics are so pervasive among humans and animals, and which brain mechanisms support them" overlooks a substantial body of existing work addressing these questions. Including relevant prior studies would strengthen the introduction and avoid suggesting that this issue has not yet been explored.

Response. We agree that a substantial body of work addresses the neural and cognitive basis of heuristics, and the original sentence overlooked it. The claim has been removed in the revised Introduction. The paper does not set out to explain why heuristics are pervasive; its contribution is a mechanistic computational framework for one well-studied heuristic — optimism — across multiple task domains.

Comment 2.4

Introduction/Lines 66-67: The statement that existing models treat optimism and pessimism as ad hoc parameters would benefit from further clarification, in order to clearly distinguish DDRL from them. As currently phrased, the sentence suggests that contrary to existing models, DDRL avoids ad hoc parameterization altogether, whereas optimism/pessimism appears to be implemented through a very specific distortion of the value distribution.

Response. We concede the point. DDRL also uses a parameter (w_U) to implement optimism, so the original sentence — implying that prior models were uniquely ad hoc — was misleading. We have revised it to clarify the actual distinction: prior models treat optimism as a *phenomenological* hyperparameter (a learning-rate asymmetry tuned to improve performance), whereas DDRL grounds it *mechanistically* in a distortion of the learned return distribution, connecting the optimism level to a

representational quantity with a neural counterpart. The distinction is not the absence of parameters, but the substrate they operate on.

> Comment 2.5
>
> **Introduction**/Lines 67-68: The statement that previous models "cannot explain why specific levels of optimism or pessimism are helpful in a given situation" seems overstated. In RL, the impact of a feature can typically be quantified by comparing cumulative reward with and without that feature.

Response. We agree. Whether specific optimism levels are beneficial can indeed be quantified in prior RL models by comparing cumulative reward across parameter settings; the original sentence overstated the gap. We have removed this claim. The revised Introduction reframes the contribution as mechanistic: DDRL derives optimism from a biologically motivated representational choice rather than treating the optimism level as a free parameter to be searched over.

> Comment 2.6
>
> **Introduction**/Lines 69-71: The claim that distributional RL uniquely provides the "representational richness" required to meaningfully implement and reason about optimism/pessimism is not sufficiently justified. It is unclear why previously cited approaches would not allow meaningful implementation or analysis of these constructs.

Response. We agree the claim was not sufficiently justified. We have removed it. The revised Introduction makes the narrower, supportable claim that DistRL provides the mechanistic substrate for grounding optimism in the structure of learned return distributions, with neural signatures identified in the dopaminergic system (Dabney et al., 2020).

> Comment 2.7
>
> **Introduction**/Lines 75-76: The sentence "doing so allows for improved treatment of uncertainty" is potentially misleading. It seems that the representation of the full return distribution that enables richer treatment of uncertainty, rather than the distortion of that distribution per se.

Response. The reviewer is correct. It is the full return distribution that enables richer treatment of uncertainty; the distortion of that distribution implements optimism, not the richer uncertainty representation. The sentence has been removed. The revised paragraph presents the distributional representation and the distortion-based optimism as distinct contributions, each with its own justification.

> Comment 2.8
>
> **Introduction**/Lines 78-81: The claim that the methodology allows agents to differentiate between environmental stochasticity and uncertainty due to insufficient exploration requires substantially more explanation. It is not clear how this distinction is implemented in the model. Moreover, the agents in the present study appear to have fixed levels of optimism/pessimism and thus cannot decide whether to be optimistic or pessimistic depending on the context.

Response. We agree. The claim that the methodology "enables the agent to differentiate between

uncertainty stemming from environmental stochasticity and that due to insufficient exploration" referred to the broader DistRL literature, where, in principle, the variance of the return distribution can encode both types. However, this observation does not hold for our DDRL framework. DDRL operates under deterministic dynamics in which stochastic noise is integrated out, so the learned distribution does not track epistemic uncertainty in this sense. Hence, we removed this claim in the revised Introduction to avoid confusion.

Regarding fixed optimism levels: this is a deliberate scope choice. DDRL studies the *consequences* of a given optimism level — characterising the attractor structure, fixed-point stability, and bifurcations as a function of w_U — rather than modelling how agents would adaptively select their optimism in response to context. Context-dependent, adaptive optimism remains an interesting avenue for future work.

> Comment 2.9
>
> **Introduction**/Lines 84-86: The statement that existing approaches have mostly been used in specific contexts (e.g., risky or collective choice) suggests that the present study goes beyond this limitation. However, the current manuscript also evaluates the model in specific contexts (albeit several of them). This distinction should be framed more accurately.

Response. We agree that the difference to prior work can be made more precise: DDRL also evaluates the framework in specific task contexts, three of them. Many prior works put their focus on one specific context. For example, asymmetric RL in cognitive science focuses on the stateless bandit choice task (Cazé and van der Meer, 2013). Lenient learning in multi-agent RL focuses on normal-form games (Panait et al., 2006). Distributional RL in machine learning focuses on single-agent Markov decision processes (Bellemare et al., 2017). The original framing implied that prior work was principally more context-bound than our own, which was unfair. We have revised the relevant passage to clarify this point: DDRL applies a single underlying framework to multiple task contexts, whereas many prior works focus on a single context.

> Comment 2.10
>
> **Introduction**/Lines 94-95: The claim that the field lacks a "unified, biologically plausible framework for modeling heuristics in RL" again suggests a level of generality not reflected in the paper. The present study focuses specifically on optimism and pessimism, not on heuristics more broadly.

Response. We agree. The paper addresses optimism and pessimism specifically, not heuristics in general. The Introduction has been updated accordingly: the contribution is described as a biologically grounded, analytically tractable framework for studying the optimism heuristic across task domains, not a unified theory of heuristics broadly.

> Comment 2.11
>
> **Introduction**/Lines 96-97: The phrase "combining multi-agent reinforcement learning with complexity science and evolutionary dynamics" is unclear. It would be helpful to specify how "complexity" science is applied to the model in practice, and where evolutionary dynamics enter the analysis.

Response. We agree the phrase was unclear. "Complexity science" was not operationalized in the paper, and "evolutionary dynamics" referred loosely to the game-theoretic formalism underlying DetRL rather than to evolutionary biology per se. We have removed this phrasing. The revised Introduction describes the evolutionary game theory analogy specifically and in context: deterministic RL dynamics carry a formalism analogous to evolutionary game theory, enabling exact characterization of fixed points and bifurcations of collective learning.

Comment 2.12

Introduction/Lines 104-106: The manuscript repeatedly refers to "collective behavior." However, in the current implementation, agents interact only indirectly through reward contingencies and do not model or predict each other's behavior. There is no explicit social inference or communication. Referring to this as "collective behavior" may therefore be misleading and should be reconsidered or more carefully defined.

Response. We appreciate this observation. We have added an explicit definition of collective behavior in the Introduction at its first appearance: "collective behavior — coordinated outcomes arising from individual learning in a shared environment, without requiring explicit communication between agents." This makes clear that the multi-agent interactions in DDRL are mediated entirely by shared reward structure, and that we use "collective behavior" in this broad sense — consistent with its use in evolutionary game theory and complex systems — throughout the paper.

Comment 2.13

Introduction/Line 128-129: The manuscript mentions that the deterministic aspect of the model can help understand the role of noise in learning, yet this issue is not revisited, and the impact of noise is not systematically explored.

Response. We agree. The claim that the deterministic model helps understand the role of noise in learning was not revisited in the Results or Discussion and has been removed from the Introduction. The relationship between deterministic fixed points and the stationary distribution of the corresponding stochastic system is noted in Section 2.4 (old: 2.1.4) as part of the methodological justification for deterministic dynamics, but is not presented as a result of this paper.

Comment 2.14

Claim that perseveration results from neural reward discretization In both the Abstract (lines 13–14) and the Discussion (lines 635–638), the authors suggest that perseveration may arise from low-level biological mechanisms such as distributed reward encoding, because this mechanism produces path dependence in the model's strategy. However, while path dependence is necessary for perseveration, it is not sufficient. The manuscript does not clearly demonstrate that the model produces perseverative behavior in the behavioral sense, nor does it disentangle path dependence from perseveration per se. This claim would benefit from either additional supporting analysis or more cautious phrasing.

Response. The reviewer is correct on both counts, and we thank them for the precise diagnosis. The original text conflated two distinct phenomena, and path dependence alone is indeed insufficient for perseveration in the behavioral sense.

Distinguishing choice hysteresis from perseveration. We have revised the Discussion to draw an explicit distinction. Choice hysteresis — path dependence in strategy space, where the attractor an agent reaches depends on initial conditions — is a property of the model's phase structure. Stuck-in-set perseveration (Sandson and Albert, 1984; Banavar and Bornstein, 2024), by contrast, refers to the deployment of a previously appropriate strategy that is no longer goal-congruent. Being locked into one of two attractors is not perseveration if that attractor still yields higher return; it becomes perseveration only when the agent is locked into the *lower*-valued attractor by its history.

A hierarchy of path-dependent phenomena. DDRL predicts a three-level hierarchy, all traceable to reward discretization. (1) In the bistable region, when both attractors have positive return ordering consistent with their stability, the model produces choice hysteresis but not perseveration. (2) When parameters cross the value-ordering boundary (thick black line, Fig. 4F) while remaining in the bistable region, an agent locked into the lower-valued attractor by its history is in a goal-incongruent state — an escapable form of stuck-in-set perseveration, because stochastic fluctuations in a realistic learning system could in principle drive a transition to the preferred basin. (3) In the individual dilemma regime (monostable, but with the stable strategy yielding lower return than the unstable one), no noise-induced correction is possible: the better strategy is no longer a fixed point. This constitutes *inescapable* stuck-in-set perseveration.

Hypothesis status and phrasing. We agree with the reviewer that the manuscript should not present this as a demonstrated behavioral result. That was never our intention. The individual dilemma provides a mechanistic account — a prediction — of inescapable perseveration, consistent with accounts that trace perseverative behavior to constraints on policy representation (Gershman, 2020; Banavar and Bornstein, 2024). Empirical demonstration would require a reversal-learning or outcome-omission paradigm in which the optimal strategy changes mid-task. We have revised the relevant passages in the Abstract and Discussion to reflect this hypothesis status, and have added the three-level hierarchy to the Discussion with appropriate hedging. The new citations (Sandson and Albert, 1984; Banavar and Bornstein, 2024; Gershman, 2020) ground the behavioral vocabulary.

Comment 2.15

Comparison with behavior The manuscript reproduces three empirical findings, previously captured by different models in distinct contexts, within a single framework. However, if the goal is to bridge machine learning and experimental psychology, it is essential to explain how the predicted qualitative effects are observed in human behavior (and not only in previous RL models).
Ideally, the authors could go further and fit the DDRL model to human behavioral data to demonstrate its empirical adequacy. At minimum, the manuscript should explicitly connect the simulated patterns to established behavioral findings. Without such comparison, it is difficult to assess the model's relevance for cognitive science.

Response. We thank the reviewer for the suggestion to strengthen the connection of our framework to empirical findings. We have added empirical connections to each of the three Results subsections, and we address the model-fitting question separately.

Two-armed bandit under resource variability (Section 3.2 (old: 3.1)). The model reproduces the computational finding that optimism aids performance in poor environments and pessimism in rich ones (Cazé and van der Meer, 2013). We have added a passage noting that in humans, asymmetric sensitivity to positive and negative prediction errors is well documented (Niv et al., 2012; Frank et al., 2004, 2007; Palminteri et al., 2017) and reflects variation in dopaminergic signaling. Interestingly,

and in contrast to Cazé and van der Meer (2013)'s original proposal, human learners tend toward a stable positivity bias regardless of environment statistics (Gershman, 2015), consistent with fixed rather than context-adaptive optimism weights — the assumption made in DDRL.

Social coordination (stag-hunt, Section 3.3 (old: 3.2)). We have added a passage citing human laboratory experiments. Under time pressure — which favors intuitive, optimistic social expectations — participants cooperate more in stag-hunt tasks (Belloc et al., 2019). Shared optimistic beliefs about partner trustworthiness increase cooperation by up to 40 percentage points (Jansson and Eriksson, 2015), and optimism about partner behavior predicts payoff-dominant choices (Jagau, 2024).

Intertemporal risky choice (Section 3.4 (old: 3.3)). To our knowledge, no behavioral experiment has studied an environment combining repeated stochastic choice with state-dependent consequences and temporal recovery dynamics. We have added a passage to Section 3.4 stating this explicitly, noting that risk-taking and intertemporal discounting have been explored jointly in simplified choice tasks (Andreoni and Sprenger, 2012) but not in a multi-state repeated structure of this form. We frame the novel effects — bistable regimes and individual dilemmas — as DDRL predictions that await empirical test. We regard this as a strength: the framework generates concrete, novel predictions about behavior in sustainability-motivated choice environments that have not yet been experimentally studied.

Model fitting. Fitting DDRL to individual-subject trial-by-trial data would require adapting the deterministic dynamics framework to a stochastic likelihood model — a non-trivial extension that is beyond the scope of this paper. We have added a paragraph to the Discussion (Section 4 (old: 4), behavioral implications) flagging model fitting as an important avenue for future empirical work.

Comment 2.16

Comparison with existing models
Although several reinforcement learning approaches are discussed and partially compared to DDRL (Figures 2B, 2C and 4D, 4E), the results are not fully contextualized. In the intertemporal risky choice task, DDRL predicts quantitatively different patterns compared to a non-distributional agent. However, it remains unclear which pattern aligns better with human data, or which is theoretically more plausible.
The authors argues their model is biological plausibility because it relies on distributed value encoding across simple units. However, classical RL models could also be implemented in population codes that represent expected values rather than full distributions.; biological inspiration alone does not necessarily establish greater plausibility. A more explicit conceptual or empirical comparison with alternative models would therefore, in my opinion, considerably strengthen the manuscript.
If relevant behavioral datasets are available, comparing model fits would be particularly informative. If such comparisons have already been performed elsewhere, discussing them would be valuable.

Response. We address the reviewer's two points in turn.

Intertemporal risky choice: which pattern aligns with human data? As noted in our response to Comment 2.15, the intertemporal risky choice environment has no direct behavioral analog in the existing experimental literature. The qualitative difference between DDRL and non-distributional dynamics (Figs. 4D–E) is therefore a theoretical prediction rather than a validated result. In particular, the bistable regimes that emerge in DDRL but not in non-distributional dynamics await empirical test. We have added a passage to Section 3.4 (old: 3.3) making this explicit.

Population codes and biological plausibility. The reviewer correctly observes that distributed encoding per se does not distinguish DDRL from a population code for expected value — a classical RL agent could in principle also be implemented as a population of neurons tuned to different values of the mean. The biological motivation for DDRL is more specific: it rests on population-level *distributional* coding, in which different neurons encode different quantiles or moments of the return distribution rather than a shared mean. This coding scheme is empirically distinguishable. Dopamine neurons exhibit systematic diversity in prediction-error asymmetries — some learning faster from better-than-expected outcomes, others from worse-than-expected outcomes — spanning the full return distribution (Dabney et al., 2020). This reversal-point diversity across the neuronal population cannot be explained by a population code for the expected value. D1 and D2 striatal neurons have been shown to encode opposite tails of the return distribution through an opponent circuit (Lowet et al., 2025), and distributional coding has also been identified in prefrontal cortex (Muller et al., 2024). We have added a passage to the biological plausibility paragraph of Section 4 (old: 4) (Discussion) making this distinction explicit. We do not claim that DDRL is uniquely biologically plausible; rather, that its representational assumptions correspond to a specific, empirically supported neural coding scheme that goes beyond generic distributed computation.

Comment 2.17

Applications to complex collective decision-making
In the Discussion (lines 665-678), the authors suggest that the model could be applied to complex collective decision-making problems such as responses to climate change. While this is an interesting perspective, the mechanisms described appear to rely primarily on risk-seeking versus risk-averse tendencies, which are already standard components of many decision-making models. It would be helpful to clarify what unique contribution the optimism/pessimism mechanism provides in this broader context.

Response. The reviewer correctly identifies that the paragraph as written leans on the optimism→risk-seeking / pessimism→risk-averse framing, which is standard in many decision-making models. We have revised the paragraph to articulate three aspects of DDRL that go beyond that framing.

Analytically tractable stability landscape. DDRL's deterministic dynamics yield an analytically accessible stability landscape (analogous to Fig. 4F) in which the conditions for coordination, collapse, and bistability can be identified exactly as functions of optimism weight, discount factor, and return discretization. This goes beyond knowing the direction of a risk bias: it specifies *when* and *under what conditions* optimism aids or undermines cooperation.

Simultaneous coordination and intertemporal collapse risk. A climate tipping-point scenario combines both a coordination structure (as in our stag-hunt) and an intertemporal collapse risk (as in our risk-reward dilemma). These appear in our paper as separate tasks; in a real collective-action problem they often operate simultaneously. DDRL provides a natural framework for analyzing their interaction analytically in a stateful multi-agent setting.

Broader applicability. We close with a sentence widening the outlook to the general question of how neurocognitive heuristics shape collective behavior across complex social-ecological systems (Barfuss et al., 2025; Constantino et al., 2021; Schill et al., 2019).

We have revised the paragraph accordingly (see Discussion section).

Comment 2.18

Minor points
The choice to model a population of 29 neurons is quite unusual, is there any specific reason for that?

Response. $N = 29$ was chosen to balance accuracy and computational cost across environments. To demonstrate this explicitly, we have added a new analysis to the supplementary notebook *Discretization calibration*, sweeping N over a coarse grid $\{5, 9, 13, 17, 21, 25, 29, 35, 41, 51\}$ for each of the three environments with σ fixed at its per-environment calibrated value. The discretization error — the norm of the difference between the TD errors of a DDRL and an expectation-based agent across a sample of strategies — plateaus by approximately $N = 25$ in the stag-hunt and risk-reward dilemma; $N = 29$ lies at or beyond the saturation point in all three environments. Beyond $N \approx 30$, computation time grows superlinearly while accuracy no longer improves. We have added a sentence to Section 3.1.1 (old: 2.2.1) noting this.

References

James Andreoni and Charles Sprenger. Risk preferences are not time preferences. *American Economic Review*, 102(7):3357–3376, 2012. doi: 10.1257/aer.102.7.3357.

Nidhi V. Banavar and Aaron Bornstein. Multi-Plasticities: Distinguishing Context-Specific Habits from Complex Perseverations. In Youna Vandaele, editor, *Habits: Their Definition, Neurobiology, and Role in Addiction*, pages 87–106. Springer International Publishing, Cham, 2024. ISBN 978-3-031-55889-4. doi: 10.1007/978-3-031-55889-4_5.

Wolfram Barfuss. Dynamical systems as a level of cognitive analysis of multi-agent learning. *Neural Computing and Applications*, 34(3):1653–1671, February 2022. ISSN 1433-3058. doi: 10.1007/s00521-021-06117-0. URL https://doi.org/10.1007/s00521-021-06117-0.

Wolfram Barfuss and Janusz M Meylahn. Intrinsic fluctuations of reinforcement learning promote cooperation. *Scientific reports*, 13(1):1309, 2023.

Wolfram Barfuss, Jonathan F Donges, and Jürgen Kurths. Deterministic limit of temporal difference reinforcement learning for stochastic games. *Physical Review E*, 99(4):043305, 2019.

Wolfram Barfuss, Jessica Flack, Chaitanya S Gokhale, Lewis Hammond, Christian Hilbe, Edward Hughes, Joel Z Leibo, Tom Lenaerts, Naomi Leonard, Simon Levin, et al. Collective cooperative intelligence. *Proceedings of the National Academy of Sciences*, 122(25):e2319948121, 2025.

Marc G Bellemare, Will Dabney, and Rémi Munos. A distributional perspective on reinforcement learning. In *International conference on machine learning*, pages 449–458. PMLR, 2017.

Marc G. Bellemare, Will Dabney, and Mark Rowland. *Distributional Reinforcement Learning*. MIT Press, May 2023.

Marianna Belloc, Ennio Bilancini, Leonardo Boncinelli, and Simone D'Alessandro. Intuition and deliberation in the stag hunt game. *Scientific Reports*, 9:14833, 2019. doi: 10.1038/s41598-019-50556-8.

Tilman Börgers and Rajiv Sarin. Learning Through Reinforcement and Replicator Dynamics. *Journal of Economic Theory*, 77(1):1–14, November 1997. ISSN 00220531. doi: 10.1006/jeth.1997.2319.

Vivek S. Borkar. *Stochastic Approximation: A Dynamical Systems Viewpoint*. Springer, 2008. ISBN 978-93-86279-38-5. doi: 10.1007/978-93-86279-38-5.

Romain D Cazé and Matthijs AA van der Meer. Adaptive properties of differential learning rates for positive and negative outcomes. *Biological cybernetics*, 107(6):711–719, 2013.

S. M. Constantino, M. Schlüter, E. U. Weber, and N. Wijermans. Cognition and behavior in context: A framework and theories to explain natural resource use decisions in social-ecological systems. *Sustainability Science*, 16(5):1651–1671, September 2021. ISSN 1862-4057. doi: 10.1007/s11625-021-00989-w.

Will Dabney, Georg Ostrovski, David Silver, and Remi Munos. Implicit Quantile Networks for Distributional Reinforcement Learning. In *Proceedings of the 35th International Conference on Machine Learning*, pages 1096–1105. PMLR, July 2018a.

Will Dabney, Mark Rowland, Marc Bellemare, and Rémi Munos. Distributional reinforcement learning with quantile regression. In *Proceedings of the AAAI conference on artificial intelligence*, volume 32, 2018b.

Will Dabney, Zeb Kurth-Nelson, Naoshige Uchida, Clara Kwon Starkweather, Demis Hassabis, Rémi Munos, and Matthew Botvinick. A distributional code for value in dopamine-based reinforcement learning. *Nature*, 577(7792):671–675, 2020.

Jesse Farebrother, Jordi Orbay, Quan Vuong, Adrien Ali Taiga, Yevgen Chebotar, Ted Xiao, Alex Irpan, Sergey Levine, Pablo Samuel Castro, Aleksandra Faust, Aviral Kumar, and Rishabh Agarwal. Stop Regressing: Training Value Functions via Classification for Scalable Deep RL. In *Proceedings of the 41st International Conference on Machine Learning*, pages 13049–13071. PMLR, July 2024.

Simone Ferrari-Toniolo and Wolfram Schultz. Reliable population code for subjective economic value from heterogeneous neuronal signals in primate orbitofrontal cortex. *Neuron*, 111(22):3683–3696.e7, November 2023. ISSN 0896-6273. doi: 10.1016/j.neuron.2023.08.009.

Michael J Frank, Lauren C Seeberger, and Randall C O'Reilly. By carrot or by stick: cognitive reinforcement learning in parkinsonism. *Science*, 306(5703):1940–1943, 2004. doi: 10.1126/science.1102941.

Michael J Frank, Ahmed A Moustafa, Heather M Haughey, Tim Curran, and Kent E Hutchison. Genetic triple dissociation reveals multiple roles for dopamine in reinforcement learning. *Proceedings of the National Academy of Sciences*, 104(41):16311–16316, 2007. doi: 10.1073/pnas.0706111104.

Samuel J Gershman. Do learning rates adapt to the distribution of rewards? *Psychonomic Bulletin & Review*, 22(5):1320–1327, 2015. doi: 10.3758/s13423-014-0790-3.

Samuel J. Gershman. Origin of perseveration in the trade-off between reward and complexity. *Cognition*, 204:104394, November 2020. ISSN 0010-0277. doi: 10.1016/j.cognition.2020.104394.

R. J. Herrnstein. Relative and absolute strength of response as a function of frequency of reinforcement. *Journal of the Experimental Analysis of Behavior*, 4(3):267–272, July 1961. ISSN 0022-5002. doi: 10.1901/jeab.1961.4-267.

Josef Hofbauer and Karl Sigmund. *Evolutionary Games and Population Dynamics*. Cambridge University Press, 1 edition, May 1998. ISBN 978-0-521-62365-0 978-0-521-62570-8 978-1-139-17317-9. doi: 10.1017/CBO9781139173179.

Ehsan Imani and Martha White. Improving Regression Performance with Distributional Losses. In *Proceedings of the 35th International Conference on Machine Learning*, pages 2157–2166. PMLR, July 2018.

Stephan Jagau. To catch a stag: Identifying payoff- and risk-dominance effects in coordination games. *Games and Economic Behavior*, 147:429–448, 2024. doi: 10.1016/j.geb.2024.08.011.

Fredrik Jansson and Kimmo Eriksson. Cooperation and shared beliefs about trust in the assurance game. *PLoS ONE*, 10(12):e0144191, 2015. doi: 10.1371/journal.pone.0144191.

Edwin T Jaynes and G. Larry Bretthorst. *Probability Theory: The Logic of Science*. Cambridge University Press, Cambridge, 2003. ISBN 978-0-511-06589-7.

Adam S. Lowet, Qiao Zheng, Melissa Meng, Sara Matias, Jan Drugowitsch, and Naoshige Uchida. An opponent striatal circuit for distributional reinforcement learning. *Nature*, 639(8055):717–726, 2025. ISSN 1476-4687. doi: 10.1038/s41586-024-08488-5.

Timothy H. Muller, James L. Butler, Sebastijan Veselic, Bruno Miranda, Joni D. Wallis, Peter Dayan, Timothy E. J. Behrens, Zeb Kurth-Nelson, and Steven W. Kennerley. Distributional reinforcement learning in prefrontal cortex. *Nature Neuroscience*, 27(3):403–408, 2024. ISSN 1546-1726. doi: 10.1038/s41593-023-01535-w.

Yael Niv, Jeffrey A Edlund, Peter Dayan, and John P O'Doherty. Neural prediction errors reveal a risk-sensitive reinforcement-learning process in the human brain. *Journal of Neuroscience*, 32(2): 551–562, 2012. doi: 10.1523/JNEUROSCI.5498-10.2012.

Stefano Palminteri, Germain Lefebvre, Emma J Kilford, and Sarah-Jayne Blakemore. Confirmation bias in human reinforcement learning: Evidence from counterfactual feedback processing. *PLoS computational biology*, 13(8):e1005684, 2017.

Liviu Panait, Keith Sullivan, and Sean Luke. Lenient learners in cooperative multiagent systems. In *Proceedings of the fifth international joint conference on Autonomous agents and multiagent systems*, pages 801–803, 2006.

Mark Rowland, Robert Dadashi, Saurabh Kumar, Rémi Munos, Marc G Bellemare, and Will Dabney. Statistics and samples in distributional reinforcement learning. In *International Conference on Machine Learning*, pages 5528–5536. PMLR, 2019.

Mark Rowland, Shayegan Omidshafiei, Daniel Hennes, Will Dabney, Andrew Jaegle, Paul Muller, Julien Pérolat, and Karl Tuyls. Temporal difference and return optimism in cooperative multi-agent reinforcement learning. In *AAMAS ALA Workshop*, 2021.

Jennifer Sandson and Martin L. Albert. Varieties of perseveration. *Neuropsychologia*, 22(6):715–732, January 1984. ISSN 0028-3932. doi: 10.1016/0028-3932(84)90098-8.

Caroline Schill, John M Anderies, Therese Lindahl, Carl Folke, Stephen Polasky, Juan Camilo Cárdenas, Anne-Sophie Crépin, Marco A Janssen, Jon Norberg, and Maja Schlüter. A more dynamic understanding of human behaviour for the anthropocene. *Nature Sustainability*, 2(12): 1075–1082, 2019.

Alireza Soltani and Xiao-Jing Wang. A Biophysically Based Neural Model of Matching Law Behavior: Melioration by Stochastic Synapses. *Journal of Neuroscience*, 26(14):3731–3744, April 2006. ISSN 0270-6474, 1529-2401. doi: 10.1523/JNEUROSCI.5159-05.2006.

John Tsitsiklis and Benjamin Van Roy. Analysis of Temporal-Diffference Learning with Function Approximation. In *Advances in Neural Information Processing Systems*, volume 9. MIT Press, 1996.

Esther van Duuren, Jan Lankelma, and Cyriel M. A. Pennartz. Population Coding of Reward Magnitude in the Orbitofrontal Cortex of the Rat. *Journal of Neuroscience*, 28(34):8590–8603, August 2008. ISSN 0270-6474, 1529-2401. doi: 10.1523/JNEUROSCI.5549-07.2008.

David H. Wolpert, Michael Harré, Eckehard Olbrich, Nils Bertschinger, and Jürgen Jost. Hysteresis effects of changing the parameters of noncooperative games. *Physical Review E*, 85(3):036102, March 2012. ISSN 1539-3755, 1550-2376. doi: 10.1103/PhysRevE.85.036102.
