# Supplementary material for: Deterministic dynamics of distributional multi-agent reinforcement learning
Source: PLoS Comput Biol. 2026 Sep 23;22(9):e1014723. doi: 10.1371/journal.pcbi.1014723 (PMC13630324; doi:10.1371/journal.pcbi.1014723)
Supplement: Supplementary file 4 [file pcbi.1014723.s005.pdf]

PLOS Computational Biology

Jun.-Prof. Dr. Wolfram Barfuss
Argelander Professor for
Integrated System Modelling
for Sustainability Transitions
University of Bonn

Phone: +49-228-73-4476
Email: wbarfuss@uni-bonn.de
Web: https://wbarfuss.github.io

July 29, 2026

PCOMPBIOL-D-25-02733R1
Deterministic dynamics of distributional multi-agent reinforcement learning

> Dear Dr. Barfuss,
> Thank you for submitting your manuscript to PLOS Computational Biology. After careful consideration, we feel that it has merit but does not fully meet PLOS Computational Biology's publication criteria as it currently stands. Therefore, we invite you to submit a revised version of the manuscript that addresses the points raised during the review process.
> While the reviewers were happy with the overall progress of the paper, reviewer 1 raised some valid concerns which require minor corrections.
> [...]
> We look forward to receiving your revised manuscript.
> Kind regards,
> Ulrik R. Beierholm, Academic Editor, PLOS Computational Biology
> Andrea E. Martin, Section Editor, PLOS Computational Biology

Dear Dr. Beierholm and Dr. Martin,

Thank you for the opportunity to revise our manuscript, and to Reviewer 1 for a careful second reading. The main changes in this round are:

1. We corrected the maximum-entropy characterization of the Boltzmann policy and dropped the "least arbitrary choice" wording (Comment 1.2).
2. We removed the incorrect claim that expectile-based return representations have no neural analogue (Comment 1.3).
3. We qualified the claim relating the stochastic system's stationary distribution to the deterministic fixed points (Comment 1.4).
4. We made the distinction between reward and return discretization explicit throughout: behavior is governed by the discretized return distribution, while reward discretization is the mathematical step that makes that distribution computable in closed form (Comments 1.5 and 1.10).
5. We clarified the aims of the framework — DDRL is a method of analysis, not an algorithmic proposal — and defined what "validate" means in this context (Comment 1.8).
6. We disambiguated the two semantics related to the time index t and the scope of the strategy update (Comment 1.9).
7. We fixed the typographical and notational issues the reviewer listed (Comment 1.11).

Detailed responses to each comment of the reviewers are provided below.

We hope that the revised manuscript now meets the journal's publication criteria, and we look forward to hearing from you.

With kind regards,

Wolfram Barfuss, on behalf of all authors

Response to Reviewer 1

Comment 1.1

I thank the authors for their detailed response and updates in light of the reviews. This review first responds to the authors' letter (unless otherwise noted, I'm happy that the revisions made by the authors have dealt with the points raised in my initial review), and then provides further comment on the revised manuscript as a whole.
Comment 1.2
Thank you for the updates here. I have several comments in response; these are all reasonably minor/low-level technical comments, save for the point about reward vs. return distribution discretization, which I think is important to the overall message of the manuscript.

Response. We thank the reviewer for the careful second reading and for the constructive comments. We respond to each of the reviewer's points in turn below.

Comment 1.2

Boltzmann policies. I think the connection with the maximum-entropy principle is more subtle than the new paragraph implies. Specifically, I don't think the sentence "Among all distributions over actions consistent with current Q-value beliefs, it maximizes entropy" is accurate (or the term "consistent" should be explained in more detail). I think the correct claim is that the Boltzmann distribution can be derived as the distribution over actions that maximizes expected return (according to the Q-function) subject to a constraint on entropy (or alternatively, maximizes entropy subject to a constraint on the expected return (according to the Q-function). It may be that I am misinterpreting the authors' claim here, so potentially further explanation of the claim would also suffice. I am also unsure about the claim that this is the "least arbitrary choice", since it still requires the selection of the coefficient beta^i.

Response. We agree with the reviewer that our formulations were missleading. We corrected the claim to the constrained-optimization statement: the Boltzmann distribution maximises entropy subject to a constraint on the expected Q-value, equivalently maximises the expected Q-value subject to a constraint on entropy, with β^i the Lagrange multiplier coupling the two objectives. We also dropped "least arbitrary choice," since it does not hold once β^i is a free parameter.

Comment 1.3

Return-distribution representation. I'm not sure about the argument that quantile-/expectile-based approximations have no clear neural analogue. Dabney et al. (2020) argue for an expectile-based representation of return distributions in modelling certain neural phenomena,

and in some ways these representations have more in common with the non-distributional setting (in which an expected value is parameterized directly), rather than in the categorical setting parametrizing probabilities, rather than outcomes. To be clear, I think the choice to focus on the categorical representation in this paper is a reasonable one, but I don't agree with the above argument for avoiding quantile/expectile-based approaches; it may be that I'm missing something, however. The authors could adapt this specific claim, or provide more reasoning for it.

Response. We thank the reviewer for pointing this out. Dabney et al. (2020) indeed provide direct neural evidence for expectile-based coding of value distributions. So the claim that quantile/expectile-based representations have "no clear neural analogue" was incorrect and an oversight on our part. We removed it in the revised version of the manuscript.

Comment 1.4

Deterministic dynamics. The additional material here helps justify the choice of dynamics, and I found the mentioned connections to replicator systems interesting. I disagree with the sentence "the stationary distribution of the stochastic system concentrates around the stable fixed points of the deterministic part and spends negligible time near unstable ones". I think qualitatively this expresses the right intuitions, but when exactly this is true (versus when stochasticity is such that convergence is prevented, or when the dynamical system may enter a limit cycle rather than reaching the neighborhood of a stationary point) are subtle questions in general. Particularly given that the ODE method is mentioned in the same paragraph, which aims to carefully analyse these questions mathematically.

Response. We agree the claim was overstated as such. We qualified it in the revised version of the manuscript. Intuitively, the concentration result holds provided the noise is sufficiently weak and the deterministic dynamics have isolated stable fixed points rather than more complex attractors. We also made explicit that these are precisely the conditions the ODE method for stochastic approximation (already cited in this paragraph) characterizes rigorously, although from a different perspective and different goals.

Comment 1.5

Comment 1.4
Reward discretization vs. return discretization. With the authors' scaling of rewards by (1-gamma), I agree that reward and return ranges coincide in this work. However, there remains an algorithmic difference between projecting rewards and projecting returns. The authors state in their response to Comment 1.4 that they have now aligned the manuscript with the convention that returns, not rewards, are discretized, but I think there are still numerous references to "reward discretization" in the text (Line 11 of abstract, and in the main paper, e.g. Lines 54 & 227), and I believe Section 3.1.1 still describes the model implementation as discretizing rewards (in addition to returns; see, e.g., Eqn (14)). Unless I have missed something, this seems like a key issue for future revision.

Response. We thank the reviewer for bringing up this point up again. In the last revision, we wanted to retain the formulation of "reward neurons" where the biological framing is primary. This framing carried over too far into reward discretization.

Our framework is consitent with standard distributional RL in that discretized return distributions are the object from which actions are selected. We start, however, already with discretized rewards, as these are required to compute the return distribution in closed form (see Sect. 3.1.4), which is necessary to formulate the deterministic dynamics. So, our reward discretization can be seen as a mathematical step towards the discretized return distribution, which determines the agent's behavior. We clarified this point in the revised version of the manuscript and also emphasize our findings being due to return discretization instead of reward discretization where appropriate. We retain, however, the concept of reward neurons and refraind from calling them return neurons. In this context, return carries the same units as rewards and, thus can also be expressed in terms of reward neurons.

> Comment 1.6
>
> Gaussian smoothing + nearest neighbor projection. Thanks for the additional explanations, I agree with the authors' summary here, and the cited connection with Farebrother et al. and Imani & White in the revision.

Response. Thank you.

General comments in light of the new revision

> Comment 1.7
>
> Overall, the revisions here mostly resolve the technical queries raised in my first review, though a few queries remain, as discussed above, and including the discrepancy between reward/return discretization, summarized below. I remain of the opinion that this work is interesting, complements existing distributional RL literature in the domains of machine learning and neuroscience, and is likely to lead to further investigation in this area. I think there are still some notational/technical issues to address, as described above, and discuss several broad areas where I think there is room for improvement below.

Response. We thank the reviewer for this positive assessment. The remaining notational and technical points are addressed in our responses above and below, and the reward-versus-return discretization concern in our responses to Comments 1.5 and 1.10.

> Comment 1.8
>
> *Framing of contributions.*
> In reading the comments addressed in the other review, I'd agree that further explanation and emphasis of the key aims of the paper would be helpful. For example, at the end of the revised introduction, it is stated that "we validate the resulting learning dynamics"; it could help to more clearly state what the goals of the modeled dynamics are, and what it means to validate them. My understanding is the goal of the dynamics/model is not to have a realistic model of a multi-agent learning system (since this would typically involve stochasticity in agents' actions, imperfectly estimated value functions, etc.), but to have a simpler model that nevertheless provides mathematical predictions of phenomena in multi-agent systems that can be validated against observation. Additionally, my understanding is that the proposals here are not algorithmic proposals for agents implementing reinforcement learning (for example, it is often

not possible for an agent to compute the expected quantities that feature in these dynamics), but instead are proposals for methods of analysis.

Response. We agree and revised the end of the Introduction accordingly. We now state explicitly that DDRL's deterministic dynamics describe the expected trajectory of a learning agent, obtained by averaging out the noise of the underlying stochastic learning update over strategies, transitions, and rewards, and that DDRL is therefore a method of analysis rather than an algorithmic proposal. We also define what "validate" means in this context: testing whether DDRL's predictions (fixed points, their stability, and bifurcations) reproduce established behavioral findings, not asking whether agents implement this exact computation.

Comment 1.9

Clarity of equations/notations.
I thank the authors for updating these sections in light of my technical comments in the initial review. There are still some concerns around clarity:

- The index t appears to be used with two different semantics. In e.g. Eqn (2), it indexes both states/actions, and estimated quantities like Q-functionsm implying that it refers to time within an MDP episode. However, around Eqn (10), it is not clear whether this is still the case (the states/actions are not t-indexed here), and the indexing of the policy X_t implies this may no longer be time steps in the game, but instead discrete time steps in the discretiszd dynamics.
- Similarly, it is unclear at Eqn (11) whether this means a single action a^i at a single state s is taken at time t, and so Eqn (11) applies to update X_t only for these coordinates, or if the update is intended to apply to all state-actions pairs.

Response. We would view it as the index t retains its meaning as time step thoughout the manuscript, However, there are two different semantics regarding the kind of learning update: the standard algorithmic update (Eqs. 3 and 4) is a single-trajectory update, while the strategy-averaged update (Eq. 11 onward) is an update of the entire joint strategy $\mathbf{X}_t$ at once. In the revised version of the manuscript, we added an explicit remark at the point where the manuscript switches from single-trajectory to strategy-averaged quantities (i.e., where Eq. 11 is introduced): in Eqs. 3 and 4, t indexes the single state s_t and action a_t^i sampled at that step, and only the visited coordinate's estimate is updated; from Eq. 11 onward, s, a^i range over every state-action pair, all updated at each step t together, since the joint strategy $\mathbf{X}_t$ — not a sampled state-action pair — is the sole variable carrying the time index. We also added a corresponding clause directly at the strategy update equation (now Eq. 13) stating that it applies to every (s, a^i) pair at once, which resolves the reviewer's question about Eqs. 12 and 13's scope.

Comment 1.10

Discrepancy from standard distributional RL algorithms.
This concern was raised in the original review: namely, that there are certain choices in defining the distributional RL methods in the paper that differ from standard choices in the distributional RL literature, such as the discretization of rewards, rather than returns (the authors have responded to this query raised in the original review; see the response to Comment 1.4 above for

more technical details and remaining queries). I think currently, the discrepancy between reward discretization (the approach taken in this paper) and return discretization (the approach broadly taken in the distributional RL literature) is not sufficiently discussed; I think this difference isn't necessarily a problem per se, but this could perhaps be made more prominent in the text, since from what I understand this specific design decision is crucial to the predictions that emerge from the model, see e.g. Line 675: "Reward discretization thus generates a hierarchy of path-dependent phenomena–from choice hysteresis, to escapable perseveration", and so the conclusions reached in the paper may be somewhat specific to this non-standard choice of algorithmic specification.

Response. See our response to Comment 1.5 for the mechanism and the terminology convention we now use throughout. This response addresses the reviewer's remaining point about whether our conclusions are specific to a non-standard choice.

What is crucial to the results is that the return distribution has a discrete, finite support. This is established by the comparison between a non-distributional agent (continuous return spectrum, Fig. 4D) and a neutral-weight DDRL agent (discretized return distribution, Fig. 4E). Only the latter is bistable. That property is not a departure from standard categorical distributional RL; it is what defines it. What is specific to our implementation is *how* that discrete support is populated: Gaussian smoothing, applied first to the reward and then propagated through the Bellman recursion, rather than a mean-preserving (Cram'er) projection applied directly to the return, as in Bellemare et al. (2017). We already discuss this alternative in the Discussion (Limitations), including the fact that it would align the framework more closely with standard categorical distributional RL. In addition, we explicilty mention in the revised version of the Results and Discussion sections, that we use Gaussian smoothing as one component of our framework on which our results depend.

Comment 1.11

General minor typos etc.
There are several typos/notational points that could be addressed. A non-exhaustive list is given below:

1. Line 255: "[60, 61]and" is missing a space.
2. Line 63: "cartesian product" -> "Cartesian product"
3. alpha is overloaded as a step size and part of the optimism parametrization.
4. Eqn (15): sum_{ ^j} should be sum_{alpha^{-i}} I think.
5. Around Line 154, we have both r_t (the reward at time t), and r_n (the nth reward atom location).
6. Line 264: There is an unbound t on RHS of new "G =" equation.

Response. We thank the reviewer for this list and address each point in turn.

1. Fixed.
2. Fixed.
3. In fact, the symbol, α, refers to a learning rate throughout. In asymmetric-RL, α^+/α^- are used to denote the learning rates for positive and negative reward-prediction errors, respectively. The actual gap was that α^+/α^- were used in an equation (bias ratio $b = \alpha^+/\alpha^- = w_U$) well before that definition was spelled out in words, one subsection later. We fixed this issue in the revised version of the manuscript by stating the definition at that first symbolic use.

4. Fixed. The affected equation (then Eq 15) and three further instances of the same miss-notation, plus a related bold-X/bold-a notation slip now all match the established a^{-i} / unbold-X^{j,s,a^j} convention used elsewhere in the manuscript.
5. Fixed. We removed the restated gain function at that point and instead referenced the existing (now labeled) gain equation, Eq. 1, avoiding the confusing use of the reward indicies within this paragraph.
6. Fixed.

We also proofread the entire manuscript carefully. Beyond the specific items above, we corrected a small number of further typographical errors (e.g. a split word in a figure caption), a subject–verb agreement error, and several notational inconsistencies — most notably unifying the symbol for the natural numbers, the typesetting of `min`/`max` subscripts, and introducing the action-conditioned transition tensor $T_{\mathbf{X}}^{i,s,a^i,\acute{s}}$, which was previously used without an explicit definition. We also unified the terminology for the Gaussian discretization parameter σ, which had accumulated several names across the manuscript (e.g. “discretization parameter”, “discretization standard deviation”, “sensitivity”). It is now consistently termed the *smoothing width* and defined once as the standard deviation of the Gaussian kernel. We also standardized spelling and the formatting of figure, equation, and section references throughout. All corrections are visible in the tracked-changes version of the manuscript.
