# Supplementary material for: Deterministic dynamics of distributional multi-agent reinforcement learning
Source: PLoS Comput Biol. 2026 Sep 23;22(9):e1014723. doi: 10.1371/journal.pcbi.1014723 (PMC13630324; doi:10.1371/journal.pcbi.1014723)
Supplement: Peer review history

# Peer review: Decision Letter 0

**DOI:** 10.1371/journal.pcbi.1014723.r001

**Authors:** Ulrik Beierholm, Andrea E. Martin

3 Apr 2026

PCOMPBIOL-D-25-02733

Deterministic dynamics of distributional multi-agent reinforcement learning

PLOS Computational Biology

Dear Dr. Barfuss,

Thank you for submitting your manuscript to PLOS Computational Biology. After careful consideration, we feel that it has merit but does not fully meet PLOS Computational Biology's publication criteria as it currently stands. Therefore, we invite you to submit a revised version of the manuscript that addresses the points raised during the review process.

The paper was read by the editorial team, as well as by two reviewers who provided detailed comments. In general, there was an appreciation of the detailed modeling work, but the reviewers highlighted some deficiencies in the writing. Especially (but not exclusively) the description of the modeling framework (reviewer 1) and the positioning of the contribution relative to the literature (reviewer 2) would need to be better explained in a resubmission.

Please submit your revised manuscript by Jun 03 2026 11:59PM. If you will need more time than this to complete your revisions, please reply to this message or contact the journal office at ploscompbiol@plos.org. When you're ready to submit your revision, log on to https://www.editorialmanager.com/pcompbiol/ and select the 'Submissions Needing Revision' folder to locate your manuscript file.

Please include the following items when submitting your revised manuscript:

* A letter that responds to each point raised by the editor and reviewer(s). You should upload this letter as a separate file labeled 'Response to Reviewers'. This file does not need to include responses to formatting updates and technical items listed in the 'Journal Requirements' section below.

* A marked-up copy of your manuscript that highlights changes made to the original version. You should upload this as a separate file labeled 'Revised Manuscript with Track Changes'.

* An unmarked version of your revised paper without tracked changes. You should upload this as a separate file labeled 'Manuscript'.

If you would like to make changes to your financial disclosure, competing interests statement, or data availability statement, please make these updates within the submission form at the time of resubmission. Guidelines for resubmitting your figure files are available below the reviewer comments at the end of this letter

We look forward to receiving your revised manuscript.

Kind regards,

Ulrik R. Beierholm

Academic Editor

PLOS Computational Biology

Andrea E. Martin

Section Editor

PLOS Computational Biology

**Journal Requirements:**

1) We ask that a manuscript source file is provided at Revision. Please upload your manuscript file as a .doc, .docx, .rtf or .tex. If you are providing a .tex file, please upload it under the item type u2018LaTeX Source Fileu2019 and leave your .pdf version as the item type u2018Manuscriptu2019.

2) Please provide an Author Summary. This should appear in your manuscript between the Abstract (if applicable) and the Introduction, and should be 150-200 words long. The aim should be to make your findings accessible to a wide audience that includes both scientists and non-scientists. Sample summaries can be found on our website under Submission Guidelines:

https://journals.plos.org/ploscompbiol/s/submission-guidelines#loc-parts-of-a-submission

3) Please upload all main figures as separate Figure files in .tif or .eps format. For more information about how to convert and format your figure files please see our guidelines:

https://journals.plos.org/ploscompbiol/s/figures

**Reviewers' comments:**

Reviewer's Responses to Questions

**Comments to the Authors:**

**Please note here if the review is uploaded as an attachment.**

Reviewer #1: __Main claims of paper__

This paper studies multi-agent interactions from the perspective of stochastic games, a well-established approach in game theory. The paper is specifically concerned with the behavior of agents that interact and learn through the use of distributional reinforcement learning algorithms, and the use of such a framework in modeling and understanding heuristics in human decision-making.

From a methodological point of view, the primary focus of the paper is on a variant of the categorical approach to distributional reinforcement learning, with a specific choice of rule for inducing behavior based on optimistic/pessimistic summaries of learned distributions. The authors focus on a simplified model of the multi-agent interactions, in which discrete updates are made on the basis of expected behavior (rather than random updates that may arise from e.g. agents randomly sampling actions, or stochastic environments).

Interactions are simulated in three domains, and several connections are drawn between the methodological parameters, such as reward discretization and discount factor, and phenomena arising in human-decision making, such as perseveration, choice hysteresis, and "individual dilemma".

__High-level discussion__

The direction of building a framework to model aspects of human decision-making through distributional reinforcement learning is an interesting, novel one. In this regard, the work naturally complements prior work on distributional reinforcement learning in machine learning and neuroscience. I expect the emphasis here on studying the dynamical systems that arise from distributional learning algorithms in a variety of contexts to spur on future work in this area.

That said, I think the paper would benefit from expanded discussion of the framework that the authors introduce here. In particular, there are a few specific choices made (categorical approximations of distributions, use of deterministic dynamics, a specific form of optimism/pessimism), and it was not clear to me whether the authors are advocating for these choices over others (or just making one of many possible choices). For example, it would also be possible to investigate what happens when a quantile-based approximation of distributions is used, with a different form of optimism/pessimism, and simulating dynamics that take into account policy randomness. I think exhaustively examining other possibilities like this is beyond the scope of the paper, and studying the consequences of one possible set of choices is a nice contribution; I think though it would strengthen the paper to emphasize that these other choices exist, to what extent the authors expect the findings to translate to these other settings, and why the paper focuses on the choices made here.

The simulation findings in Sections 3.1/3.2 replicate earlier findings regarding benefits of optimism/pessimism depending on the reward structure of the environment. I found the findings in Section 3.3 regarding the interaction of discount factor and optimism levels, and resulting (bi)stability of policies interesting, and I expect there to be interest in investigating these phenomena further in future work.

There are several deviations in the methodology presented here from standard distributional RL practice. Some of these deviations (in particular, using discretization rather than Cramer projections in approximating reward/return distributions) seem important in several of the claims, so it would be useful to have further discussion of these choices in the paper. Further details are given below.

The authors have provided detailed explanations and code in the supplementary material that greatly aids understanding of the nuances of the simulation protocols, and should be highly useful to other researchers in building on this work.

__Main comments and concerns__

_Some lack of discussion of choices made_

As mentioned in the high-level discussion above, there are several methodological choices made in the paper that could benefit from further discussion; in brief, why are these specific choices made, and what might we expect to find if we make other choices? Some important examples are given below.

Eqn (6) commits to an exploration policy given by a Boltzmann distribution. However, there is no discussion of why this choice is made over e.g. epsilon-greedy exploration, or more involved approaches such as UCB-based exploration.

The work commits to a categorical representation of the return distribution. Other representations are available, such as quantile-based approaches (Dabney et al., 2018), or approaches that learn other properties of distributions, such as expectiles.

In Section 2.1.4, the paper describes analyzing learning dynamics with the noise in TD updates integrated out. Further discussion as to why this choice is made would be very beneficial; for example, presumably this approach makes simulating dynamics more straightforward and easier to replicate. On the other hand, it is not clear what might be lost by using this simplification of the dynamical system. An important case for this paper might be when both agents in a symmetric two-player game are initialized to the same strategy (as in Section 3.2). Under deterministic dynamics, their strategies remain coupled throughout the dynamics, but in real stochastic play, they will become uncoupled. Does this more general situation in the stochastic case change the conclusions of the paper? Perhaps a brief discussion/comparison to the "ODE method" in dynamical systems could be made (e.g. Borkar, 2008 as a general reference, or Tsitsiklis and Van Roy, 1997, for specific application to reinforcement learning); this technique also uses deterministic dynamics to analyse behavior of dynamical systems, and provides results that guarantee the same asymptotic behavior of stochastic dynamics and their deterministic counterpart, under certain conditions.

The use of Gaussian smoothing of returns in Section 2.2.1 is introduced without discussion of why this choice is made. This is not a default approach in distributional reinforcement learning, so it would be interesting to discuss why the choice was made here. There is however some precedent for this idea in distributional RL (see below) which could be cited.

Section 2.3 defines one way of obtaining optimistic/pessimistic value summaries of a distribution (although the definition is somewhat unclear; see below). It's unclear why this precise choice is made, how alternatives would affect the conclusions of the paper, and how this choice compares to earlier approaches in distributional RL (such as the use of expectiles in Rowland et al., 2021, or distortion risk measures in Dabney et al., 2018b).

In summary, there are a few particular methodological choices here (categorical distributions, particular types of optimistic/pessimistic distribution summaries) that I can imagine researchers in future expanding on (for example, by using quantile-based representations of distributions, or using conditional-value-at-risk as an optimistic summary). The paper's impact and utility for these future works could be increased with some indication that other methodological choices are possible, and perhaps more discussion of why the choices made in this paper are reasonable first choices to study.

_Unclear derivations/possible inaccuracies_

Reward discretization. In the introduction (Line 111), the authors mention discretization of rewards as a property of their framework in line with previous literature, citing Dabney et al.'s work on dopaminergic modelling. However, in this cited work, as in the vast majority of distributional RL literature, rewards themselves are typically not discretized. In general, returns may be discretized with a categorical projection (see below), or some other encoding scheme may be used; Dabney et al. study a model in which a discrete set of expectiles are learnt. From my reading, I think the current paper over-emphasizes how commonplace reward discretization is in distributional RL, and the paper would benefit from clarification here.

Eqn (5): I don't think this Bellman equation holds for Q_t, since Q_t is in general an arbitrary estimate of the Q-function.

Eqn (11): I don't think the move from the first line to the second line is valid. I think it relies on a Bellman equation being satisfied by Q_{X,t}, but I don't believe one holds, because Q_{X,t} is just an approximation to Q_X.

I don't understand the discussion in Section 2.3. The definition of w at Lines 354-356 seems to have an unbounded v variable on the right-hand side. The inequalities here seem to be comparing a return outcome (r_k) to a probability (Q^{i,s,v}_X). I expect there may be a typo here.

_Non-standard distributional RL algorithms_

One important deviation from the distributional RL literature is that this paper describes discretizing reward distributions. In distributional RL, it is typically return distributions that are discretized (as in e.g. Bellemare et al., 2017), rather than reward distributions. Further, rather than the nearest-neighbor discretization described here, which allocates each reward to the neuron corresponding to the closest value, categorical distributional RL methods typically use a projection that preserves the mean (see for example Eqn (7) of Bellemare et al., 2017, or Eqn (7) of Rowland et al., 2018).

There are therefore two important differences from standard categorical distributional RL algorithms: discretization of reward, rather than return, and discretization using a nearest-neighbor projection, rather than a mean-preserving projection. The latter may be quite important to the conclusions of the paper, since reward discretization is a hypothesized mechanism for several observations reported in the paper.

In addition, the idea of using a Gaussian smoothing kernel in the context of distributional RL is explored by Farebrother et al. (2024), who in turn took this approach from Imani and White (2018) who used it in the context of supervised learning. This relates more generally to the concept of label smoothing in machine learning and statistics; some discussion of this prior work would be helpful in contextualizing the contributions of the paper.

I don't think it's necessary for the paper to align all algorithmic choices with the most commonly used ones in distributional RL in the context of machine learning, but some discussion about where deviations are made would aid the clarity of the paper.

_Experiments_

Section 3.1.

This is introduced as an exploration/exploitation challenge, but the agents are not evaluated on a metric that introduces such a trade-off (a classical example would be regret minimization, or comparison of cumulative reward). Instead, the policy at convergence is compared. This could be clarified by de-emphasizing the notion of exploration/exploitation in the discussion in this section.

"DDRL closely approximates the behavior of an asymmetric RL agent". As the authors mention, from Fig 2B-C (and the proof in the supplementary material), it looks like there is exact agreement here. As a general comment (not necessary to address in revisions), it would be interesting to know if there is exact agreement in the dynamics of the two approaches, or just in their limiting strategies.

Section 3.2.

In Section 3.2, some specific values are given for the Stag-Hunt rewards. Why are these specific values used? Do they have precedent in the literature?

Section 3.3

I found the bistability that arises here very interesting. My understanding is that this is one of the benefits of studying the deterministic dynamics of the learning algorithm; the concepts of stability are much easier to pin down than if we were dealing with stochastic algorithms (e.g. incorporating randomness from the agent's policy), because certain conditions are required to guarantee convergence in these cases. If my understanding is correct, it could be nice to emphasize this point here, and when introducing the deterministic dynamics perspective earlier in the paper, to communicate to readers why these choices are made.

The point in this section about discretization of the reward signal playing a key role in stability, in reference to Fig 4E, was not clear to me. As far as I can tell, the number of distributional predictions, and degree of discretization, is not varied in this experiment, so I am unsure how this conclusion is arrived at. It would be great if the authors can expand on this.

__Detailed notes__

_Optional/minor formatting notes_

Using superscript on discount factors to index by agent, as well as for exponents, could cause confusion. Does this paper require the introduction of different discount factors for each agent? I don't believe so, and in this case, Section 2.1.1 could introduce stochastic games with a global discount factor and avoid introducing this notation.

The formatting of Line 140 seems odd. Is there a full stop after ">" which is missing/obscured by the line number?

Section 2 is very long, and contains both review material and new contributions. The authors could consider splitting this section into a few smaller sections, making clearer the distinction between new and existing material.

Line 332: "Noting" -> "Denoting".

_Citations_

In providing references for distributional reinforcement learning, the authors could consider citing earlier work than Bellemare et al. (2017) as well, such as Morimura et al. (2010a;b).

_More minor technical notes_

This section notes technical issues with the presentation, though ones which do not affect the key messages of the paper.

Eqn (7): Based on the conventions of the paper, the R^{s,a} term is missing a factor of (1-gamma^i).

"We assume an agent has n ∈ N reward ”neurons”, each neuron being sensitive to a given range of amount of reward." Should "reward" be "return"?

I don't understand the discussion prior to Eqn (23) that the critic can be removed in the definition of the temporal-difference error. If we remove the critic, it seems that what we are measuring is no longer an error between predicted and realized returns?

Eqn (24): Why not use "V", rather than "Q", on the left-hand side, since this is an action-independent quantity?

Line 436:

"However, if both agents defect, they receive the sucker’s payoff S"

Shouldn't this be

"However, if both agents defect, they receive the punishment payoff P"?

_Terminology_

The manuscript makes use of the term "value distributions" to refer to the distributions learned in the paper. While Bellemare et al. (2017) make use of this term, earlier and later work both use the more technically correct "return distribution".

__References__

Borkar, V.S, 2008. Stochastic approximation: a dynamical systems viewpoint. Cambridge University Press.

Dabney, W., Ostrovski, G., Silver, D. and Munos, R., 2018b. Implicit quantile networks for distributional reinforcement learning. In Proceedings of the International Conference on Machine Learning.

Farebrother, J., Orbay, J., Vuong, Q., Taïga, A.A., Chebotar, Y., Xiao, T., Irpan, A., Levine, S., Castro, P.S., Faust, A. and Kumar, A., 2024. Stop regressing: Training value functions via classification for scalable deep RL. In Proceedings of the International Conference on Machine Learning.

Imani, E. and White, M., 2018. Improving regression performance with distributional losses. In Proceedings of the International Conference on Machine Learning.

Morimura, T., Sugiyama, M., Kashima, H., Hachiya, H. and Tanaka, T., 2010a. Nonparametric return distribution approximation for reinforcement learning. In Proceedings of the International Conference on Machine Learning.

Morimura, T., Sugiyama, M., Kashima, H., Hachiya, H. and Tanaka, T., 2010b. Parametric return density estimation for reinforcement learning. In Proceedings of the Conference on Uncertainty in Artificial Intelligence.

Rowland, M., Bellemare, M., Dabney, W., Munos, R. and Teh, Y.W., 2018. An analysis of categorical distributional reinforcement learning. In Proceedings of the International Conference on Artificial Intelligence and Statistics.

J. N. Tsitsiklis and B. Van Roy, 1997. An analysis of temporal-difference learning with function approximation.In Proceedings of the IEEE Transactions on Automatic Control.

Reviewer #2: Review uploaded as an attachment.

**********

**Have the authors made all data and (if applicable) computational code underlying the findings in their manuscript fully available?**

The PLOS Data policy requires authors to make all data and code underlying the findings described in their manuscript fully available without restriction, with rare exception (please refer to the Data Availability Statement in the manuscript PDF file). The data and code should be provided as part of the manuscript or its supporting information, or deposited to a public repository. For example, in addition to summary statistics, the data points behind means, medians and variance measures should be available. If there are restrictions on publicly sharing data or code —e.g. participant privacy or use of data from a third party—those must be specified.

Reviewer #1: Yes

Reviewer #2: Yes

**********

PLOS authors have the option to publish the peer review history of their article (what does this mean?). If published, this will include your full peer review and any attached files.

If you choose “no”, your identity will remain anonymous but your review may still be made public.

**Do you want your identity to be public for this peer review?**  For information about this choice, including consent withdrawal, please see our Privacy Policy.

Reviewer #1: No

Reviewer #2: No

[NOTE: If reviewer comments were submitted as an attachment file, they will be attached to this email and accessible via the submission site. Please log into your account, locate the manuscript record, and check for the action link "View Attachments". If this link does not appear, there are no attachment files.]

**Figure resubmission:**

**Reproducibility:**

?>

Attachment Submitted filename: 2026.02.16 - PCOMPBIOL-D-25-02733 - comments.pdf

# Author comment: Author response to Decision Letter 1

**DOI:** 10.1371/journal.pcbi.1014723.r002

27 May 2026

Attachment Submitted filename: DistRL-R1v2-ResponseLetter.pdf

# Peer review: Decision Letter 1

**DOI:** 10.1371/journal.pcbi.1014723.r003

**Authors:** Ulrik Beierholm, Andrea E. Martin

2 Jul 2026

PCOMPBIOL-D-25-02733R1

Deterministic dynamics of distributional multi-agent reinforcement learning

PLOS Computational Biology

Dear Dr. Barfuss,

Thank you for submitting your manuscript to PLOS Computational Biology. After careful consideration, we feel that it has merit but does not fully meet PLOS Computational Biology's publication criteria as it currently stands. Therefore, we invite you to submit a revised version of the manuscript that addresses the points raised during the review process.

While the reviewers were happy with the overall progress of the paper, reviewer 1 raised some valid concerns which require minor corrections.

Please submit your revised manuscript by Sep 01 2026 11:59PM. If you will need more time than this to complete your revisions, please reply to this message or contact the journal office at ploscompbiol@plos.org. When you're ready to submit your revision, log on to https://www.editorialmanager.com/pcompbiol/ and select the 'Submissions Needing Revision' folder to locate your manuscript file.

Please include the following items when submitting your revised manuscript:

* A letter that responds to each point raised by the editor and reviewer(s). You should upload this letter as a separate file labeled 'Response to Reviewers'. This file does not need to include responses to formatting updates and technical items listed in the 'Journal Requirements' section below.

* A marked-up copy of your manuscript that highlights changes made to the original version. You should upload this as a separate file labeled 'Revised Manuscript with Track Changes'.

* An unmarked version of your revised paper without tracked changes. You should upload this as a separate file labeled 'Manuscript'.

If you would like to make changes to your financial disclosure, competing interests statement, or data availability statement, please make these updates within the submission form at the time of resubmission. Guidelines for resubmitting your figure files are available below the reviewer comments at the end of this letter.

As the corresponding author, your ORCID iD is verified in the submission system and will appear in the published article. PLOS supports the use of ORCID, and we encourage all coauthors to register for an ORCID iD and use it as well. Please encourage your coauthors to verify their ORCID iD within the submission system before final acceptance, as unverified ORCID iDs will not appear in the published article. Only the individual author can complete the verification step; PLOS staff cannot verify ORCID iDs on behalf of authors.

We look forward to receiving your revised manuscript.

Kind regards,

Ulrik R. Beierholm

Academic Editor

PLOS Computational Biology

Andrea E. Martin

Section Editor

PLOS Computational Biology

**Additional Editor Comments (if provided):**

**Reviewers' comments:**

Reviewer's Responses to Questions

**Comments to the Authors:**

**Please note here if the review is uploaded as an attachment.**

Reviewer #1: I thank the authors for their detailed response and updates in light of the reviews. This review first responds to the authors' letter (unless otherwise noted, I'm happy that the revisions made by the authors have dealt with the points raised in my initial review), and then provides further comment on the revised manuscript as a whole.

_Comment 1.2_

Thank you for the updates here. I have several comments in response; these are all reasonably minor/low-level technical comments, save for the point about reward vs. return distribution discretization, which I think is important to the overall message of the manuscript.

Boltzmann policies.

I think the connection with the maximum-entropy principle is more subtle than the new paragraph implies. Specifically, I don't think the sentence "Among all distributions over actions consistent with current Q-value beliefs, it maximizes entropy" is accurate (or the term "consistent" should be explained in more detail). I think the correct claim is that the Boltzmann distribution can be derived as the distribution over actions that maximizes expected return (according to the Q-function) subject to a constraint on entropy (or alternatively, maximizes entropy subject to a constraint on the expected return (according to the Q-function). It may be that I am misinterpreting the authors' claim here, so potentially further explanation of the claim would also suffice. I am also unsure about the claim that this is the "least arbitrary choice", since it still requires the selection of the coefficient beta^i.

Return-distribution representation.

I'm not sure about the argument that quantile-/expectile-based approximations have no clear neural analogue. Dabney et al. (2020) argue for an expectile-based representation of return distributions in modelling certain neural phenomena, and in some ways these representations have more in common with the non-distributional setting (in which an expected value is parameterized directly), rather than in the categorical setting parametrizing probabilities, rather than outcomes. To be clear, I think the choice to focus on the categorical representation in this paper is a reasonable one, but I don't agree with the above argument for avoiding quantile/expectile-based approaches; it may be that I'm missing something, however. The authors could adapt this specific claim, or provide more reasoning for it.

Deterministic dynamics.

The additional material here helps justify the choice of dynamics, and I found the mentioned connections to replicator systems interesting. I disagree with the sentence "the stationary distribution of the stochastic system concentrates around the stable fixed points of the deterministic part and spends negligible time near unstable ones". I think qualitatively this expresses the right intuitions, but when exactly this is true (versus when stochasticity is such that convergence is prevented, or when the dynamical system may enter a limit cycle rather than reaching the neighborhood of a stationary point) are subtle questions in general. Particularly given that the ODE method is mentioned in the same paragraph, which aims to carefully analyse these questions mathematically.

_Comment 1.4_

Reward discretization vs. return discretization.

With the authors' scaling of rewards by (1-gamma), I agree that reward and return ranges coincide in this work. However, there remains an algorithmic difference between projecting rewards and projecting returns. The authors state in their response to Comment 1.4 that they have now aligned the manuscript with the convention that returns, not rewards, are discretized, but I think there are still numerous references to "reward discretization" in the text (Line 11 of abstract, and in the main paper, e.g. Lines 54 & 227), and I believe Section 3.1.1 still describes the model implementation as discretizing rewards (in addition to returns; see, e.g., Eqn (14)). Unless I have missed something, this seems like a key issue for future revision.

Gaussian smoothing + nearest neighbor projection.

Thanks for the additional explanations, I agree with the authors' summary here, and the cited connection with Farebrother et al. and Imani & White in the revision.

__General comments in light of the new revision__

Overall, the revisions here mostly resolve the technical queries raised in my first review, though a few queries remain, as discussed above, and including the discrepancy between reward/return discretization, summarized below. I remain of the opinion that this work is interesting, complements existing distributional RL literature in the domains of machine learning and neuroscience, and is likely to lead to further investigation in this area. I think there are still some notational/technical issues to address, as described above, and discuss several broad areas where I think there is room for improvement below.

Framing of contributions.

In reading the comments addressed in the other review, I'd agree that further explanation and emphasis of the key aims of the paper would be helpful. For example, at the end of the revised introduction, it is stated that "we validate the resulting learning dynamics"; it could help to more clearly state what the goals of the modeled dynamics are, and what it means to validate them. My understanding is the goal of the dynamics/model is not to have a realistic model of a multi-agent learning system (since this would typically involve stochasticity in agents' actions, imperfectly estimated value functions, etc.), but to have a simpler model that nevertheless provides mathematical predictions of phenomena in multi-agent systems that can be validated against observation. Additionally, my understanding is that the proposals here are not algorithmic proposals for agents implementing reinforcement learning (for example, it is often not possible for an agent to compute the expected quantities that feature in these dynamics), but instead are proposals for methods of analysis.

Clarity of equations/notations.

I thank the authors for updating these sections in light of my technical comments in the initial review. There are still some concerns around clarity:

- The index t appears to be used with two different semantics. In e.g. Eqn (2), it indexes both states/actions, and estimated quantities like Q-functionsm implying that it refers to time within an MDP episode. However, around Eqn (10), it is not clear whether this is still the case (the states/actions are not t-indexed here), and the indexing of the policy X_t implies this may no longer be time steps in the game, but instead discrete time steps in the discretiszd dynamics.

- Similarly, it is unclear at Eqn (11) whether this means a single action a^i at a single state s is taken at time t, and so Eqn (11) applies to update X_t only for these coordinates, or if the update is intended to apply to all state-actions pairs.

Discrepancy from standard distributional RL algorithms.

This concern was raised in the original review: namely, that there are certain choices in defining the distributional RL methods in the paper that differ from standard choices in the distributional RL literature, such as the discretization of rewards, rather than returns (the authors have responded to this query raised in the original review; see the response to Comment 1.4 above for more technical details and remaining queries). I think currently, the discrepancy between reward discretization (the approach taken in this paper) and return discretization (the approach broadly taken in the distributional RL literature) is not sufficiently discussed; I think this difference isn't necessarily a problem per se, but this could perhaps be made more prominent in the text, since from what I understand this specific design decision is crucial to the predictions that emerge from the model, see e.g. Line 675: "Reward discretization thus generates a hierarchy of path-dependent phenomena–from choice hysteresis, to escapable perseveration", and so the conclusions reached in the paper may be somewhat specific to this non-standard choice of algorithmic specification.

_General minor typos etc._

There are several typos/notational points that could be addressed. A non-exhaustive list is given below:

Line 255: "[60, 61]and" is missing a space.

Line 63: "cartesian product" -> "Cartesian product"

alpha is overloaded as a step size and part of the optimism parametrization.

Eqn (15): sum_{\alpha^j} should be sum_{alpha^{-i}} I think.

Around Line 154, we have both r_t (the reward at time t), and r_n (the nth reward atom location).

Line 264: There is an unbound t on RHS of new "G =" equation.

Reviewer #2: I thank the authors for thoroughly addressing my points. I do not have any more concern.

**********

**Have the authors made all data and (if applicable) computational code underlying the findings in their manuscript fully available?**

The PLOS Data policy requires authors to make all data and code underlying the findings described in their manuscript fully available without restriction, with rare exception (please refer to the Data Availability Statement in the manuscript PDF file). The data and code should be provided as part of the manuscript or its supporting information, or deposited to a public repository. For example, in addition to summary statistics, the data points behind means, medians and variance measures should be available. If there are restrictions on publicly sharing data or code —e.g. participant privacy or use of data from a third party—those must be specified.

Reviewer #1: None

Reviewer #2: Yes

**********

PLOS authors have the option to publish the peer review history of their article (what does this mean?). If published, this will include your full peer review and any attached files.

If you choose “no”, your identity will remain anonymous but your review may still be made public.

**Do you want your identity to be public for this peer review?**  For information about this choice, including consent withdrawal, please see our Privacy Policy.

Reviewer #1: No

Reviewer #2: No

[NOTE: If reviewer comments were submitted as an attachment file, they will be attached to this email and accessible via the submission site. Please log into your account, locate the manuscript record, and check for the action link "View Attachments". If this link does not appear, there are no attachment files.]

**Figure resubmission:**

**Reproducibility:**

To enhance the reproducibility of your results, we recommend that authors of applicable studies deposit laboratory protocols in protocols.io, where a protocol can be assigned its own identifier (DOI) such that it can be cited independently in the future. Additionally, PLOS ONE offers an option to publish peer-reviewed clinical study protocols. Read more information on sharing protocols at https://plos.org/protocols?utm_medium=editorial-email&utm_source=authorletters&utm_campaign=protocols

# Author comment: Author response to Decision Letter 2

**DOI:** 10.1371/journal.pcbi.1014723.r004

29 Jul 2026

Attachment Submitted filename: DistRL-R2-ResponseLetter.pdf

# Peer review: Decision Letter 2

**DOI:** 10.1371/journal.pcbi.1014723.r005

**Authors:** Ulrik Beierholm, Andrea E. Martin

17 Aug 2026

Dear Barfuss,

We are pleased to inform you that your manuscript 'Deterministic dynamics of distributional multi-agent reinforcement learning' has been provisionally accepted for publication in PLOS Computational Biology.

Before your manuscript can be formally accepted you will need to complete some formatting changes, which you will receive in a follow up email. A member of our team will be in touch with a set of requests.

Please note that your manuscript will not be scheduled for publication until you have made the required changes, so a swift response is appreciated.

IMPORTANT: The editorial review process is now complete. PLOS will only permit corrections to spelling, formatting or significant scientific errors from this point onwards. Requests for major changes, or any which affect the scientific understanding of your work, will cause delays to the publication date of your manuscript.

Should you, your institution's press office or the journal office choose to press release your paper, you will automatically be opted out of early publication. We ask that you notify us now if you or your institution is planning to press release the article. All press must be co-ordinated with PLOS.

Thank you again for supporting Open Access publishing; we are looking forward to publishing your work in PLOS Computational Biology.

Best regards,

Ulrik R. Beierholm

Academic Editor

PLOS Computational Biology

Andrea E. Martin

Section Editor

PLOS Computational Biology

***********************************************************

Reviewer's Responses to Questions

**Comments to the Authors:**

**Please note here if the review is uploaded as an attachment.**

Reviewer #1: I thank the authors for their further revisions to the manuscript and responses to my previous comments. I'm satisfied with the authors' responses on these points, and would be supportive of the publication of the manuscript. Below I have included some commentary on the authors' latest revisions, but to be clear I am not raising anything that I think needs further revision.

I think the additional discussion on the framing of contributions and the motivations behind the modelling framework has improved the clarity of the introduction, and the overall narrative of the paper.

Thanks also for the updates on notation, and clarifications where my comments arose from a misunderstanding on my side.

Comment 1.5 & Comment 1.10

Thanks for the additional response here. One of the main concerns I raised in the initial review, which has been the subject of some discussion subsequently, was the differences between the distributional RL algorithm described here, and the implementations used commonly in machine learning. This centered on the discretization of the reward employed, which differs from the usual Cramer projection typically used in machine learning approaches. I appreciate the authors' additional comments on this point, and I think the difference is now made clear in the paper, so I am happy on this point. An interesting question for future work could be to investigate any potential sensitivity to choices like this in the framework.

Comment 1.9

Thanks for the clarification on the semantics of the index t. While some presentations of reinforcement learning/dynamic programming algorithms use distinct indices for "time along a trajectory" (commonly t) and "algorithm time" (often k, see e.g. Eqn (4.10) of Sutton and Barto (2018)), I think with the additional text the authors have added to the paper, the current presentation is clear.

**********

**Have the authors made all data and (if applicable) computational code underlying the findings in their manuscript fully available?**

The PLOS Data policy requires authors to make all data and code underlying the findings described in their manuscript fully available without restriction, with rare exception (please refer to the Data Availability Statement in the manuscript PDF file). The data and code should be provided as part of the manuscript or its supporting information, or deposited to a public repository. For example, in addition to summary statistics, the data points behind means, medians and variance measures should be available. If there are restrictions on publicly sharing data or code —e.g. participant privacy or use of data from a third party—those must be specified.

Reviewer #1: None

**********

PLOS authors have the option to publish the peer review history of their article (what does this mean?). If published, this will include your full peer review and any attached files.

If you choose “no”, your identity will remain anonymous but your review may still be made public.

**Do you want your identity to be public for this peer review?**  For information about this choice, including consent withdrawal, please see our Privacy Policy.

Reviewer #1: No

# Editor report: Acceptance letter

**DOI:** 10.1371/journal.pcbi.1014723.r006

**Authors:** Ulrik Beierholm, Andrea E. Martin

PCOMPBIOL-D-25-02733R2

Deterministic dynamics of distributional multi-agent reinforcement learning

Dear Dr Barfuss,

I am pleased to inform you that your manuscript has been formally accepted for publication in PLOS Computational Biology. Your manuscript is now with our production department and you will be notified of the publication date in due course.

The corresponding author will soon be receiving a typeset proof for review, to ensure errors have not been introduced during production. Please review the PDF proof of your manuscript carefully, as this is the last chance to correct any errors. Please note that major changes, or those which affect the scientific understanding of the work, will likely cause delays to the publication date of your manuscript.

Soon after your final files are uploaded, unless you have opted out, the early version of your manuscript will be published online. The date of the early version will be your article's publication date. The final article will be published to the same URL, and all versions of the paper will be accessible to readers.

For Research, Software, and Methods articles, you will receive an invoice from PLOS for your publication fee after your manuscript has reached the completed accept phase. If you receive an email requesting payment before acceptance or for any other service, this may be a phishing scheme. Learn how to identify phishing emails and protect your accounts at https://explore.plos.org/phishing.

Thank you again for supporting PLOS Computational Biology and open-access publishing. We are looking forward to publishing your work!

With kind regards,

Sharmila Kamatchi

PLOS Computational Biology | Carlyle House, Carlyle Road, Cambridge CB4 3DN | United Kingdom ploscompbiol@plos.org | Phone +44 (0) 1223-442824 | ploscompbiol.org | @PLOSCompBiol
